# Mild, Organo-Catalysed Borono-Deamination as a Key to Late-Stage Pharmaceutical Precursors and ^18^F-Labelled Radiotracers

**DOI:** 10.3389/fchem.2022.884478

**Published:** 2022-04-26

**Authors:** Raúl M. Pérez-García, Patrick J. Riss

**Affiliations:** ^1^ Section of Organic Chemistry, Department of Chemistry, University of Oslo, Oslo, Norway; ^2^ Division of Clinical Neuroscience, Oslo University Hospitals HF, Oslo, Norway; ^3^ GIGA Cyclotron Research Centre, Department of Chemistry, Liège, Belgium; ^4^ Department of Chemistry, Johannes Gutenberg-University, Mainz, Germany

**Keywords:** aromatic amines, boronato-deamination, aryl boronates, lewis acid-catalysis, drug molecules, radiofluorination, positron emission tomography

## Abstract

A tris(pentafluorophenyl)borane catalysed method for the synthesis of boronic acid esters from aromatic amines in yields of up to 93% was devised. Mild conditions, benign reagents, short reaction times, low temperatures and a wide substrate scope characterize the method. The reaction was found applicable to the synthesis of boronic acid ester derivatives of complex drug molecules in up to 86% isolated yield and high purity suitable for labelling. These boronates were subsequently labelled with [^18^F]fluoride ion in radiochemical yields of up to 55% with and even without isolation of the boronate-intermediate.

## 1 Introduction

The labeling of pharmaceuticals with short-lived, positron-emitting radionuclides (t_1/2_ < 2 h) is a prerequisite for their use as positron emission tomography (PET) radiotracers. Such short half-life require the introduction of radioactive isotopes like carbon-11 (t_1/2_ = 20 min) or fluorine-18 (t_1/2_ = 110 min) into suitable precursors in the last stages of synthesis. Typical labelling precursors are structurally complex drug molecules bearing a diverse array of functional groups with defined stereochemistry, which must be conserved through all subsequent chemical transformations to retain the desired pharmacodynamic properties. Moreover, these precursors have to be synthesized with a labelling strategy in mind, which is compatible with effective synthesis routes and free of conflicting functional groups (Serdons et al., 2009; Krishnan et al., 2017). Given the ever-growing need for radiolabeled tracers in biomedical research, robust access to precursors has become a critical bottleneck to facilitate labelling. Radiolabeling with fluorine-18 is only practically useful for tracer studies if high quality [^18^F]fluoride ion is used to obtain highly radioactive products suitable for tracer studies. In contrast to simple nitro-precursors for nucleophilic aromatic fluorination of activated arenes, non-activated arenes may require alternative precursors. Hypervalent iodanes, iodonium ylids and Pd-complexes have been explored for the purpose but were clearly found less compatible with typical drug molecules, both in terms of precursor synthesis and labelling (Serdons et al., 2009; Riss et al., 2012a; Huiban et al., 2013; Colem et al., 2014; Rühl et al., 2014; van der Born et al., 2014; Krishnan et al., 2017; Jana et al., 2020). The underlying chemistry may require protection of functional groups or involve complicated methods for their synthesis (Colem et al., 2014). Aryl boronic acid and aryl boronic acid ester precursors have received special attention in the last decade, mainly due to the advent of relatively versatile copper mediated labelling strategies with [^11^C]CO_2_, [^18^F]CF_3_I, [^18^F]fluoride ion or Cu [^18^F]CF_3_. The use of copper offers a wide variety of labelling methods compatible with the requirements of good manufacturing practice and release criteria for application of the products in man, particularly due to the relatively high limit for daily Cu exposure of 350 µg/day (Riss et al., 2012b; Huiban et al., 2013; Rühl et al., 2014; Tredwell et al., 2014; van der Born et al., 2014; Mossine et al., 2015; Preshlock et al., 2016; Zischler et al., 2017; Clemente et al., 2019; Jana et al., 2020; Mossine et al., 2020; Wright et al., 2020; Yuan et al., 2020). In consequence, these methods have been adapted to automated radiosynthesis for tracer production, which sets the pace for widespread use in clinical PET imaging studies.

In our efforts to synthesize precursors for the radiosynthesis of the PolyADP-ribose polymerase (PARP) inhibitor [^18^F]Rucaparib, we devised a deamination strategy to obtain a labelling precursor (Riss et al., 2012a; Alluri and Riss, 2018; Peréz-García et al., 2021). Methods for C-N borylation based on thermal degradation or metal mediated mechanisms with diazonium intermediates have been introduced to exploit the ample variety of accessible nitrogen compounds and their utility as intermediates in synthesis (Fang et al., 2004; Bej et al., 2012; Kundu et al., 2012; Qiu et al., 2013; Chen et al., 2020; Shegavi et al., 2020; Wang and Shi, 2020). Borono-deamination has so far been used as a robust means to provide small molecule building blocks for further use in synthesis (Mo et al., 2010; Qiu et al., 2014). However useful small aromatic building blocks may be, our challenge was to derive a method for use on diverse drug-like molecules to prepare labelling precursors. Ideally a mild, one-pot borylation suitable to introduce boronic esters stereospecifically into typical advanced pharmaceutical compounds under future-proof, environmentally benign conditions. In addition to mild conditions and a broad functional group tolerance, the use of metal catalysts was not desired, to circumvent recovery of heavy metals used for this purpose (Bej et al., 2012; Kundu et al., 2012; Shegavi et al., 2020). The common problems of undesired cross reactivities like C-C coupling with haloarenes or Chan-Lam reactions was another issue associated with available boronation protocols (Qiu et al., 2013; Chen et al., 2020).

## 2 Results and Discussion

We began our investigation on the borono-deamination of anilines (Table 1) using 1.2 equivalents of bis(pinacolato)diboron (B_2_pin_2_), 1.5 equivalents of *tert*-butyl nitrite and 4-nitroaniline **1** as a model compound and determining yields of 4-nitrophenylboronic acid ester **2** by ^1^H NMR spectroscopy. Initial tests produced only 49.8% yield of the borylated compound in addition to 4-nitrobenzene **3** (proto-deaminated analogue) as the main products of the reaction. In general, benzene formation was observed as the major side reaction but tended to be lower in aprotic solvents of low polarity and in absence of Lewis acids. Unreacted diazonium salt was a major side product in CH_2_Cl_2_ and 2,2-dimethylpropanol (entries 1 and 7). In the solvent screen, MeCN was shown to be the best solvent for the reaction, particularly when changing the nitrous acid ester to amyl nitrite (AmylONO), which gave 65.5% yield (entry 5). MeCN is considered most green amongst the dipolar aprotic solvents (Byrne et al., 2016; Prat et al., 2016) and is easy and safe to handle under common HSE guidelines. As a byproduct of bulk acrylonitrile monomer production and a preferred (U)HPLC solvent, MeCN will remain available and in use for decades to come.

**TABLE 1:** Effect of solvent on the formation of products.

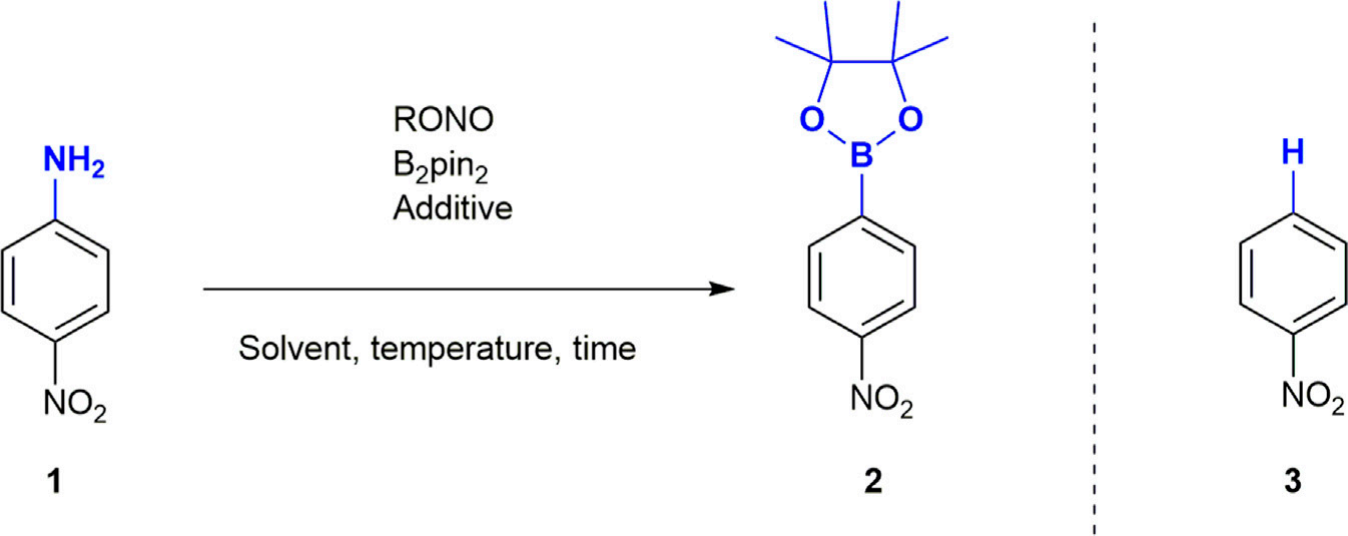					
Entry (substrate)	Solvent	Yield/%^a^			
		2	3	*r* = 2/3	ArN_2_^+^
1 (**1**)	CH_2_Cl_2_	34.9	9.0	3.9	17.4
2 (**1**)	DMF	9.4	32.3	0.3	0.0
3 (**1**)	DMSO	41.1	16.2	2.5	5.6
4 (**1**)^b^	MeCN	49.8	11.2	4.4	2.7
5 (**1**)	MeCN	65.5	16.8	3.9	1.7
6 (**1**)	MeOH	7.7	0.3	25.6	trace
7 (**1**)	2,2-Me_2_-PrOH	43.9	14.5	3.0	32.8
8 (**1**)	THF	15.8	23.3	0.7	20.9
9 (**1**)	PhMe	14.7	4.3	3.4	0.0

Conditions: 1 (72 μmol), B_2_pin_2_ (1.2 Eq), MeCN (360 μL) AmylONO (1.5 equiv.), 80°C, 3 h.

a Yields determined by NMR, spectroscopy.

b Tert-Butyl nitrite (1.5 equiv.) was used.

Since some radicals are particularly long lived in MeCN (Warren and Mayer, 2008) direct protonation by solvent H emerges as a possible mechanism behind undesired benzene formation (Cohen et al., 1977). This further corroborated by the fact that the reaction can be promoted by catalytic amounts of radical initiators (Mo et al., 2010). We aimed to avoid radical promoted mechanisms to minimise potential side reactions with drug molecules. In addition, the alternative pathway of proto-deborylation of the product over time can also lead to benzenes as we were about to confirm in our study. As seen in Table 1, entry 5, MeCN showed the highest borylation to protonation ratio, which bodes well for its use in the reaction. With a solvent of choice established, we reasoned that a Lewis acid in catalytic amounts may promote the reaction (Table 2) (De Vries et al., 2012; Ingleson, 2012), reduce reaction temperature and time in order to suppress proto-deborylation.

**TABLE 2:** Effect of Lewis acids on the product spectrum.

Entry (substrate)	LA (mol%)	Yield/%^a^			
		2	3	r = 2/3	ArN2+
1 (**1**)	AlCl_3_ (5)	57.7	11.2	5.1	2.3
2 (**1**)	AlF_3_ (5)	66.8	16.0	4.1	2.0
3 (**1**)	BBr_3_ (5)	56.7	9.1	6.2	4.7
4 (**1**)	BCl_3_ (5)	50.0	9.9	5.0	4.0
5 (**1**)	BF_3_.Et_2_O (20)	55.2	8.5	6.5	3.0
6 (**1**)	B(C_6_F_5_)_3_ (10)	53.7	10.3	5.2	2.4
7 (**1**)	B(C_6_F_5_)_3_ (2.5)	67.0	14.7	4.5	3.0
8 (**1**)	B(C_6_F_5_)_3_/PPh_3_ (2.5/2.5)	60.8	13.9	4.3	1.2
9 (**1**)	BPh_3_ (5)	67.8	16.1	4.2	9.8
10 (**1**)	B(O*i*Pr)_3_	60.7	16.4	3.7	2.1
11 (**1**)	MgCl_2_ (5)	57.9	16.8	3.4	2.8
12 (**1**)	MgF_2_ (5)	56.6	12.1	4.6	4.3
13 (**1**)	Yb(OTf)_3_ (5)	54.7	13.6	4.0	2.4
14 (**1**)	Yb(OTf)_3_ (2.5)	57.6	15.2	3.8	3.1
15 (**1**)	ZnCl_2_ (5)	46.2	7.7	6.0	16.9
16 (**1**)	ZnCl_2_ (2.5)	55.4	16.3	3.4	4.4
17 (**1**)^b^	AlF_3_ (5)	86.0	3.8	22.6	1.3
18 (**1**)^b^	AlF_3_ (10)	86.1	4.7	18.3	1.7
19 (**1**)^b^	B(C_6_F_5_)_3_ (5)	86.5	3.0	28.8	1.3
20 (**1**)^b^	B(C_6_F_5_)_3_ (2.5)	90.5	4.5	20.1	1.3
21 (**1**)^b^ ^,^ ^c^	B(C_6_F_5_)_3_ (2.5)	88.3	7.17	12.3	0.9
22 (**1**)^b^	none	71.3	12.9	5.5	8.5
23 (**1**)^d^	none	55.5	9.3	5.9	24.1
24 (**1**)^d^	B(C_6_F_5_)_3_ (2.5)	84.0	3.1	27.1	1.5

Conditions: 1a (72 μmol), B_2_pin_2_ (1.2 Eq), MeCN (360 μL) AmylONO (1.5 equiv.), 80°C, 3 h.

a Yields determined by NMR, spectroscopy.

b Reaction with B_2_pin_2_ (4 Eq), 40°C, 15 min.

c B_2_neop_2_ (4 Eq) used as diboron reagent; LA, lewis acid.

d Reaction with B_2_pin_2_ (4 equiv.), 25°C, 15 min.

**FIGURE 1:**
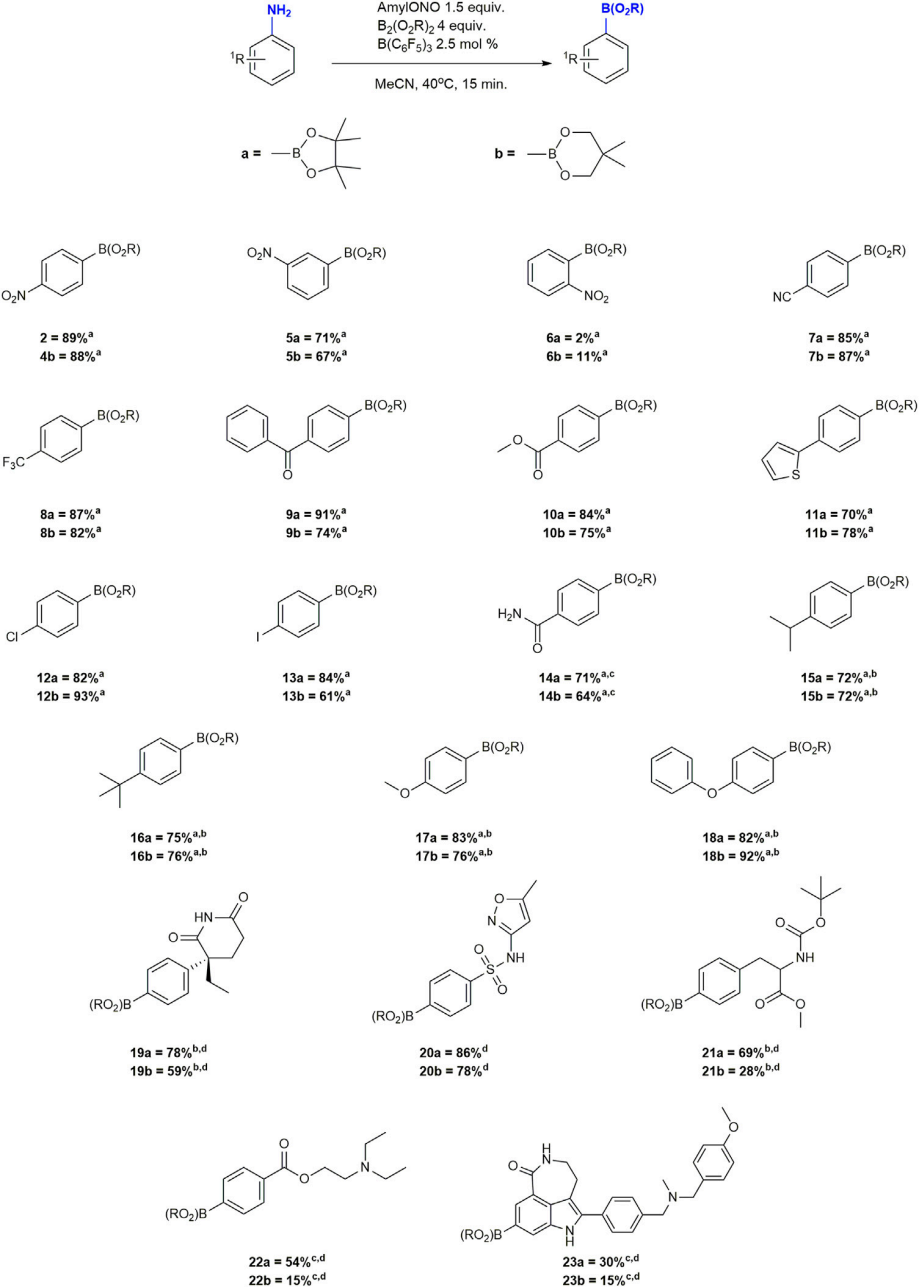
Substrate scope of the borono-deamination reaction. ^a^Yield determined by ^1^H-NMR spectroscopy. ^b^Reaction performed at 80°C. ^c^AcOH (4 equiv.) added as an additive. ^d^Isolated yield.

Thus, we proceeded to test the hypothesis by screening a panel of Lewis acids. Selected results from a screen over 2.5–20 mol%, temperatures between 25 and 80°C and 15–180 min reaction time are shown in Table 2. We were pleased to find that Lewis acid catalysis indeed promoted the reaction producing two distinct outcomes. As a general trend the yield improved markedly in presence of catalysts. In consequence, the reaction was found to proceed at much lower temperatures which further improved the yield and product distribution. In methodological experiments, tris(pentafluorophenyl)borane [B(C_6_F_5_)_3_] was eventually identified as the most effective Lewis acid. The borane produced the highest yield of all employed Lewis acids (Table 2, entries 7, 19–21) while allowing for drastically reducing both the reaction time and the temperature relative to control. Time and temperature dependent experiments revealed that starting material had already been consumed within the first 15 min of the reaction, at 40°C (entries 17–21) with mere traces of the diazonium intermediate left over after 15 min at 25°C (entries 23 and 24). A marked, positive effect from the B(C_6_F_5_)_3_ was demonstrated clearly by a double figured increase of product at 25 and 40°C. In addition to increased yields, a lower tendency of products to proto-deborylate was observed through improvement in the ratio of **2** to **3** (Table 2) to 28.8, which is five-fold higher than control (5.9). Under these conditions no remaining starting material was detected in any of the trials. At this point we surmised that thermal degradation was the preferred reaction pathway at elevated temperature while an organocatalytic route became viable at lower temperatures. Under the assumption of a catalytic mechanism in action with reduced activation energy, lower temperature and shorter reaction times are expected to produce equal or better yield. This was clearly confirmed by both, much higher yield relative to control at lower temperature and shorter reaction time in presence of B(C_6_F_5_)_3_ as well as higher conversion of the available diazonium intermediate (entries 15–21). A synergistic effect of faster conversion and better product selectivity leads to 20–30% higher yield and three- to five-fold lower benzene formation (entries 18–21). Under optimized conditions, a reproducible yield of 89 ± 1% (*n* = 4) was obtained with B_2_pin_2_. We reasoned that a Lewis acid may serve as a reversible activator for the diboron reagent to increase reactivity and, thereby, suppress radical mediated side reactions. In contrast to earlier reports, we do not observe N-arylation as a side reaction and obtain higher yields, which includes high isolated yields on drug molecules, even at much reduced reaction time and temperature. We surmised that a putative activated diboron-Lewis acid complex reacts directly in the borono-deamination reaction and thereby limit the life-time of any possible radical intermediate. This is clearly reflected by near absence of radical protonation product (benzenes) and a much shorter reaction time (15 min), with concomitant increase in yield of up to 50% of the theory. Despite the similarity of the method to the Sandmeyer reaction and to other procedures in the literature (Mo et al., 2010; Qiu et al., 2013; Qiu et al., 2014; Akhtar et al., 2021), we propose a different mechanism which is based on the lewis acid assisted disproportionation of diboron reagent to produce 1 equiv. of product and one equivalent of borate. This hypothesis was supported by detectection of borate by ^1^H-NMR of reaction mixtures. With the best conditions in hand, we proceeded to test the substrate scope of the reaction (Figure 1).

A broad variety of functional groups were tested in the form of substituted anilines in good to excellent yields. In general, changing the boronate source to bis(neopentyl)glycolato diboron (B_2_neop_2_) did not significantly affect product formation. While compound **2**, **4** and **5** were borylated in between 65–89% yield the ortho derivatives **6a** and **6b** gave only 2 and 11% yield of borylated products, respectively, with the major product being the protodeborylation analog. This may indicate a steric effect of bulky boronic acid ester. Arenes containing electron withdrawing functional groups (compounds **2**–**11**) were borylated in over 64% and up to 91% yields. Halogenated compounds **12** and **13** were borylated in 65, 82, 84 and 93% yield. In contrast to earlier reports and controls without catalyst, cross-coupling was not detected, highlighting the chemoselectivity of the method (Kapdi and Prajapati, 2014). In the case of anilines with electron donating groups, yields were initially lower than 50%, surely owed to the stability of the corresponding diazonium compounds which simply did not react fast enough. Since the decomposition of the diazonium intermediates is the rate determining step in these reactions, we surmised that increasing the temperature would, in turn, increase borylation yields in favour over benzene formation with our catalytic system (Cowdery and Davies, 1949; Kochi, 1957; Zollinger, 1973). Indeed increasing the reaction temperature to 80 from 40°C boosted yields of compounds **15–18** two- to three-fold to 72–93%. Compounds **14**, **22** and **23**, were more challenging to borylate. Using the standard conditions returned mostly unreacted aniline, and the amine precursor to compound **23** was not soluble in pure MeCN. The presence of additional basic functional groups in both compounds was found to impede the reaction likely by sequestering the Lewis acid catalyst as yields improved to typical levels when a stoichiometric excess of the catalyst was employed. Addition of 4 equivalents of acetic acid (AcOH) without any other changes to the optimized conditions caused all anilines to dissolve and react smoothly to obtain the same range of double figured yields seen for the other substrates (Figure 1).

Having confirmed a broad substrate scope, we proceeded to the synthesis and isolation of late stage drug compounds of interest for PET imaging studies in our clinical imaging programmes. We selected amines that contained a diverse range of functional groups typically encountered in drug molecules, and preferably with biological activity, to demonstrate the ability of the method to produce boronic acid ester precursors for subsequent radiofluorination (Figure 1). We proceeded with the syntheses of compounds **19a/b**, **20a/b**, **21a/b**, **22a/b** and **23a/b**. However, isolation of products did not come without challenges. Decomposition of products and coelution with excess unreacted diboron reagent on silica gel were major hurdles to obtaining pure compounds. Decomposition in particular was very pronounced in Bneop derivatives. These same challenges with the purification of arylboronic acid esters had already been reported (Roy and Brown, 2007; Hitosugi et al., 2012), but no solution was offered at the time. Seeing the need for a purification strategy we decided to also optimize isolation of Bpin and Bneop derivatives of drug molecules. Gratifyingly we found that the addition of AcOH to mobile phases during chromatography reduced the residency time of the compound on the solid phase and suppressed hydrolysis of the boronate product by OH^−^-nucleophiles on the column. This allowed for isolation of compounds in moderate to very good yields (Figure 1). After optimization of chromatographic conditions, we were even able to recover further unreacted B_2_pin_2_ which was spectroscopically undistinguishable from the material received from commercial suppliers Supplementary Material.

Besides the improved yield and product distribution in combination with a wide scope of suitable substrates, we considered B(C_6_F_5_)_3_ an ideal catalyst for our purpose. Its general ease of handling, safety and chemoselectivity comes with straightforward detection, quantification and purification by HPLC or LCMS. Thus (U)HPLC is suitable to detect residual catalyst in labelling precursors after GMP synthesis prior to release of the batch, which is a valuable feature for clinical use.

**FIGURE 2:**
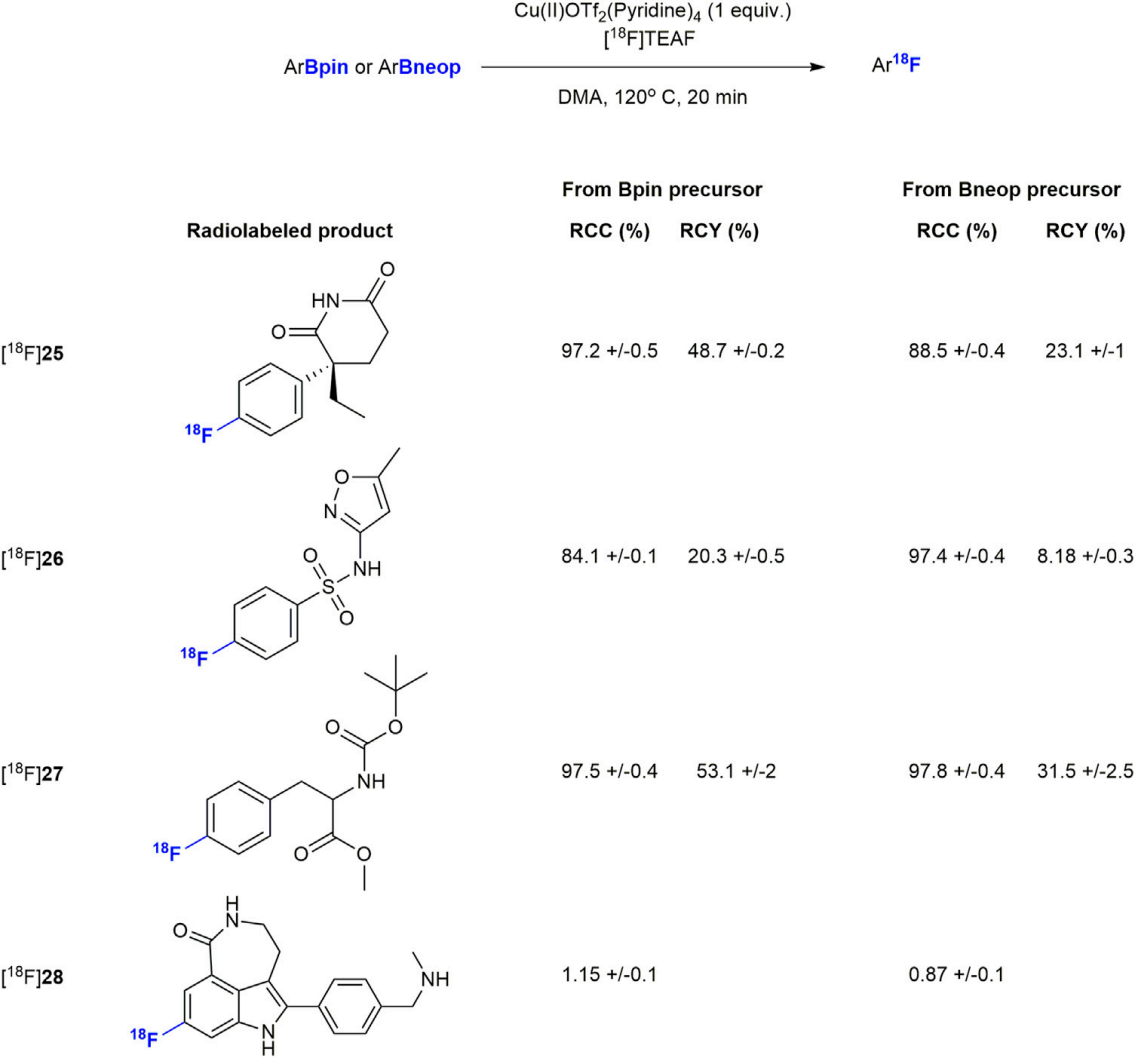
Radiolabeling of drug compounds. Results are all averages of two runs (*n* = 2).

Finally, we subjected the pure boronic acid esters to the copper mediated radiofluorination reaction that motivated our current study. Without optimization, and with a modified literature procedure (Figure 2) (Detailed procedures are available in the Supplementary Material) (Mossine et al., 2020), we were able to radiofluorinate compounds [^18^F]**25-**[^18^F]**28**, which were obtained in radiochemical yields of less than 20%. To optimize radiolabeling conditions (Table 3), we chose conditions from recent publication (Peréz-García et al., 2021) as a starting point for a small study using compound **2** as the substrate. After testing different stoichiometries of copper(I) and copper (II) triflates, one equivalent of tetrakis pyridine copper (II)triflate relative to starting material more than doubled radiochemical conversion (RCC) from 26.2 to 56.2%. Preference for copper (II) indicates a B-Cu-transmetalation followed by reductive elimination upon nucleophilic attack by fluoride ion as the key mechanism, rather than a redox mechanism which would be more likely for Cu(I) (Peréz-García et al., 2021). N,N-dimethylacetamide was identified as the optimal solvent to bring the RCC to 99% and isolated radiochemical yields (RCY) up to 41% at the end of the process. Finally, among the [^18^F]fluoride ion sources tested, isolated RCY increased further to 58% in presence of tetraethyl ammonium bicarbonate (TEAB) while nearly quantitative RCC was maintained. This may be attributed to the softer quarternary ammonium cation, which promotes fluoride transfer to a harder Cu^II/III^ cation. Once fluorinated, it becomes plausible for the Cu(II) to assume a higher oxidation state just ahead of the reductive-elimination-fluorination.

**TABLE 3:** Optimization of radiolabeling reaction with [^18^F]fluoride.

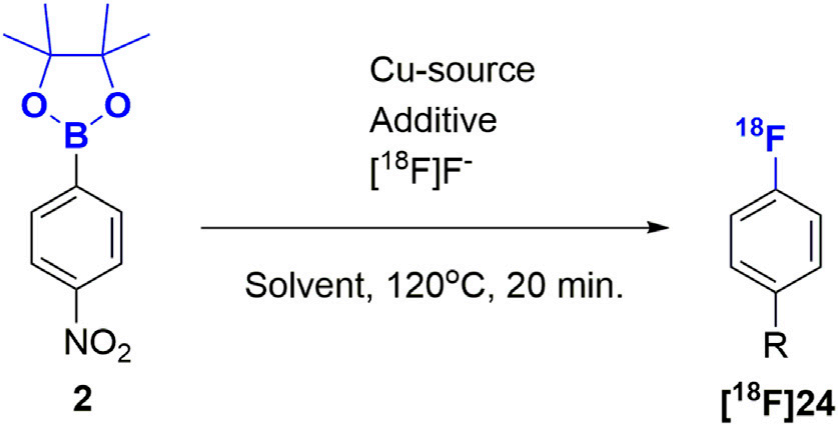				
Copper Salt and Concentration Screen				
Entry (substrate)	Copper Salt (mM)	Additive (mM)	RCC (%)	RCY^a^ (%)
1 (**2**)	Cu(I)OTf (120)	Pyridine (480)	26.2	10.6
2 (**2**)	Cu(II)OTf_2_ (120)	Pyridine (480)	49.9	9.26
3 (**2**)	Cu(I)OTf (30)	Pyridine (120)	5.74	1.91
4 (**2**)	Cu(II)OTf_2_ (30)	Pyridine (120)	51.2	18.7

Solvent screen				
Entry (substrate)	Solvent		RCC (%)	RCY^a^ (%)
4 (**2**)	MeCN		51.2	18.7
5 (**2**)	DMF		98.1	19.5
6 (**2**)	DMSO		50.5	4.79
7 (**2**)	DMA		99.8	40.8
8 (**2**)	2-Methyl-2-butanol		50.3	25.7

Fluoride source screen				
Entry (substrate)	Fluoride source (36 μmol)		RCC (%)	RCY^a^ (%)
9 (**2**)	KOTf/K_222_		98.4	44.8
10 (**2**)	KOTf/18C6		98.2	48.6
7 (**2**)	KOTf/15C5		99.8	40.8
11 (**2**)	TEAB		97.1	58.4
12 (**2**)	TBAOTf		96.6	54.4

Initial solvent and fluoride source were MeCN, and KOTf/15C5 (36 μmol) respectively.

a Isolated radiochemical yield, activity of the isolated product counted by dose calibrator.

With the optimized conditions in hand, we resumed radiolabeling of drug compounds. The results summarized in Figure 2, show excellent RCCs (88–97%) and sufficient to good RCY. Notably all Bneop derivatives labeled in poorer yields than Bpin derivatives, an observation we were able to attribute to increased sensitivity to thermal decomposition. Compounds containing basic amine functional groups labeled poorly (**28a/b**) or not at all (**29a/b**). One explanation may be stronger basicity when compared to pyridine, leading to Cu-complexation which, in turn impedes transmetalation and subsequent fluorination. Compound [^18^F]**28**, was radiolabeled and deprotected with ceric ammonium nitrate (CAN) (Supplementary Material) which resulted in a low but reproducible yield. In addition, we were able to perform the borono-deamination reaction, followed by direct radiofluorination in one pot using the optimal conditions described herein. Initial experiments with excess diboron reagent produced no labelled compound, but reducing the amount of diboron reagent to 0.5 equivalents relative to aromatic amine gave labeled products [^18^F]**25** and [^18^F]**26** in 1 and 17.5% respectively (see Supplementary Material for a detailed procedure). All other radiotracer candidates were radiofluorinated from their boronic acid ester precursors in practically useful RCY of up to 55%, excellent radiochemical purities (RCP) of >95%, and with a copper content 0.05–0.22 μg well below the limit of daily intake of 350 μg (Table 4). Molar activities of 135–327 MBq/nmol (Table 4) were achieved, which is sufficient for their use in PET studies.

**TABLE 4:** Quality control results of radiolabeleld compounds.

Compound	RCP (%)	Starting Activity (MBq)	Cu Content (μg/dose)	A_M_ (MBq/nmol)
[^18^F]**25**	97.2	1101	0.15	327
[^18^F]**26**	99.8	1307	0.22	198
[^18^F]**27**	97.8	1248	0.10	135
[^18^F]**28**	≥95	932	0.5	208

## 3 Conclusion

We have developed a remarkably rapid, mild and widely applicable method for Lewis acid catalyzed borono-deamination at low temperature, suitable for the synthesis of radiolabeling precursors. Borylation and isolation of late stage drug compounds was achieved in moderate to excellent yields, and excess reagents can be recovered from the reaction mixtures with ease. Radiofluorination reaction was optimized, which allowed synthesis of compounds [^18^F]**25**, [^18^F]**26**, [^18^F]**27**, [^18^F]**28** from the borylated precursors in excellent RCC, with and more importantly even without isolation of the boronate. This validates the method as a convenient way to access boronic acid esters of advanced drug intermediates and their effective radiofluorination. Optimisation of the labelling and purification lead to Cu-levels far below the limits for administration to man together with high yields, molar activity and radiochemical purity within the specifications for PET radiotracers.

## 4 Materials and Methods

### 4.1 General

Consumables, solvents, reagents and starting materials used in the experiments described herein were procured from Sigma-Aldrich (Sigma-Aldrich AS, Norway) or Fluorochem (Fluorochem Ltd., United Kingdom), in high quality unless specified otherwise. Intermediates and references were either obtained commercially or produced via standard methods from commercially available starting materials. The identity of intermediates and references was confirmed via comparison to literature reports when available. TLC was conducted on Silica gel 60 F_254_ coated aluminium TLC plates (Merck KGaA, Darmstadt, Germany), and developed using mixtures of ethyl acetate:hexanes (v:v) unless otherwise stated. Compounds on TLC plates were visualized under UV light (254 or 356 nm) and by staining with iodine or potassium permanganate solution. Nuclear magnetic resonance spectra were recorded on a Bruker AVII 400 NMR instrument (Bruker ASX Nordic AB). Chemical shifts (δ) for ^1^H (400 MHz), ^13^C (100 MHz) and ^19^F (376 MHz) resonances are reported in parts per million (ppm), relative to the solvent signal (CDCl_3_ δ = 7.223 ppm), downfield from a theoretical tetramethylsilane signal (TMS, δ = 0 ppm). Mass spectrometry was conducted on a Q-Tof-2 mass analyser (Micromass, Q-Tof-2TM) using ESI ion source in positive mode. Solid phase extraction (SPE) cartridges were purchased from VWR (VWR International, Darmstadt, Germany) and Sigma-Aldrich (Sigma-Aldrich AS, Norway). HPLC analysis of compound purity and quality control was conducted on a Hewlett-Packard 1100 HPLC system (Matriks AS, Oslo, Norway) consisting of a quaternary pump, variable wavelength diode array detector and a Raytest Gina star radioactivity detector (Raytest GmBH, Straubenhardt, Germany) using GABI-star software (Raytest) for instrument control, data acquisition and processing. Three HPLC methods were developed. For determination of the identity and purity of radiotracers, a Luna PFP column (Phenomenex; 5 μm, 100 Å, 250 mm × 4.6 mm) with an isocratic mixture of MeCN-water; 55:45 was used at a flow rate of 1 ml/min (System A) or an isocratic mixture of MeCN-water 40:60 at 1.0 ml/min flow rate (System B). Alternatively, a Kinetex EVO (Phenomenex; 5u, C18, 100Å, 250 × 4.6 mm) with an isocratic mixture of ammonium formate buffer (25 mM, pH = 9.2)-MeOH-MeCN; 60:30:10 at a 1.0 ml/min was used (System C). UV signals were detected at a wavelength of 254 nm. Radioactivity measurements during labelling experiments and radiotracer productions were performed using an Atomlab 300 dose calibrator (Biodex Medical Systems).

### 4.2 General Procedure for NMR Experiments

Aromatic amine (72 μmol), diboronate (1.2-4 equivalents) and catalyst (2.5–20 mol%) were weighed into 3 ml glass vial. A magnetic stir-bar was placed in the vial and the reagents were dissolved in the appropriate solvent (0.2 M relative to aromatic amine), followed by degassing of the mixture by passing inert gas (N_2_ or Ar) through the solution for 2–3 min. Alkyl nitrite (1.5 equivalents, 108 μmol) was then added to the solution in one portion, the vial was capped and placed on a hotplate set to the appropriate temperature with stirring. After the appropriate time, the reaction was quenched by cooling on an ice bath for 2 min. A sample from the reaction mixture (100 μL) was transferred directly into an NMR tube, diluted with CDCl_3_ (400 μL) and measured in the NMR spectrometer.

### 4.3 General Procedures for Borono-Deaminations

#### 4.3.1 General Procedure A

Aromatic amine (0.43 mmol), 4,4,4′,4′,5,5,5′,5′-octamethyl-2,2′-bi-1,3,2-dioxaborolane (B_2_pin_2_) (1.72 mmol, 435 mg) or 5,5,5′,5′-tetramethyl-2,2′-bi-1,3,2-dioxaborinane (B_2_neop_2_) (1.72 mmol, 388 mg) and tris(pentafluorophenyl)borane [B(C_6_F_5_)_3_] (0.01 mmol, 5.5 mg, 2.5 mol%) were weighed into a 5 ml glass vial. A magnetic stir-bar was placed in the vial, the reagents were dissolved in MeCN (2.15 ml, 0.2 M), and the mixture was degassed by passing inert gas (N_2_ or Ar were found to be appropriate) through the solution for 2–3 min. AmylONO (0.65 mmol, 86 μL) was added to the mixture all at once, the vial was capped and placed on the hotplate with stirring at 40°C for 15 min. The reaction was quenched by cooling on ice for 2 min, the solvent was removed under reduced pressure and the crude product purified by column chromatography.

#### 4.3.2 General Procedure B

The reagents were prepared in the same way as in general procedure A. The degassed reaction mixture was stirred at 80°C for 15 min. The reaction was quenched by cooling on ice for 2 min, the solvent was removed under reduced pressure and the crude purified by column chromatography.

#### 4.3.3 General Procedure C

Into a 5 ml glass vial was weighed in the aromatic amine (0.43 mmol) and then suspended in MeCN-AcOH (2.15 ml (0.2 M):123 μL (2.15 mmol)) and the mixture was stirred until all solids dissolved. In a separate vial B_2_pin_2_ (1.72 mmol, 435 mg), B_2_neop_2_ (1.72 mmol, 388 mg) and B(C_6_F_5_)_3_ (0.01 mmol, 5.5 mg, 2.5 mol%) were weighed in and dissolved in the previous mixture. The mixture was degassed by passing inert gas (N_2_ or Ar) through the solution for 2–3 min. AmylONO (0.65 mmol, 86 μL) was added to the mixture all at once, the vial was capped and placed on the hotplate with stirring at 40°C for 15 min. The reaction was quenched by cooling on ice for 2 min, the solvent was removed under reduced pressure and the crude product purified by column chromatography.

### 4.4 Synthesis of Boronic Acid Esters and Fluoride Reference Compounds

(S)-3-ethyl-3-[4-(4,4,5,5-tetramethyl-1,3,2-dioxaborolan-2-yl)phenyl]piperidine-2,6-dione (19a). The compound was synthesized using general procedure B with B_2_pin_2_. Purification by flash column chromatography on silica gel (80:18:2, hexanes:ethyl acetate:acetic acid) afforded the target compound in 78% yield (115 mg, 0.33 mmol) as white solids. Analytical data are in accordance with those previously reported (Firth et al., 2021). ^1^H NMR (400 MHz, Chloroform-*d*) δ 8.12 (s, 1H), 7.83 (d, *J* = 8.2 Hz, 2H), 7.30 (d, *J* = 8.3 Hz, 2H), 2.65 – 2.57 (m, 1H), 2.48 – 2.34 (m, 2H), 2.32 – 2.19 (m, 1H), 2.15 – 2.03 (m, 1H), 1.94 (dq, *J* = 14.6, 7.4 Hz, 1H), 1.36 (s, 12H), 0.89 (t, *J* = 7.4 Hz, 3H). ^13^C NMR (100 MHz, Chloroform-*d*) δ 174.91, 172.07, 141.87, 135.47, 125.51, 83.97, 51.42, 32.77, 29.31, 26.93, 24.86, 9.08. HR-ESIMS: m/z 366.1848 [M + Na]^+^ (C_19_H_26_BNNaO_4_, calculated 366.1853).

(*S*)-3-ethyl-3-[4-(5,5-dimethyl-1,3,2-dioxaborinan-2-yl)phenyl]piperidine-2,6-dione (19b). The compound was synthesized using general procedure B with B_2_neop_2_. Purification by flash column chromatography on silica gel (80:18:2, hexanes:ethyl acetate:acetic acid) afforded the target compound in 59% yield (43 mg, 0.14 mmol) as white solids. Analytical data are in accordance with those previously reported (Firth et al., 2021). ^1^H NMR (400 MHz, Chloroform-*d*) δ 7.86 (s, 1H), 7.79 (d, *J* = 8.3 Hz, 2H), 7.25 (d, *J* = 8.3 Hz, 2H), 3.76 (s, 4H), 2.58 (m, 1H), 2.47 – 2.30 (m, 2H), 2.27 – 2.15 (m, 1H), 2.06 (m, 1H), 1.93 (m, 1H), 1.27 (d, *J* = 12.2 Hz, 2H), 1.02 (s, 6H), 0.87 (t, *J* = 7.4 Hz, 3H). ^13^C NMR (100 MHz, Chloroform-*d*) δ 175.22, 172.41, 141.29, 134.67, 125.49, 72.46, 51.49, 32.90, 32.03, 29.84, 29.46, 27.26, 22.02. HR-ESIMS: m/z 352.1691 [M + Na]^+^ (C_18_H_24_BNNaO_4_, calculated 352.1696).

N-(5-methylisoxazol-3-yl)-4-(4,4,5,5-tetramethyl-1,3,2-dioxaborolan-2-yl)benzenesulfonamide (20a). The compound was synthesized using general procedure A with B_2_pin_2_. Purification by flash column chromatography on silica gel (80:18:2, hexanes:ethyl acetate:acetic acid) afforded the target compound in 86% yield (134 mg, 0.37 mmol) as beige solids. ^1^H NMR (400 MHz, Chloroform-*d*) δ 9.06 (s, 1H), 7.92 (d, *J* = 8.2 Hz, 2H), 7.84 (d, *J* = 8.2 Hz, 2H), 6.25 (s, 1H), 2.37 (s, 3H), 1.36 (s, 12H). ^13^C NMR (100 MHz, Chloroform-*d*) δ 171.02, 157.56, 141.06, 135.50, 125.99, 95.45, 84.51, 24.87, 12.72. HR-ESIMS: m/z 387.1156 [M + Na]^+^ (C_16_H_21_BN_2_NaO_5_S, 387.1162 calculated).

N-(5-methylisoxazol-3-yl)-4-(5,5-dimethyl-1,3,2-dioxaborinan-2-yl)benzenesulfonamide (20b). The compound was synthesized using general procedure A with B2neop2. Purification by flash column chromatography on silica gel (80:18:2, hexanes:ethyl acetate:acetic acid) afforded the target compound as a yellow solid in 78% yield (107 mg, 0.37 mmol) as beige solids. ^1^H NMR (400 MHz, Chloroform-*d*) δ 7.88 (d, J = 8.2 Hz, 2H), 7.79 (d, J = 8.2 Hz, 2H), 6.22 (s, 1H), 3.76 (s, 4H), 2.34 (s, 3H), 1.01 (s, 6H). ^13^C NMR (100 MHz, Chloroform-*d*) δ 170.93, 157.59, 140.49, 134.62, 125.87, 95.44, 72.42, 31.90, 26.92, 21.85, 12.72. HR-ESIMS: m/z 373.1000 [M + Na]^+^ (C_15_H_19_BN_2_NaO_5_S, 373.1005 calculated).

4-[*N*-(5-methylisoxazol-3-yl)sulfamoyl]benzenediazonium tetrafluoroborate. 4-amino-*N*-(5-methylisoxazol-3-yl)benzenesulfonamide (200 mg, 0.78 mmol) was dissolved in ethanol (0.5 ml), and cooled to 0°C in an ice bath. To the cooled stirring mixture was added 50% HBF_4_ (0.2 ml, 1.6 mmol). After 15 min of stirring *tert*-butyl nitrite (140 μL, 1.17 mmol) was added dropwise, and the mixture was removed from the ice bath. After stirring for 30 min a thick paste had formed, which was suspended in Et_2_O (2 ml) and stirred for 5 min. The suspension was filtered and the filter cake was washed with Et_2_O (10 ml) and air dried. The diazonium tetrafluoroborate salt was collected and placed in high vacuum for 2 h. The white fluffy crystals characteristic of diazonium tetrafluoroborates were obtained in 89% yield (245 mg, 0.69 mmol), which were used in the next step without purification or characterization (Bayarmangai, et al., 2015).

4-Fluoro-*N*-(5-methylisoxazol-3-yl)benzenesulfonamide (26). The reaction was done as described in the literature (Shinhama et al., 1993). 4- (*N*-(5-methylisoxazol-3-yl)sulfamoyl)benzenediazonium tetrafluoroborate (100 mg, 0.28 mmol) was placed in an oven dried 3 ml vial, and flushed with nitrogen gas. Boron trifluoride diethyl etherate (0.5 ml) was added to the vial under nitrogen. The mixture was heated via a heating block to 125°C, and was stirred for an hour. Ice cold water was added to the mixture and the organics were extracted with EtOAc (10 ml x2), washed with saturated sodium bicarbonate solution (5 ml x2) and water (5 ml). The organic phase was dried over Na_2_SO_4_, filtered and evaporated. The brown crude was purified by flash column chromatography (50:50 hexanes:ethyl acetate) to afford the target compound as colorless crystals in 76% yield (55 mg, 0.21 mmol). ^1^H NMR (400 MHz, Chloroform-*d*) δ 9.57 (s, 1H), 7.87 (ddd, *J* = 10.0, 5.0, 2.5 Hz, 2H), 7.15 (t, *J* = 8.6 Hz, 2H), 6.26 (s, 1H), 2.40 (s, 3H). ^13^C NMR (100 MHz, Chloroform-*d*) δ 171.27, 166.75, 164.21, 157.69, 135.13 (d, *J* = 3.3 Hz), 129.87 (d, *J* = 9.5 Hz), 116.57 (d, *J* = 22.8 Hz), 95.53, 12.75.^19^F NMR (376 MHz, Chloroform-*d*) δ -103.58 – -103.67 (m). HR-ESIMS: m/z 279.0210 [M + Na]^+^ (C_10_H_9_FN_2_NaO_3_S, calculated 279.0216).

methyl (*S*)-3-(4-aminophenyl)-2-[(tert-butoxycarbonyl)amino]propanoate. The compound was synthesized according to literature procedures (Juodaityte and Sewald, 2004). Purification by flash column chromatography (70:30, hexanes:ethyl acetate) afforded the target compound in 96% yield (435 mg, 1.47 mmol) as a slightly red sticky oil. Analytical data are in accordance with those previously reported (Ross et al., 2010). ^1^H NMR (400 MHz, Chloroform-*d*) δ 6.87 (d, *J* = 8.3 Hz, 2H), 6.58 (d, *J* = 8.4 Hz, 2H), 5.07 (d, *J* = 8.0 Hz, 1H), 4.48 (q, *J* = 6.0 Hz, 1H), 3.69 (bs, 2H), 3.68 (s, 3H), 2.94 (h, *J* = 7.3, 6.4 Hz, 2H), 1.41 (s, 9H).

methyl (*S*)2-[(tert-butoxycarbonyl)amino]-3-[4-(4,4,5,5-tetramethyl-1,3,2-dioxaborolan-2-yl)phenyl]propanoate (21a). The compound was synthesized using general procedure B with B_2_pin_2_. Purification by flash column chromatography on silica gel (85:13:2, hexanes:ethyl acetate:acetic acid) afforded the target compound in 69% yield (120 mg, 0.29 mmol) as a sticky colorless oil. Analytical data was in accordance with those previously reported (Ross et al., 2010). ^1^H NMR (400 MHz, Chloroform-*d*) δ 7.76 (d, *J* = 7.9 Hz, 2H), 7.15 (d, *J* = 7.6 Hz, 2H), 4.97 (d, *J* = 7.7 Hz, 1H), 4.60 (q, *J* = 6.0 Hz, 1H), 3.72 (s, 3H), 3.18–3.07 (m, 2H), 1.44 (s, 9H), 1.36 (s, 12H). ^13^C NMR (100 MHz, Chloroform-*d*) δ 172.24, 155.06, 139.22, 135.02, 128.72, 83.80, 79.96, 54.35, 52.23, 38.43, 28.31, 24.89, 24.87. HR-ESIMS: m/z 428.2215 [M + Na]^+^ (C_21_H_32_BNNaO_6_, 428.2220 calculated).

methyl (*S*)2-[(tert-butoxycarbonyl)amino]-3-[4-(5,5-dimethyl-1,3,2-dioxaborinan-2-yl]propanoate (19b). The compound was synthesized using general procedure B with B_2_neop_2_. Purification by flash column chromatography on silica gel (85:13:2, hexanes:ethyl acetate:acetic acid) afforded the target compound in 28% yield (36 mg, 0.05 mmol) as a sticky yellow oil. ^1^H NMR (400 MHz, Chloroform-*d*) δ 7.72 (d, *J* = 8.0 Hz, 2H), 7.11 (d, *J* = 7.8 Hz, 2H), 4.93 (d, *J* = 8.3 Hz, 1H), 4.58 (q, *J* = 5.9 Hz, 1H), 3.76 (s, 4H), 3.69 (s, 3H), 3.08 (m, 2H), 1.43 (s, 9H), 1.02 (s, 6H). ^13^C NMR (100 MHz, Chloroform-*d*) δ 172.44, 155.22, 138.64, 134.24, 128.72, 72.45, 71.69, 54.49, 52.33, 38.51, 32.03, 28.44, 27.06, 22.05. HR-ESIMS: m/z 414.2059 [M + Na]^+^ (C_20_H_30_BNNaO_6_, 414.2064 calculated).

methyl 2-[(tert-butoxycarbonyl)amino]-3-(4-fluorophenyl)propanoate (27). The compound was synthesized from methyl (*S*)2-((tert-butoxycarbonyl)amino)-3-(4-(4,4,5,5-tetramethyl-1,3,2-dioxaborolan-2-yl)phenyl)propanoate following literature procedures in a 25 mg (0.06 mmol) scale (Furuya and Ritter, 2009). Purification by column chromatography (80:20, hexanes:ethyl acetate) afforded the target compound as a colorless oil in 33% yield (6 mg, 0.02 mmol). Analytical data were in accordance with those previously reported (Feng et al., 2014). ^1^H NMR (400 MHz, Chloroform-*d*) δ 7.11 (dd, *J* = 8.4, 5.8 Hz, 2H), 7.00 (t, *J* = 8.6 Hz, 2H), 5.06 – 4.92 (m, 1H), 4.70 – 4.51 (m, 1H), 3.74 (s, 3H), 3.21 – 2.98 (m, 2H), 1.44 (s, 9H).

2-(diethylamino)ethyl 4-(4,4,5,5-tetramethyl-1,3,2-dioxaborolan-2-yl)benzoate (20a). The compound was synthesized using general procedure C with B_2_pin_2_. Purification by flash column chromatography on silica gel (95:5, chloroform:methanol) afforded the target compound in 54% yield (80 mg, 0.23 mmol) as a yellow oil. ^1^H NMR (400 MHz, Chloroform-*d*) δ 8.03 (d, *J* = 8.2 Hz, 2H), 7.89 (d, *J* = 8.2 Hz, 2H), 4.43 (t, *J* = 6.2 Hz, 2H), 2.91 (t, *J* = 6.2 Hz, 2H), 2.68 (q, *J* = 7.1 Hz, 4H), 1.38 (s, 12H), 1.10 (t, *J* = 7.1 Hz, 6H). ^13^C NMR (100 MHz, Chloroform-*d*) δ 166.61, 134.67, 132.38, 128.62, 84.19, 63.25, 50.94, 47.85, 24.89, 11.88. HR-ESIMS: m/z 348.2341 [M + H]^+^ (C_19_H_31_BNO_4_, 348.2346 calculated).

2-(diethylamino)ethyl 4-(5,5-dimethyl-1,3,2-dioxaborinan-2-yl)benzoate (20b). The compound was synthesized using general procedure C with B_2_neop_2_. Purification by flash column chromatography on silica gel (95:5, chloroform:methanol) afforded the target compound in 15% yield (21 mg, 0.063 mmol) as a yellow oil. ^1^H NMR (400 MHz, Chloroform-*d*) δ 8.05 (d, *J* = 8.2 Hz, 1H), 7.45 (d, *J* = 8.05 Hz, 1H), 4.47 (t, *J* = 6.1 Hz, 2H), 3.51 (s, 4H), 2.96 (t, *J* = 6.1 Hz, 2H), 2.72 (q, *J* = 7.2 Hz, 4H), 1.13 (t, *J* = 7.2 Hz, 6H), 0.92 (s, 6H). ^13^C NMR (100 MHz, Chloroform-*d*) δ 166.58, 133.04, 130.08, 129.61, 128.40, 71.63, 62.61, 50.60, 47.47, 36.46, 21.34, 11.34. HR-ESIMS: m/z 334.2185 [M + H]^+^ (C_18_H_29_BNO_4_, 334.2190 calculated).

5-(4-(((4-methoxybenzyl) (methyl)amino)methyl)phen yl)-8-(4,4,5,5-tetramethyl-1,3,2-dioxaborolan-2-yl)-2,3,4,6-tetrahydro-1H-azepino[5,4,3-cd]indol-1-one (21b)**.** The compound was synthesized using general procedure C, with B_2_pin_2_. Purification by flash column chromatography on silica gel (92.5:7.5, chloroform:methanol) afforded the target compound in 30% yield (165 mg, 0.3 mmol) as an orange solid. ^1^H NMR (400 MHz, DMF-*d*_7_) δ 11.81 (s, 1H), 8.47 (s, 1H), 8.21 (d, *J* = 3.0 Hz, 2H), 7.93 (d, *J* = 8.0 Hz, 2H), 7.76 (d, *J* = 8.0 Hz, 2H), 7.54 (d, *J* = 8.5 Hz, 2H), 7.14 (d, *J* = 8.6 Hz, 2H), 3.99 (s, 3H), 3.78 (bs, 4H), 3.71 (bs, 2H), 3.35 (bs, 2H), 2.34 (s, 3H), 1.56 (s, 12H). ^13^C NMR (100 MHz, DMF-*d*_7_) δ 170.53, 159.08, 136.87, 131.53, 131.07, 130.20, 129.45, 129.37, 129.13, 128.97, 128.42, 128.29, 127.76, 124.73, 121.00, 113.84, 112.70, 83.83, 61.13, 55.07, 42.60, 41.67 24.71.HR-ESIMS: m/z 552.3028 [M + H]^+^ (C_33_H_39_BN_3_O_4_, 552.3034 calculated).

5-(4-(((4-methoxybenzyl) (methyl)amino)methyl)phen yl)-8-(5,5-dimethyl-1,3,2-dioxaborinan-2-yl)-2,3,4,6-tetrahydro-1H-azepino[5,4,3-cd]indol-1-one (21b). The compound was synthesized using general procedure C with B_2_neop_2_. Purification by flash column chromatography on silica gel (92.5:7.5, chloroform:methanol) afforded the target compound in 15% yield (27 mg, 0.05 mmol) as an orange solid. ^1^H NMR (400 MHz, DMF-*d*_7_) δ 11.35 (s, 1H), 7.91 (d, *J* = 8.8 Hz, 1H), 7.74 (m, 1H), 7.70 (d, *J* = 8.0 Hz, 2H), 7.64 (d, *J* = 8.6Hz, 1H), 7.55 (d, *J* = 8.0 Hz, 2H), 7.36 (d, *J* = 8.5 Hz, 2H), 7.27 (t, *J* = 7.8 Hz, 1H), 6.94 (d, *J* = 8.5 Hz, 2H), 3.86 (s, 3H), 3.60 (m, 2H), 3.56 (s, 2H), 3.43 (s, 4H), 3.25 (m, 2H), 2.25 (s, 3H), 0.92 (s, 6H). HR-ESIMS: m/z 538.2877 [M + H]^+^ (C_32_H_37_BN_3_O_4_, 538.2877 calculated).

### 4.5 General Procedures for Radiochemistry

#### 4.5.1 Preparation of [^18^F]fluoride

A SupelTM-Select SAX SPE (30 mg) cartridge was preconditioned for [^18^F]fluoride extraction by slowly passing deionised water (10 ml) through the cartridge, followed by removal of excess liquid by air (30 ml) from a syringe. Elution solution was prepared by completely dissolving tetraethyl ammonium bicarbonate (36 μmol) in a 9:1 mixture of acetonitrile (MeCN):H_2_O and in concentrations of. An aliquot of the [^18^F]fluoride in [^18^O]H_2_O from the cyclotron, was withdrawn into the preconditioned cartridge, and air was passed through (10 ml) to remove excess target water. The [^18^F]fluoride was eluted from the cartridge by passage of elution solution (1 ml) through it, and the eluent was collected into a V-vial. The solvent was then evaporated on a hot plate at 85°C under an argon stream. Residual water was removed from the [^18^F]fluoride by additional 3 cycles of addition/evaporation of MeCN (1 ml). The vial was removed from the hot plate, quickly sealed with a screwcap and cooled for about 30 s on an ice bath.

#### 4.5.2 Radiofluorination: Screening Reaction

Arylboronic acid ester (15 μmol), was dissolved in anhydrous DMA (0.25 ml). Copper (II)triflate (5 mg, 15 μmol) was suspended in DMA (0.25 ml), followed by addition of anhydrous pyridine (50 μL, 60 μmol). The mixture was prepared no more than 10 min before reaction start. The tetrakis (pyridine)copper (II) triflate solution (0.25 ml) is added to the dry and cooled [^18^F]fluoride, followed by addition of radiolabeling precursor solution (0.25 ml). The reaction mixture was heated to 120°C, and left to react for 20 min. After the time has elapsed the mixture is cooled for 2.5 min. Water (0.5 ml) is added and an aliquot of the mixture (40 μL) is transferred into an Eppendorf tube, diluted with MeCN (0.2 ml) and injected (10 µL) into an analytical radioHPLC for analysis.

## Data Availability

The original contributions presented in the study are included in the article/Supplementary Material, further inquiries can be directed to the corresponding author.
