# Supplementary material for: Mild, Organo-Catalysed Borono-Deamination as a Key to Late-Stage Pharmaceutical Precursors and 18F-Labelled Radiotracers
Source: Front Chem. 2022 Apr 26;10:884478. doi: 10.3389/fchem.2022.884478 (PMC9089349; doi:10.3389/fchem.2022.884478)
Supplement: Supplementary file 1 [file DataSheet1.pdf]

Supplementary Material

Contents

1 Supplementary Data 2

Experimental Procedures 2

General 2

Recovery of B_2pin_2 2

Total synthesis of 5-(4-(((4-methoxybenzyl)(methyl)amino)methyl)phenyl)-8-(4,4,5,5-tetramethyl-1,3,2-dioxaborolan-2-yl)-2,3,4,6-tetrahydro-1H-azepino[5,4,3-cd]indol-1-one (23a and 23b), for its use as a precursor for radiofluorinations. 3

Synthetic procedures. 3

^{1}H-NMR Experiments for the screening of reaction conditions 6

NMR yield calculation 6

Radiochemistry 7

ICP-MS analysis 9

2 Supplementary Figures and Tables 10

Analytical data 15

NMR Spectra 22

1 Supplementary Data

Experimental Procedures

General

Solvents and reagents used in the experiments described herein were procured from Sigma-Aldrich (Sigma-Aldrich AS, Norway) or Fluorochem (Fluorochem Ltd., UK), in highest available quality unless specified otherwise. Starting materials were obtained from commercial suppliers. Intermediates and references were either obtained commercially or produced via standard methods from commercially available starting materials. The identity of intermediates and references was confirmed via comparison to literature reports. Solid phase extraction (SPE) cartridges were purchased from VWR (VWR International, Darmstadt, Germany) and Sigma-Aldrich (Sigma-Aldrich AS, Norway). TLC was conducted on Silica gel 60 F254 coated aluminium TLC plates (Merck KGaA, Darmstadt, Germany), and developed using mixtures of ethyl acetate:hexanes (v:v) unless otherwise stated. Compounds on TLC plates were visualized under UV light (254 or 356 nm) and by staining with iodine or potassium permanganate. Silica gel 60Å (40-63 µm, 230-400 mesh) (Merck-Millipore) was used as the solid phase for flash column chromatography, unless otherwise stated. Nuclear magnetic resonance spectra were recorded on a Bruker AVII 400 NMR instrument (Bruker ASX Nordic AB). Chemical shifts (δ) for ^{1}H (400 MHz), ^{13}C (100 MHz) and ^{19}F (376 MHz) resonances are reported in parts per million (ppm), relative to the solvent signal ($CDCl_3$ δ = 7.223 ppm), downfield from a theoretical tetramethylsilane signal (TMS, δ = 0 ppm). Mass spectrometry was conducted on a Q-Tof-2 mass analyser (Micromass, Q-Tof-2TM) using ESI ion source in positive mode. HPLC analysis of compound purity and quality control was conducted on a Hewlett-Packard 1100 HPLC system (Matriks AS, Oslo, Norway) consisting of a quaternary pump, variable wavelength diode array detector and a Raytest Gina star radioactivity detector (Raytest GmBH, Straubenhardt, Germany) using GABI-star software (Raytest) for instrument control, data acquisition and processing. Three HPLC methods were developed. For determination of the identity and purity of radiotracers, a Luna PFP column (Phenomenex; 5 µm, 100 Å, 250 mm × 4.6 mm) with an isocratic mixture of MeCN-water; 55:45 was used at a flow rate of 1 mL/min (System A) or an isocratic mixture of MeCN-water 40:60 at 1.0 mL/min flow rate (System B). Alternatively, a Kinetex EVO (Phenomenex; 5u, C18, 100Å, 250 x 4.6 mm) with an isocratic mixture of ammonium formate buffer (25 mM, pH=9.2)-MeOH-MeCN; 60:30:10 at a 1.0 mL/min was used (System C). UV signals were detected at a wavelength of 254 nm. Radioactivity measurements during labelling experiments and radiotracer productions were performed using an Atomlab 300 dose calibrator (Biodex Medical Systems).

Recovery of B_2pin_2

Recovery of B_2pin_2 was achieved by dilution of the reaction mixture with 5 volumes of Et_2O followed by cooling to 4 °C over night. The reagent was recovered as colourless crystals after decanting or filtration of the mother liquor. Following concentration, the product is obtained via purification on silica gel. Residual B_2pin_2 was recovered in the fractions eluting before the desired products. Recovery was calculated to be around 46% (200 mg, 0.79 mmol) of the amount employed in the reaction. ^{1}H NMR (400 MHz, Chloroform-*d*) δ 1.28 (s, 1H). ^{13}C NMR (100 MHz, Chloroform-*d*) δ 83.51, 25.03.

The recovered B_2pin_2 had higher purity (by ^{1}H-NMR spectroscopy) than the material obtained from commercial sources (Figure S1).

Total synthesis of 5-(4-(((4-methoxybenzyl)(methyl)amino)methyl)phenyl)-8-(4,4,5,5-tetramethyl-1,3,2-dioxaborolan-2-yl)-2,3,4,6-tetrahydro-1H-azepino[5,4,3-cd]indol-1-one (23a and 23b), for its use as a precursor for radiofluorinations.

In efforts to obtain a radiotracer for poly-ADP ribose polymerase (PARP) from the PARP-inhibitor rucaparib (1), we designed a total synthesis to introduce a leaving group to be substituted for [^{18}F]fluoride ion (Figure S2). We then devised the following synthetic route (Figure S3), to the boronic acid ester precursor synthesized by this method.

Synthetic procedures.

methyl 6-((tert-butoxycarbonyl)amino)-1*H*-indole-4-carboxylate (S1). Methyl-6-amino-1*H*-indole-4-carboxylate (3.5 g, 18.4 mmol) was dissolved in THF (50 mL). To the stirring solution was added di-*tert*-Butyl dicarbonate (4.42 g, 20.2 mmol) in one portion at room temperature. The solution was then heated to 50 °C, and gas evolution was visible in the form of bubbles after 5-10 min. After 2 h at this temperature gas evolution had ended and TLC showed consumption of all starting materials. The solvent was evaporated under reduced pressure, and the beige residue was recrystallized from EtOH/H_2O to afford the product as small colourless/pink crystals in 96% yield (5.12 g, 17.6 mmol). ^{1}H NMR (400 MHz, Chloroform-*d*) δ 8.34 (bs, 1H), 8.13 (bs, 1H), 7.59 (d, J = 1.9 Hz, 1H), 7.32 – 7.29 (m, 1H), 7.11 – 7.09 (m, 1H), 6.65 (bs, 1H), 4.00 (s, 3H), 1.56 (s, 9H). ^{13}C NMR (100 MHz, Chloroform-*d*) δ 167.63, 153.14, 137.21, 132.73, 125.93, 123.62, 121.49, 106.25, 103.68, 80.60, 51.89, 28.40. HR-ESIMS: *m/z* 313.1162 $[M+Na]^+$ ($C_{15}H_{18}N_2O_4Na$, calculated 313.1164).

methyl 6-((tert-butoxycarbonyl)amino)-3-formyl-1*H*-indole-4-carboxylate (S2). To a round bottomed flask which had been air dried under argon, was added DMF (5 mL) and cooled on an ice bath. $POCl_3$ (3.2 mL, 34.4 mmol) was then added to the stirring solvent dropwise, being careful to maintain the temperature below 5 °C. Methyl 6-((*tert*-butoxycarbonyl)amino)-1*H*-indole-4-carboxylate (5 g, 17.2 mmol) was dissolved in anhydrous DMF (10 mL), and added to the stirring DMF/$POCl_3$ mixture over the course of 15 min. The solution was stirred on the ice bath for an additional 45 min, and then allowed to warm up to room temperature and stirred for an additional h. The solution was transferred into an Erlenmeyer flask, with crushed ice (400 mL) and stirred vigorously. A yellow paste immediately appeared. This suspension then neutralized by dropwise addition of NaOH (3 M) with vigorous stirring, until the pH was neutral (pH paper indicator) and then basified with saturated K_2CO_3, while maintaining the solution cooled. The solution was allowed to decant in the refrigerator overnight. The precipitate was filtered off, washed with water, and dried under high vacuum to leave a beige solid which was the product in 92% yield (5.04 g, 15.8 mmol), and which was pure enough to be used in the next step without further purification. ^{1}H NMR (400 MHz, DMSO-d_6) δ 12.27 (s, 1H), 10.10 (s, 1H), 9.59 (s, 1H), 8.24 (s, 1H), 7.97 (s, 1H), 7.67 (d, J = 2.0 Hz, 1H), 3.85 (s, 3H), 1.50 (s, 9H). ^{13}C NMR (100 MHz, DMSO-d_6) δ 186.29, 168.95, 153.40, 138.91, 137.03, 135.47, 125.13, 118.63, 116.98, 115.27, 105.25, 79.75, 52.44, 28.61. HR-ESIMS: *m/z* 341.1111 $[M+Na]^+$ ($C_{16}H_{18}N_2O_5Na$, calculated 341.1113).

methyl (*E*)-6-((*tert*-butoxycarbonyl)amino)-3-(2-nitrovinyl)-1*H*-indole-4-carboxylate (S3). To a suspension of methyl-6-((*tert*-butoxycarbonyl)amino)-3-formyl-1*H*-indole-4-carboxylate (5 g, 15.7 mmol) in nitromethane (100 mL) was added ammonium acetate (363 mg, 4.71 mmol). The reaction mixture was heated to 60 °C and stirred for 4-8 h. After TLC showed that the starting material had

been consumed, volatiles were evaporated under reduced pressure. The yellow crude product was redissolved in EtOAc (400 mL) and washed with water (50 mL x3) and brine (100 mL). The organic phase was dried over Na_2SO_4 and evaporated to dryness to leave an orange solid which was purified by recrystallization from MeOH/H_2O to leave a yellow solid which was the product in 94% yield (5.4 g, 14.7 mmol). ^{1}H NMR (400 MHz, DMSO-*d*$_6$) δ 12.44 (s, 1H), 9.62 (s, 1H), 9.13 (d, *J* = 13.4 Hz, 1H), 8.48 (s, 1H), 8.01 (s, 1H), 7.98 (d, *J* = 13.3 Hz, 1H), 7.95 (d, *J* = 2.0 Hz, 1H), 3.94 (s, 3H), 1.50 (s, 9H). ^{13}C NMR (100 MHz, DMSO-*d*$_6$) δ 167.76, 153.41, 139.37, 137.61, 135.07, 132.52, 132.21, 122.99, 119.93, 117.43, 107.81, 107.48, 106.73, 79.77, 52.76, 28.61. HR-ESIMS: *m/z* 384.1166 $[M+Na]^+$ ($C_{17}H_{19}N_3NaO_6$, calculated 384.1171).

methyl 6-((tert-butoxycarbonyl)amino)-3-(2-nitroethyl)-1*H*-indole-4-carboxylate (S4). Methyl (*E*)-6-((*tert*-butoxycarbonyl)amino)-3-(2-nitrovinyl)-1*H*-indole-4-carboxylate (2 g, 5.5 mmol) was dissolved in a 5:1 mixture of tetrahydrofuran:methanol (300 mL). To this mixture was added $NaBH_4$ (416 mg, 11 mmol) portion wise over the course of 0.5 h. Another 0.5 h after the addition of the reducing agent, TLC indicated consumption of all starting materials. Water (200 mL) was added, and the mixture was cooled over an ice bath before the dropwise addition of 1M HCl until the pH was acidic (pH 2). This made the solution turn from an orange to a yellow color with precipitation of solids. The organic phase was extracted with ethyl acetate (150 mL x 4), the combined organic layers washed with water (100 mL x 2) and brine (100 mL) and dried over Na_2SO_4. After filtration and evaporation under reduced pressure, the crude product was purified by flash column chromatography (75:25 hexanes:ethyl acetate) affording the target compound as light yellow solids in 57% yield (1.14 g, 3.13 mmol). ^{1}H NMR (400 MHz, DMSO-*d*$_6$) δ 11.23 (d, *J* = 2.5 Hz, 1H), 9.42 (s, 1H), 7.88 – 7.83 (m, 1H), 7.74 (d, *J* = 2.0 Hz, 1H), 7.25 (d, *J* = 2.4 Hz, 1H), 4.72 (t, *J* = 7.0 Hz, 2H), 3.88 (s, 3H), 3.45 (t, *J* = 6.9 Hz, 2H), 1.50 (s, 9H). ^{13}C NMR (100 MHz, DMSO-*d*$_6$) δ 168.29, 153.48, 138.73, 133.58, 127.19, 122.53, 119.91, 115.16, 109.43, 106.02, 79.43, 77.39, 52.44, 28.65, 25.99. HR-ESIMS: *m/z* 386.1323 $[M+Na]^+$ ($C_{17}H_{21}N_3NaO_6$, calculated 386.1328).

***tert*-butyl (1-oxo-2,3,4,6-tetrahydro-1*H*-azepino[5,4,3-cd]indol-8-yl)carbamate (S5).** Methyl 6-((*tert*-butoxycarbonyl)amino)-3-(2-nitroethyl)-1*H*-indole-4-carboxylate (4.27 g, 11.7 mmol) was suspended in methanol (250 mL). The solvent was degassed and the atmosphere saturated with nitrogen gas (N_2). Raney-Ni (5 g of the aqueous slurry), was washed with distilled water (10 mL x3), methanol (15 mL x3) and transferred to the suspension of the starting material. The atmosphere of the reaction vessel was saturated with H_2 (g) and the reaction was stirred for 8 h. After this time, the organic solids had gone into solution and TLC showed complete consumption of starting materials. The mixture was filtered through celite, and the filter cake washed with warm methanol (50 mL x 3). The clear orange filtrate was then heated to refluxed overnight. The volatiles were evaporated to leave a brown crude product which was loaded onto a silica plug which was eluted with 95:5 chloroform:methanol. Evaporation of the eluate afforded light brown solids which were the product in 52% yield (1.8 g, 6.1 mmol). ^{1}H NMR (400 MHz, DMSO-*d*$_6$) δ 10.92 (s, 1H), 9.31 (s, 1H), 7.97 (t, J = 5.7 Hz, 1H), 7.79 (s, 1H), 7.72 (s, 1H), 7.13 (d, J = 2.1 Hz, 1H), 3.43 – 3.35 (m, 2H), 2.96 – 2.80 (m, 2H), 1.49 (s, 9H). ^{13}C NMR (100 MHz, DMSO-*d*$_6$) δ 169.98, 153.56, 137.38, 134.09, 124.70, 122.60, 121.23, 115.02, 114.61, 105.04, 79.14, 42.74, 28.69, 28.64. HR-ESIMS: *m/z* 324.1319 $[M+Na]^+$ ($C_{16}H_{19}N_3NaO_3$, calculated 324.1324).

***tert*-butyl (5-bromo-1-oxo-2,3,4,6-tetrahydro-1*H*-azepino[5,4,3-*cd*]indol-8-yl)carbamate (S6).** *tert*-Butyl (5-bromo-1-oxo-2,3,4,6-tetrahydro-1*H*-azepino[5,4,3-*cd*]indol-8-yl)carbamate (5.4 g, 17.9 mmol) was dissolved in a 1:1 mixture of CH_2Cl_2:tetrahydrofuran (150 mL), and cooled to 0 °C on an

ice bath. Pyridinium tribromide (6.2 g, 19.5 mmol) was added to the stirring solution in portions over a period of 1 h. After the addition was complete the ice bath was removed, the mixture was allowed to warm up to room temperature and stirred for an additional hour. After this time TLC indicated consumption of all starting materials. The mixture was filtered through celite, the filtrate collected, and the solvent removed under reduced pressure. The crude product was dissolved in chloroform (300 mL), washed with water (50 mL x 3), and brine (100 mL). The organic phase was dried over Na_2SO_4, filtered and the solvent was removed under reduced pressure. The residue was purified by flash column chromatography (95:5, diethyl ether:methanol) which after evaporation afforded a brown solid which was the product in 81% (5.46, 14.3 mmol). ^{1}H NMR (400 MHz, DMSO-d_6) δ 11.73 (s, 1H), 9.40 (s, 1H), 8.05 (t, J = 5.7 Hz, 1H), 7.76 (s, 1H), 7.75 – 7.73 (m, 2H), 3.45 – 3.36 (m, 2H), 2.80 – 2.69 (m, 2H), 1.49 (s, 9H). ^{13}C NMR (100 MHz, DMSO-d_6) δ 169.45, 153.47, 137.21, 124.04, 114.18, 107.76, 79.34, 28.66, 28.35. HR-ESIMS: *m/z* 402.0424 $[M+Na]^+$ ($C_{16}H_{18}BrN_3NaO_3$, calculated 402.0429).

***tert*-butyl (5-(4-formylphenyl)-1-oxo-2,3,4,6-tetrahydro-1*H*-azepino[5,4,3-*cd*]indol-8-yl)carbamate (S7).** To a solution of *tert*-butyl (5-bromo-1-oxo-2,3,4,6-tetrahydro-1*H*-azepino[5,4,3-*cd*]indol-8-yl)carbamate (5.23 g, 13.8 mmol) in degassed dimethylacetamide (50 mL) was added 1,1'-Bis-(diphenylphosphino)ferrocene palladium(II)dichloride (256 mg, 0.34 mmol, 2.5 mol%) and the mixture was stirred for 1 h at room temperature. In another container, 4-formylphenyl boronic acid (2.48 g, 16.5 mmol) dissolved in degassed dimethyl acetamide (50 mL) was mixed with a solution of Na_2CO_3 (2.9 mg, 23 mmol) in water (10 mL), and the resulting mixture was heated to 90 °C for 2 h. The two separate solutions were mixed together and heated at 90 °C for 2 h. The mixture was cooled down to room temperature and stirred for an additional 2 h, during which a yellow solid precipitated. Ice cold water (100 mL) was added to the mixture which precipitated more solids, and the resulting suspension was filtered and washed with ice cold water. The yellow solids were resuspended in MeOH (30 mL), heated to 60 °C for an hour, after which the suspension was cooled to 0 °C. The solids were filtered off, washed with cold MeOH (20 mL) and dried under vacuum to leave a yellow solid which was the product in 79% yield (4.13 g, 10.9 mmol). ^{1}H NMR (400 MHz, DMSO-d_6) δ 11.57 (s, 1H), 10.05 (s, 1H), 9.47 (s, 1H), 8.12 (t, J = 5.7 Hz, 1H), 8.03 (d, J = 7.9 Hz, 2H), 7.90 (s, 1H), 7.84 (d, J = 8.0 Hz, 2H), 7.80 (s, 1H), 3.40 (bs, 2H), 3.21 – 3.01 (m, 2H), 1.51 (s, 9H). ^{13}C NMR (100 MHz, DMSO-d_6) δ 192.97, 169.66, 153.47, 138.21, 137.89, 135.45, 134.80, 132.75, 130.38, 128.25, 125.67, 122.22, 114.88, 79.38, 42.34, 29.70, 28.68. HR-ESIMS: *m/z* 428.1581 $[M+Na]^+$ ($C_{23}H_{23}N_3NaO_4$, calculated 428.1586).

***tert*-butyl (5-(4-(((4-methoxybenzyl)(methyl)amino)-methyl)phenyl)-1-oxo-2,3,4,6-tetrahydro-1*H*-azepino [5,4,3-*cd*]indol-8-yl) carbamate (S8).** To a solution of *tert*-butyl (5-(4-formylphenyl)-1-oxo-2,3,4,6-tetrahydro-1*H*-azepino[5,4,3-*cd*]indol-8-yl) carbamate (4.15 g, 10.8 mmol) dissolved in dimethylformamide (50 mL), was added $NaB(OAc)_3H$ (2.77 g, 13 mmol), followed by dropwise addition of 4'-methoxy-N-methylbenzylamine (1.8 mL, 11.9 mmol). The mixture was stirred overnight at room temperature. After this time all starting material had been consumed, and the mixture was diluted with ice cold water (200 mL), and the solution was carefully basified by addition of NaOH solution (1 M) to a pH value of around 10. The organics were extrated with EtOAc (100 mL x 4), washed with water (50 mL x 2), dried over Na_2SO_4 and filtered. The filtrate was evaporated and the crude product was purified by flash column chromatography (97.5:2.5 chloroform:methanol) to leave a yellow foamy solid which was the product in 42% (2.51 g, 4.62 mmol). ^{1}H NMR (400 MHz, DMSO-d_6) δ 11.33 (s, 1H), 9.39 (s, 1H), 8.05 (t, J = 5.8 Hz, 1H), 7.85 (s, 1H), 7.76 (d, J = 1.7 Hz, 1H), 7.58 (d, J = 8.2 Hz, 2H), 7.46 (d, J = 8.2 Hz, 2H), 7.28 (d, J = 8.6 Hz, 2H), 6.91 (d, J = 8.6 Hz, 2H), 3.74 (s, 3H), 3.52 (s, 2H), 3.46 (s, 2H), 3.39 – 3.36 (m, 3H), 3.07 – 2.97 (m, 2H), 2.09 (s,

3H), 1.51 (s, 10H). ^{13}C NMR (100 MHz, DMSO-d_6) δ 169.94, 158.72, 153.52, 138.85, 137.31, 134.46, 134.18, 131.29, 131.20, 130.27, 129.36, 127.93, 124.96, 122.51, 114.07, 112.02, 79.22, 60.97, 60.90, 55.46, 42.50, 42.07, 29.53, 28.69. HR-ESIMS: *m/z* 541.2810 $[M+H]^+$ ($C_{32}H_{37}N_4O_4$, calculated 541.2815).

8-amino-5-(4-(((4-methoxybenzyl)(methyl)amino)methyl)phenyl)-2,3,4,6-tetrahydro-1*H*-azepino[5,4,3-*cd*]indol-1-one (S9). *tert*-butyl (5-(4-(((4-methoxybenzyl)(methyl)amino)methyl)phenyl)-1-oxo-2,3,4,6-tetrahydro-1*H*-azepino[5,4,3-*cd*]indol-8-yl)carbamate (500 mg, 0.92 mmol) was suspended in CH_2Cl_2 (7.5 mL). To this suspension was added trifluoroacetic acid (2.5 mL). After 1 h TLC showed all starting materials had been consumed, and all solids had gone into solution. CH_2Cl_2 (100 mL) was added to the mixture and the organic phase was washed with a saturated solution of sodium bicarbonate (50 mL x 2), and with water (30 mL). The organic phases were dried over Na_2SO_4, filtered and evaporated. The crude product was subsequently purified by flash column chromatography (90:10 chloroform:methanol) to give a yellow solid which was the product in 91% yield (368 mg, 0.83 mmol). ^{1}H NMR (400 MHz, DMSO-d_6) δ 10.87 (s, 1H), 7.91 (t, J = 5.8 Hz, 1H), 7.53 (d, J = 8.2 Hz, 2H), 7.43 (d, J = 8.1 Hz, 2H), 7.27 (d, J = 8.6 Hz, 2H), 7.11 (d, J = 2.0 Hz, 1H), 6.91 (d, J = 8.6 Hz, 2H), 6.75 (d, J = 2.0 Hz, 1H), 5.00 (s, 2H), 3.74 (s, 3H), 3.51 (s, 2H), 3.45 (s, 2H), 3.35-3.30 (m, 2H), 3.00-2.95 (m, 2H), 2.09 (s, 3H). 13C NMR (100 MHz, DMSO-d_6) δ 170.35, 158.72, 144.63, 138.75, 131.74, 131.52, 131.31, 130.27, 129.28, 127.49, 125.29, 119.20, 114.06, 112.68, 112.12, 98.94, 61.01, 60.86, 55.46, 42.54, 42.06, 29.72. HR-ESIMS: *m/z* 441.2285 $[M+H]^+$ ($C_{27}H_{29}N_4O_2$, calculated 441.2291).

^{1}H-NMR Experiments for the screening of reaction conditions

Spectra of samples from the screening of reaction conditions (**General procedure for NMR experiments**) were taken, using 4-nitroaniline as a model compound. Peaks for aromatic product and starting materials were identified by adding an external standard of 4-nitrophenyl boronate or 4-nitroaniline to reaction samples. The chemical shifts of the different intermediates were accounted for (Table S1; Figures S4, S5, S6, S7 and S8), and were subsequently used to calculate the NMR yields. For the neopentyl derivatives, there was no change in the chemical shift for the aromatic signals for the products. (Table S1).

NMR yield calculation

Yield for individual reactions were calculated by integrating the proton signals originating from the products and any other intermediates present in the reaction mixture in the range between 13-6 ppm. The total integral from the product was then divided by the total integral of all the signals in the aromatic region and multiplied by 100 to give the yield (Equation 1).

$$Yield = \frac{Total\ integral\ from\ product}{Total\ integral\ from\ all\ aromatic\ signals} \cdot 100\ (Equation\ 1)$$

There is no need to account for the number of protons from which the signals originate, since the only source of aromatic protons in all reactions mixtures is the starting material.

Alternatively, the signal originating from the product (**IP**) either in the aliphatic region (5-0 ppm), and the sum of the integrals from all boron associated protons (**IBtot**) were normalized for the number of protons (N =12). The theoretical yield integral (**Iy_{Th}**) was found by dividing the normalized **IBtot** by the ratio between the amount of aniline to boron (monomer) in the mixture (Equation 2).

$$Iy_{Th} = \frac{IBtot}{\left(\frac{Eq.\ Aniline}{Eq.Boron}\right)} \quad (Equation\ 2)$$

IP was then divided by **Iy_{Th}** multiplied by 100 to obtain the yield (Equation 3).

$$yield = \left(\frac{IP}{Iy_{Th}}\right) \times 100\ (Equation\ 3)$$

Radiochemistry

Experimental procedures for the Cu(II) mediated radiofluorination of 23a and 23b

Due to the poor solubility of **23a** and **23b** in conventional solvents and the need for an additional deprotection step to remove the PMB protective group from the benzylic amine, standard fluorination was performed slightly different. This is summarized in scheme 2.

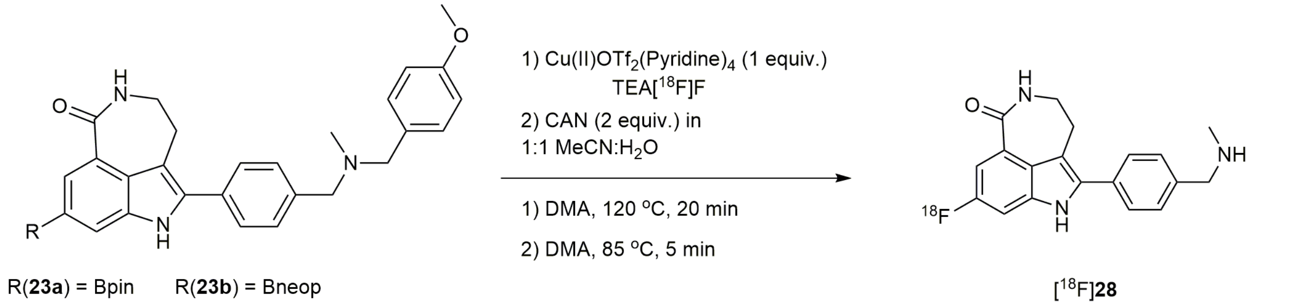

Scheme S1 Reaction scheme for the copper mediated radiofluorination of 23a and 23b to obtain [^{18}F]28.

Preparation of the reagents and reaction procedure

Solution for elution of [^{18}F]fluoride: Was prepared in the same manner as previously described in materials and methods.

Radiolabeling precursor solution: 23a or 23b (15 µmol), was dissolved in anhydrous N,N-Dimethylacetamide (DMA) (0.8 mL), by sonicating at 40 °C until all solids dissolved.

Tetrakis(pyridine)copper(II) triflate solution: copper(II) triflate (15 µmol) was suspended in DMA (0.2 mL), followed by addition of anhydrous pyridine (60 µmol). The mixture was prepared no more than 10 min before reaction start.

Cer ammonium nitrate (CAN) solution: Cerium(IV) ammonium nitrate (CAN, 2mg, 3.6 µmol) is dissolved in a 1:1 mixture of H_2O:MeCN (0.1 mL).

Labeling Procedure

Tetrakis(pyridine)copper(II) triflate solution (0.1 mL) is added to the potassium-15-C-5 [^{18}F]fluoride complex, followed by addition of radiolabeling precursor solution (0.8 mL) in a reactivial with a conical bottom The reactivial is counted, placed on the hotplate, and left to react for 20 min at 120 °C. After the time has elapsed the mixture is removed from the hotplate and placed in an ice bath for 30 seconds. CAN solution is added to the mixture and heated to 85 °C for 5 min. The reaction is quenched by cooling in an ice bath for 2 min. The mixture is diluted 1:1 with either ammonium formate buffer (25 mM, pH = 9.2) or phosphate buffered saline before injection into the HPLC, for which HPLC system C was used.

One-pot borono-deamination radiofluorination

We attempted the sequential borylation followed by direct copper mediated radiofluorination with [^{18}F]F^- by using our optimized conditions. The following general protocol was used to label drug compounds from their amine starting materials:

Aromatic amine (30 µmol) was dissolved in MeCN (150 µL) in a reactivial with a conical bottom. To the solution was added in succession B_2pin_2 or B_2neop_2 (15 µmol), $B(C_6F_5)_3$ (2.5 mol%) and AmylONO (45 µmol). The vial was heated to 40 °C for 15 min. At the same time [^{18}F]F^- (50-200 MBq) was eluted from the target water from the cyclotron and azeotropically dried as described in the article text. After 15 minutes had elapsed the reaction mixture was added to the dried fluoride, followed by a solution of tetrakis(pyridine)copper(II) triflate (30 µmol) and pyridine (120 µmol) in DMA (850 µL). The homogeneous solution was heated to 120 °C for 20 min, and cooled on an ice water. The solution was diluted 1:10 with MeCN and injected into an HPLC system.

Calculation of radiochemical yields (RCY) and quality control

Radiochemical conversions (RCC) were determined by injecting aliquots of diluted reaction mixtures into the analytical HPLC system A, B or C.

For calculation of radiochemical yields (RCY), an activity balance over the reaction was used, i.e. the percentage of activity in the product fraction at the end of syntheses was divided by the starting activity and the fraction was multiplied with 100%. For ease of screening, unreacted fluoride was separated from labelled products via liquid-liquid extraction with Et_2O (1 mL).

A quality control by HPLC was conducted using systems confirm identity and determine radiochemical purity of the products. Product remaining in the aqueous phase after extraction was omitted. The activity corresponding to the product was divided by the total activity at this timepoint and multiplied with 100% to give the RCY.

Alternatively, for [^{18}F]**23d** the area under the peak of the radioactive product was divided by the remaining are of the chromatogram and multiplied by 100% to give the RCY.

Molar activity of the product was calculated from the concentration of the labelled compound in the final formulation as determined by HPLC. In brief, UV calibration curves were recorded using 5 different concentrations of the reference compound. The calibration curves were used to calculate the amount of substance in the isolated product. Total activity of the product was divided by the total amount of substance to obtain molar activities in MBq/nmol.

ICP-MS analysis

Labeled compounds were formulated in 10% EtOH in phosphate buffered saline (PBS) after purification. A sample of the formulated radiotracer was prepared for Cu-determination by ICP-MS by 1:9 dilution in 2% (V/V) ultra-pure HNO_3, and injected to the ICP-MS system for copper determination. Samples were analyzed on an Agilent 8900 #100 ICP-MS instrument, in HE-KED MS-MS mode for ^{63}Cu and ^{65}Cu. The instrument was calibrated by measurement of standards containing 2.26, 20.2 and 200 µg/mL Cu, which were prepared identically to formulated samples.

2 Supplementary Figures and Tables

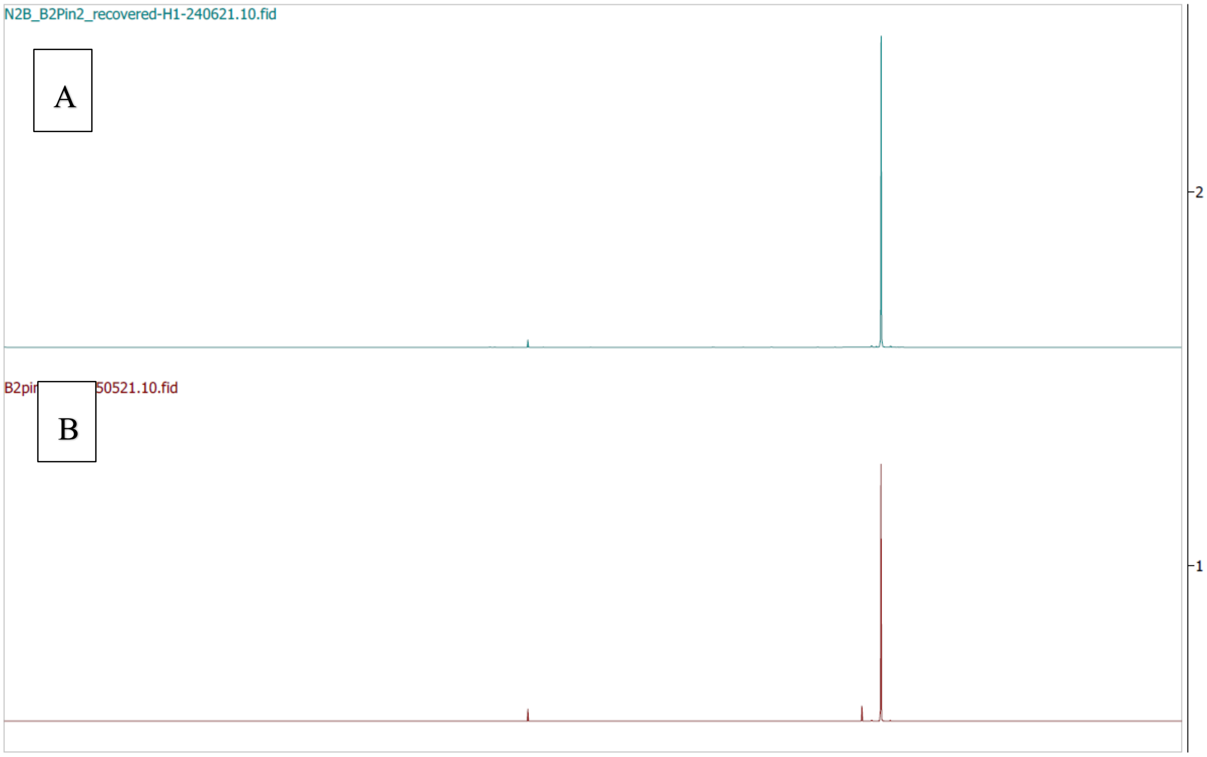

Figure S1. ^{1}H-NMR spectra of B_2pin_2 recovered (A) from our reaction conditions, and from a commercial source (B)

Figure S2. Synthesis route to 23a and 23b.

I) Boc_2O, THF, 60 °C; II) $POCl_3$, DMF, 0 °C to r.t.; III) $MeNO_2$, NH_4OAc, 60 °C; IV) $NaBH_4$, MeOH:THF (1:5); V) 1) Raney Ni, H_2 (g) 1 atm., MeOH, rt. 2) MeOH, Reflux; VI) THF:DCM (1:1), $PyrBr_3$, 0 °C to r.t.; VII) 4-Formylphenylboronic acid, Pd(dppf)Cl_2, Na_2CO_3, DMAc, 90 °C. VIII) 4-Methoxy-N-methylbenzyl amine, $NaB(OAc)_3H$ DMF, r.t.; IX) DCM:TFA (4:1); X) B_2pin_2/B_2neop_2, Amylnitrite, $B(PhF_5)_3$, MeCN, AcOH, 40 °C.

Table S1. Chemical shifts for relevant compounds present in the reaction mixture, in screening runs done with 4-nitro aniline as starting material

Compound	Chemical shifts aromatic region (ppm)		Chemical shifts aliphatic region (ppm)	
4-Nitrophenylboronic acid pinacol ester	d, 8.11	d, 7.89	s, 1.29	-
Diazonium intermediate	d, 8.21	d, 7.53	-	-

<table>
<tr><td>4-Nitrobenzene</td><td>d, 8.16</td><td>t, 7.63</td><td>t, 7.48</td><td>-</td><td>-</td></tr>
<tr><td>4-Nitroaniline</td><td>d, 7.96</td><td colspan="2">d, 6.54</td><td>s, 4.99</td><td>-</td></tr>
<tr><td>B_2pin_2</td><td>-</td><td colspan="2">-</td><td>s, 1.18</td><td>s,1.17</td></tr>
<tr><td>4-Nitrophenylboronic acid neopentyl ester</td><td>d, 8.11</td><td colspan="2">d, 7.89</td><td>s, 3.72</td><td>s, 0.95</td></tr>
<tr><td>B_2neop_2</td><td>-</td><td colspan="2">-</td><td>3.55-351</td><td>0.89-0.87</td></tr>
</table>

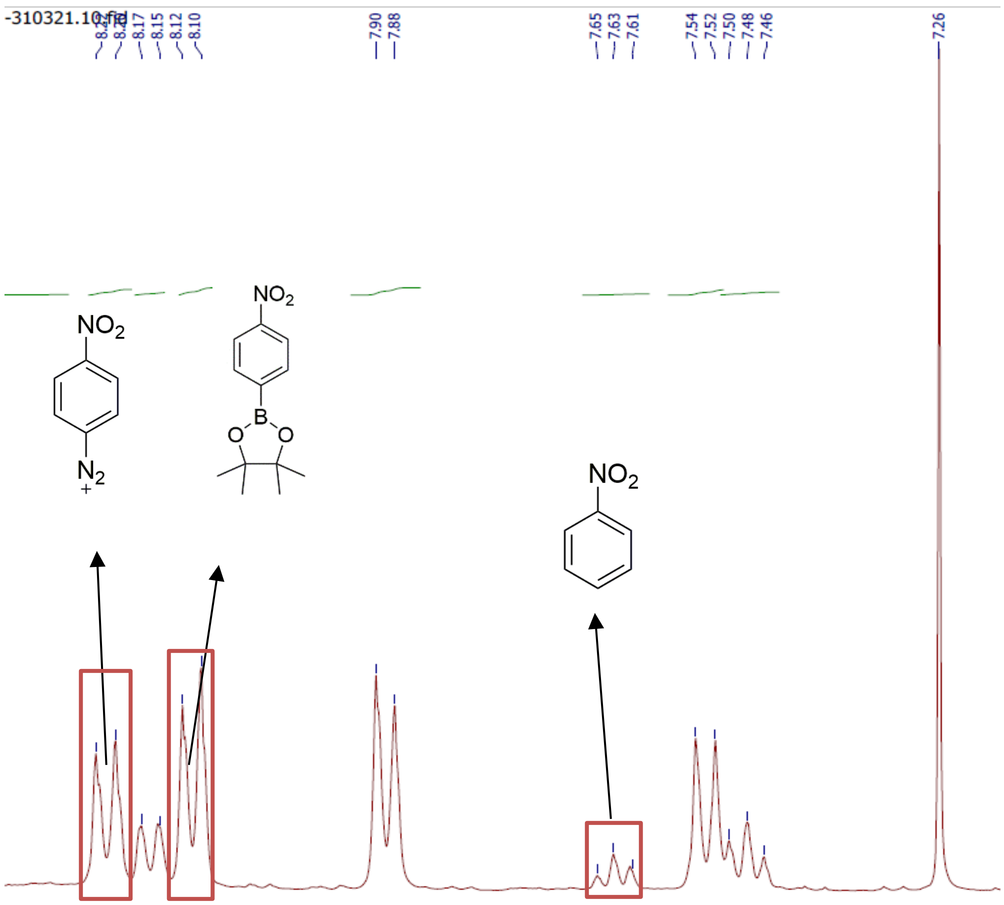

Figure S3. Identification of signals in the aromatic region from important intermediates and products in the reaction mixture

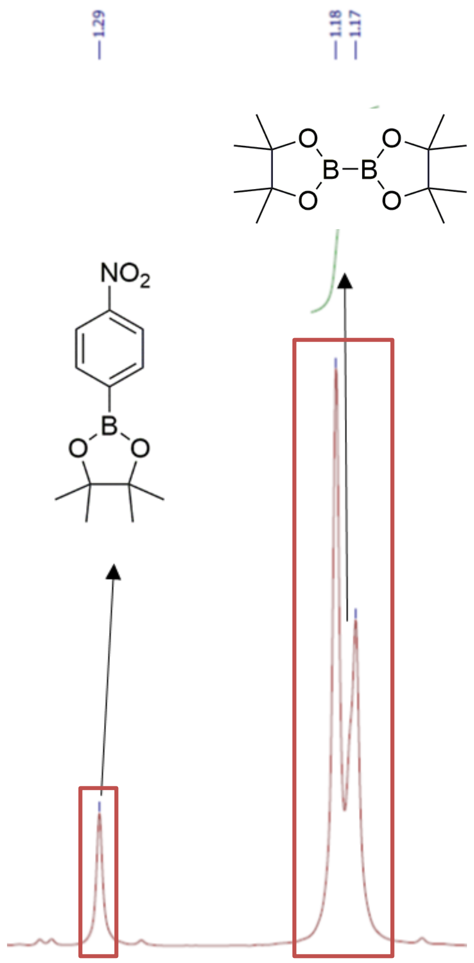

Figure S4. Identification of signals in the aliphatic region from important intermediates and products in the reaction mixture

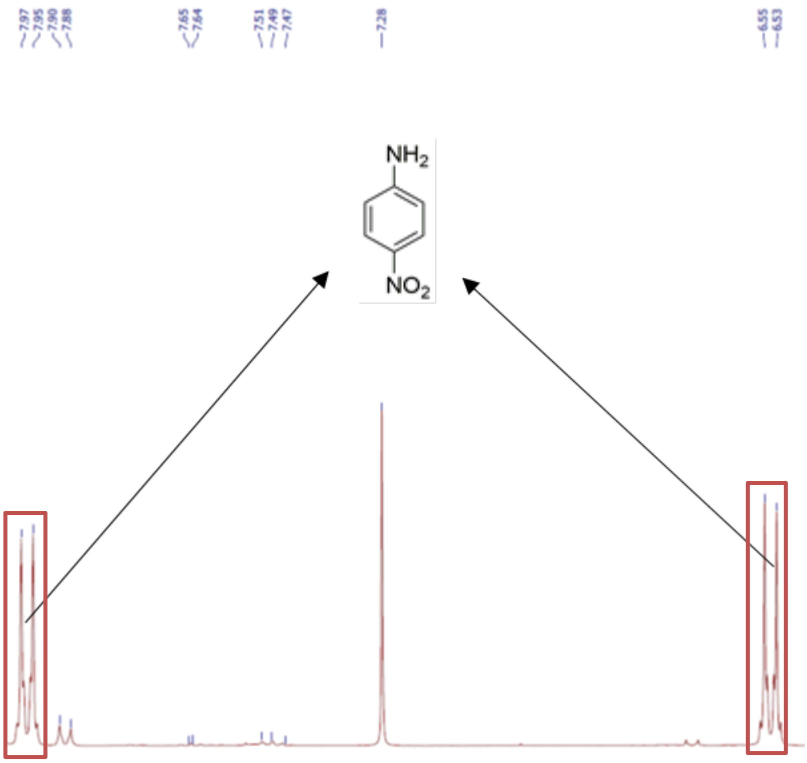

Figure S5. Signals from unreacted starting aniline

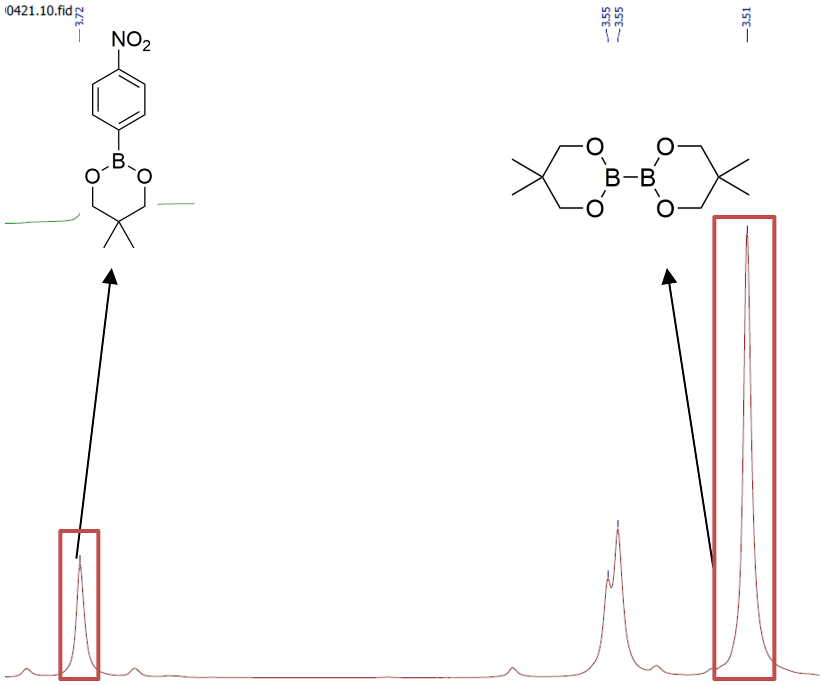

Figure S6. First set of signals of the neopenyl derivatives

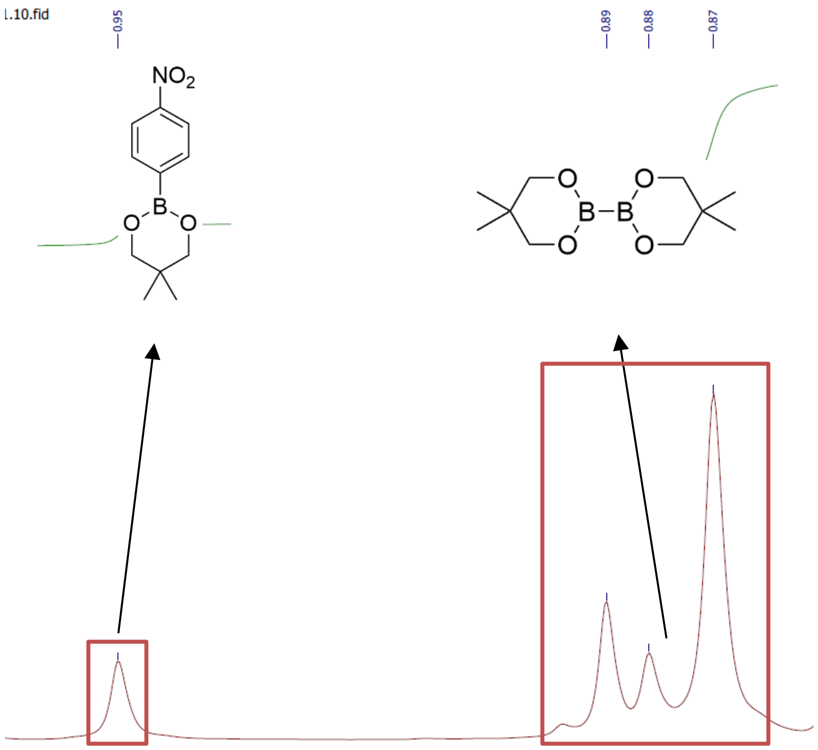

Figure S7. Second set of signals of the neopentyl derivatives

Analytical data

Radio-HPLC chromatograms and fluoride reference compounds

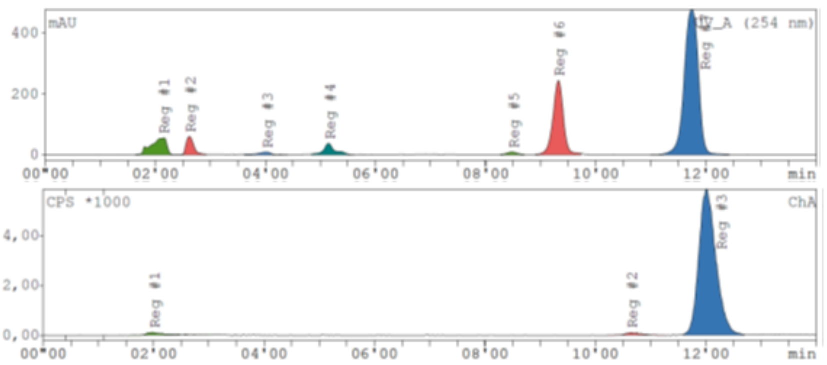

Substance	t_R / min.sec	Area (absolute) / mV^2	Area (relative) %
**25**	11.45	8562.511	60.02
[^{18}F]**25**	12.00	125926.1	86.68

Figure S8. Radiofluorination of precursor 19a. Top channel = UV trace of fluoride reference 25; Bottom channel = Activity trace of reaction mixture. MeCN-H_2O; 40:60. Flow rate 1 ml/min. Injection volume 10 uL.

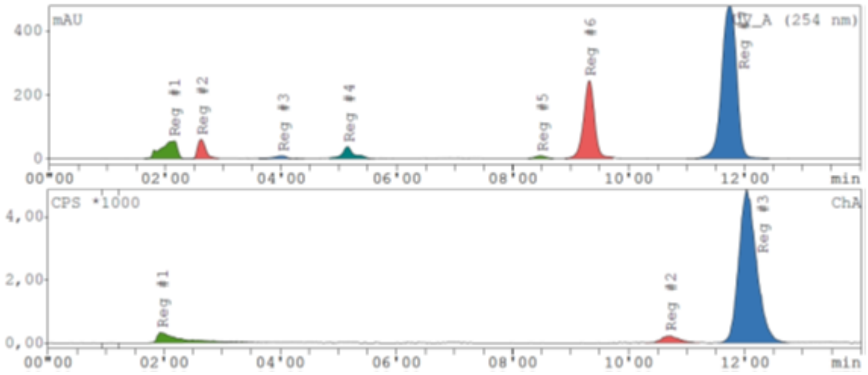

Substance	t_R / min.sec	Area (absolute) / mV^2	Area (relative) %
**25**	11.45	8562.511	60.02
[^{18}F]**25**	12.04	102336.0	88.47

Figure S9. From compound 19b. Top channel = UV trace of fluoride reference 25; Bottom channel = Activity trace of reaction mixture. MeCN-H_2O; 40:60. Flow rate 1 ml/min. Injection volume 10 uL.

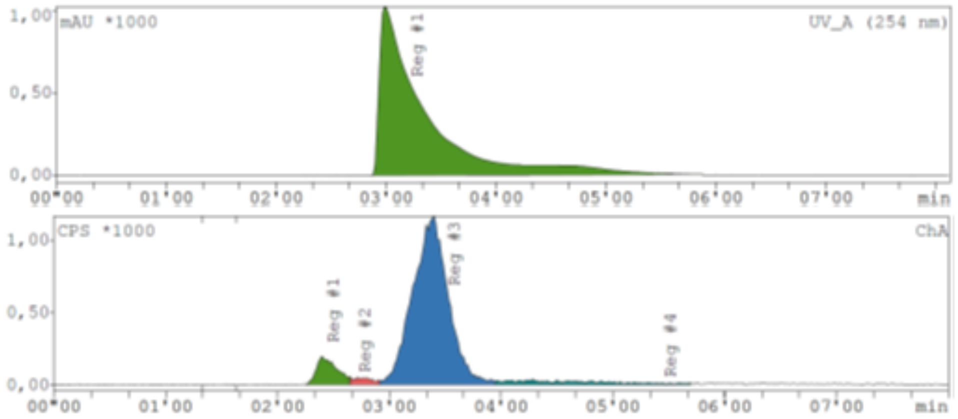

Substance	t_R / min.sec	Area (absolute) / mV^2	Area (relative) %
**26**	3.01	45643.32	54.89
[^{18}F]**26**	3.23	25734.92	84.12

Figure S10. Radiofluorination of precursor 20a. Top channel = UV trace of reference compound 26; Bottom channel = Activity trace of reaction mixture.. MeCN-H_2O; 80:20. Flow rate 1 ml/min. Injection volume 10 uL.

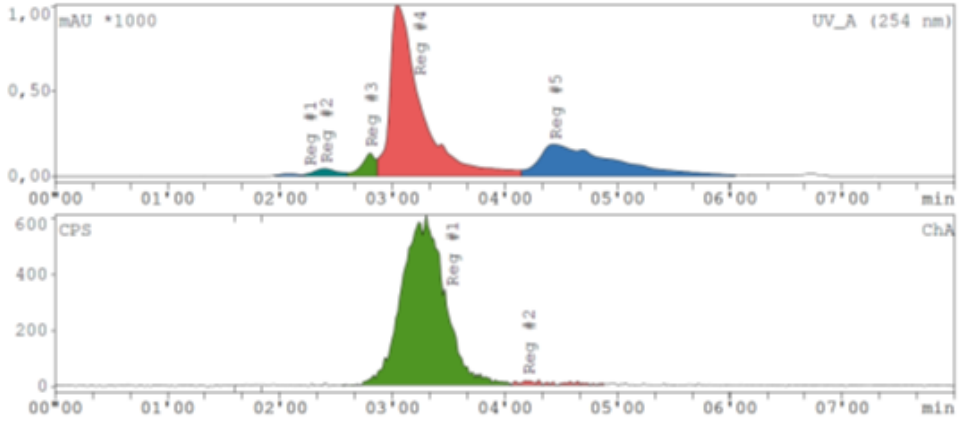

Substance	t_R / min.sec	Area (absolute) / mV^2	Area (relative) %
**26**	3.03	19593.55	64.88
[^{18}F]**26**	3.16	16101.83	97.83

Figure S11. Radiofluorination of precursor 20b. Top channel = UV trace of reference compound 26; Bottom channel = Activity trace of reaction mixture. MeCN-H_2O; 80:20. Flow rate 1 ml/min. Injection volume 10 uL.

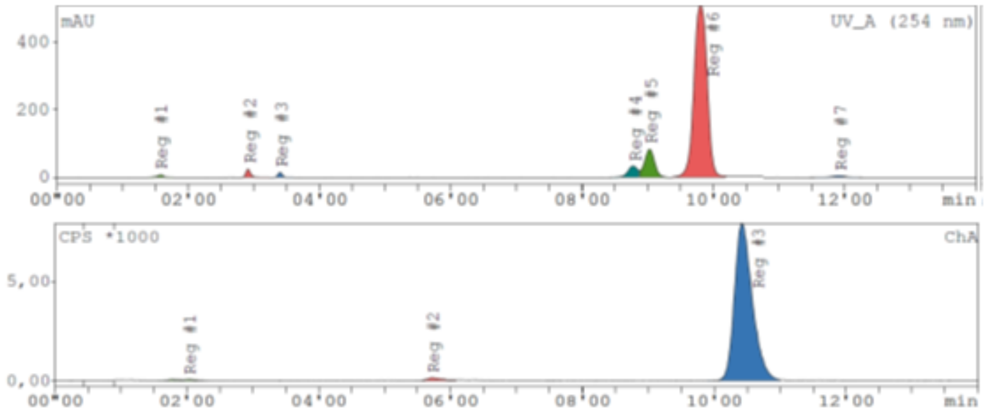

Substance	t_R / min.sec	Area (absolute) / mV^2	Area (relative) %
**27**	9.50	147987.7	97.56
[^{18}F]**27**	10.25	7107.790	82.37

Figure S12. Radiofluorination of precursor 21a. Top channel = UV trace of reference compound 27; Bottom channel = Activity trace. MeCN-H_2O; 55:45. Flow rate 1 ml/min. Injection volume 10 uL.

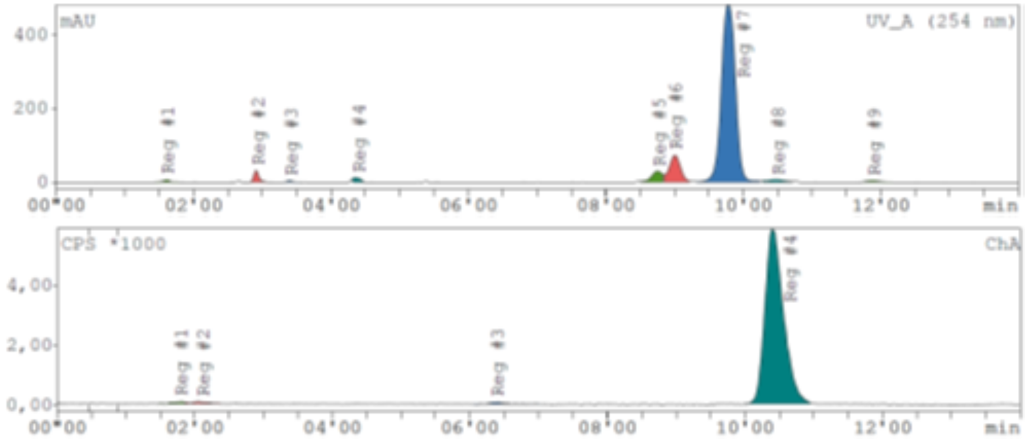

Substance	t_R / min.sec	Area (absolute) / mV^2	Area (relative) %
**27**	9.47	6786.450	80.93
[^{18}F]**27**	10.24	107829.6	97.80

Figure S13. Radiofluorination of precursor 21b. Top channel = UV trace of reference compound 27; Bottom channel = Activity trace of reaction mixture. MeCN-H_2O; 55:45. Flow rate 1 ml/min. Injection volume 10 uL.

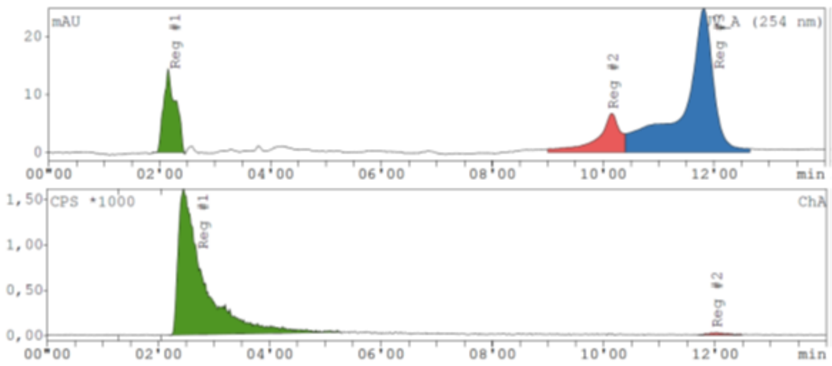

Substance	t_R / min.sec	Area (absolute) / mV^2	Area (relative) %
**28**	11.49	924.3490	63.87
[18F]**28**	12.01	652.81	1.26

Figure S14. Radiofluorination of precursor 23b. Top channel = UV trace of reference compound 28; Bottom channel = Activity trace of reaction mixture. MeCN-H_2O; 80:20. Flow rate 1 ml/min. Injection volume 10 uL.

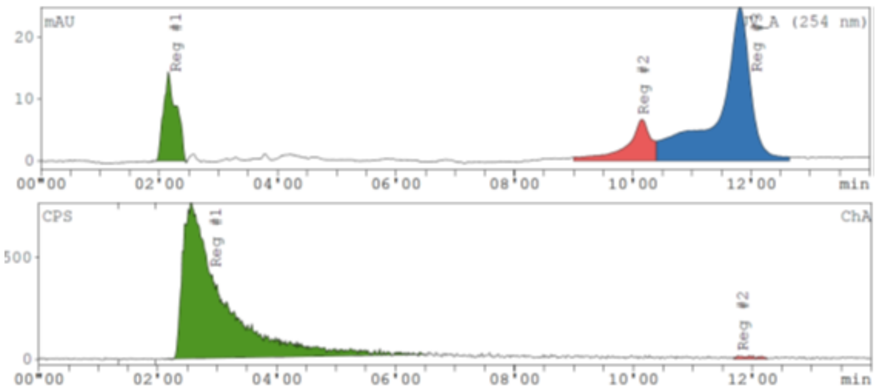

Substance	t_R / min.sec	Area (absolute) / mV^2	Area (relative) %
**28**	11.49	924.3490	63.87
[18F]**28**	11.59	334.69	0.93

Figure S15. Radiofluorination of precursor 23b. Top channel = UV trace of reference compound 28; Bottom channel = Activity trace of reaction mixture. Ammonium formate buffer (25 mM, pH=9.2)-MeOH-MeCN; 60:30:10 1.0 mL/min. Flow rate 1 ml/min. Injection volume 10 uL.

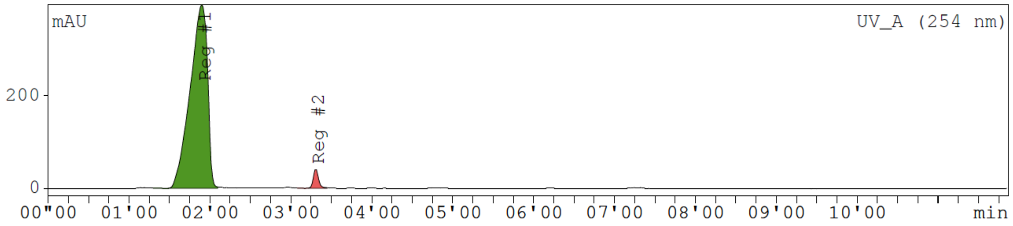

Substance	t_R / min.sec	Area (absolute) / mV^2	Area (relative) %
**$B(C_6F_5)_3$**	1.54	5882.179	98.96

Figure S16. HPLC run of $B(C_6F_5)_3$ at 0.1 mg/mL. Top channel = UV trace. MeOH-MeCN; 65:45 1.0 mL/min. Flow rate 1 ml/min. Injection volume 10 uL.

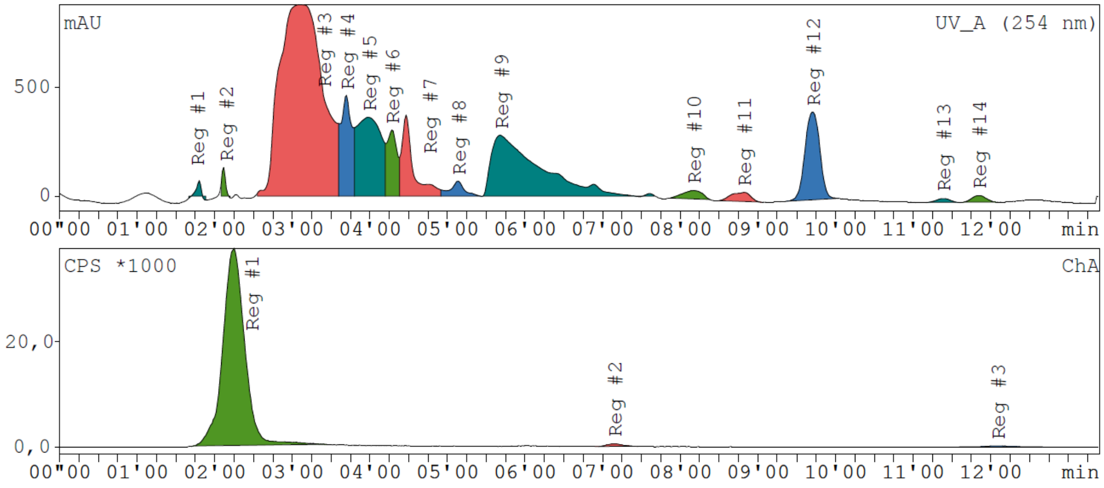

Substance	t_R / min.sec	Area (absolute) / mV^2	Area (relative) %
[^{18}F]**25**	12.05	5119.1	0.68

Figure S17. One pot borono-deamination/radiofluorination on aminoglutetimide with B_2pin_2 as boron source. Top channel = UV trace of reaction mixture; Bottom channel = Activity trace of reaction mixture. MeOH-MeCN; 40:60 1.0 mL/min. Flow rate 1 ml/min. Injection volume 10 uL.

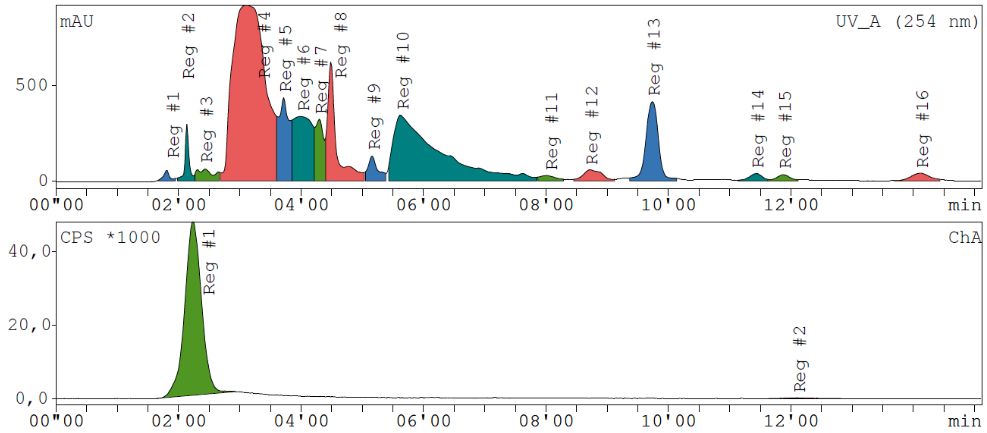

Substance	t_R / min.sec	Area (absolute) / mV^2	Area (relative) %
[^{18}F]**25**	12.06	6414.3	0.71

Figure S18. One pot borono-deamination/radiofluorination on aminoglutetimide with B_2neop_2 as boron source. Top channel = UV trace of reaction mixture; Bottom channel = Activity trace of reaction mixture. MeOH-MeCN; 40:60 1.0 mL/min. Flow rate 1 ml/min. Injection volume 10 uL.

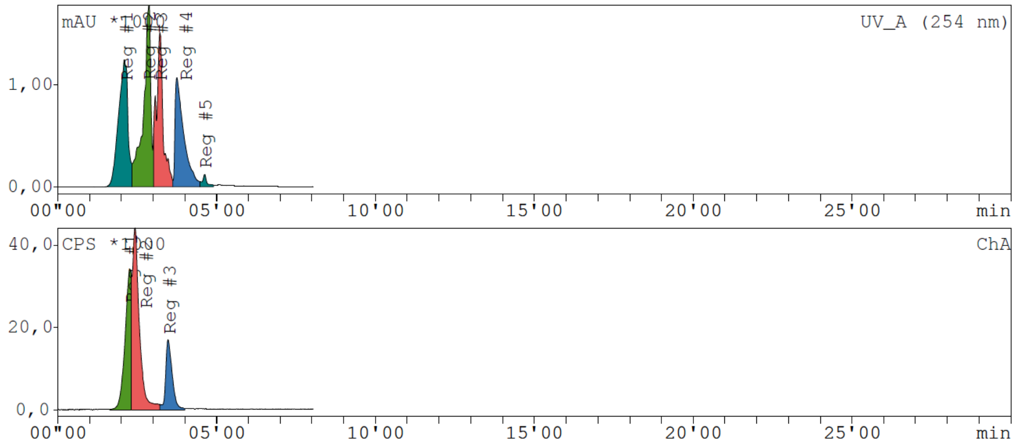

Substance	t_R / min.sec	Area (absolute) / mV^2	Area (relative) %
[^{18}F]**26**	3.29	243724.2	17.52

Figure S19. One pot borono-deamination/radiofluorination on sulfamethoxazole with B_2pin_2 as boron source. Top channel = UV trace of reaction mixture; Bottom channel = Activity trace of reaction mixture. MeOH-MeCN; 80:20 1.0 mL/min. Flow rate 1 ml/min. Injection volume 10 uL.

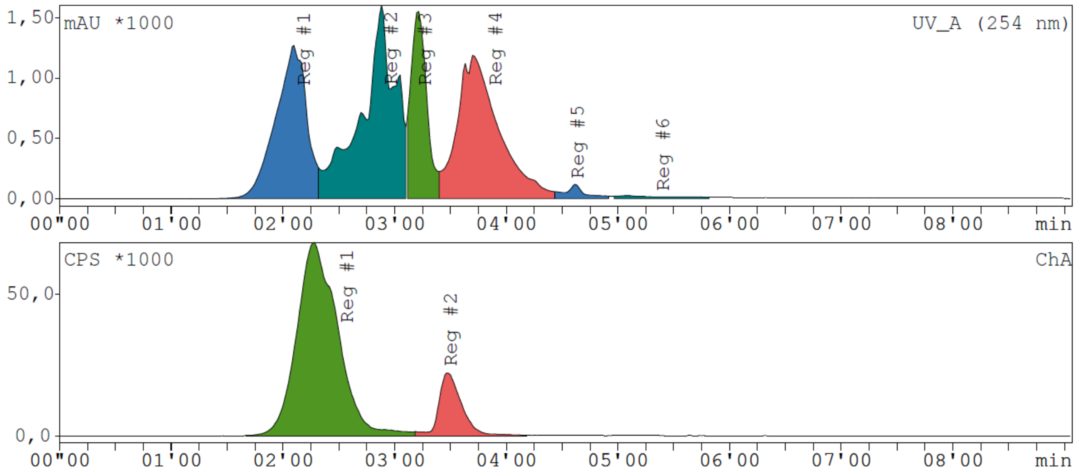

Substance	t_R / min.sec	Area (absolute) / mV^2	Area (relative) %
[^{18}F]**26**	3.29	319859	15.47

Figure S20. One pot borono-deamination/radiofluorination on sulfamethoxazole with B_2pin_2 as boron source. Top channel = UV trace of reaction mixture; Bottom channel = Activity trace of reaction mixture. MeOH-MeCN; 80:20 1.0 mL/min. Flow rate 1 ml/min. Injection volume 10 uL.

NMR Spectra

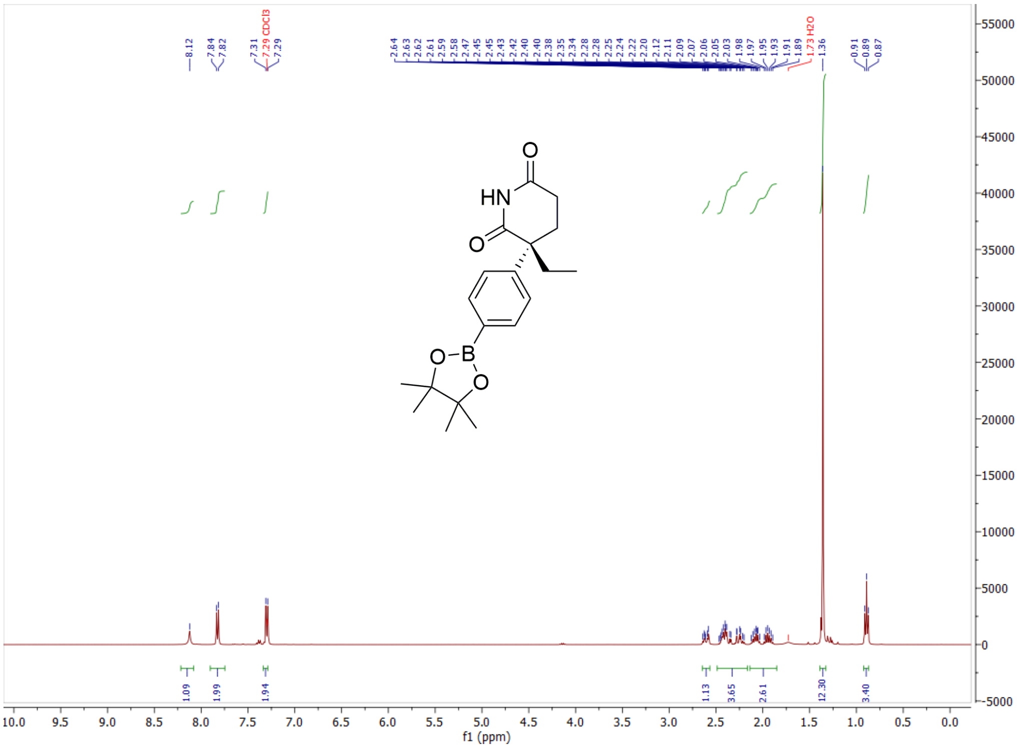

Figure S21. ^{1}H-NMR of compound 19a

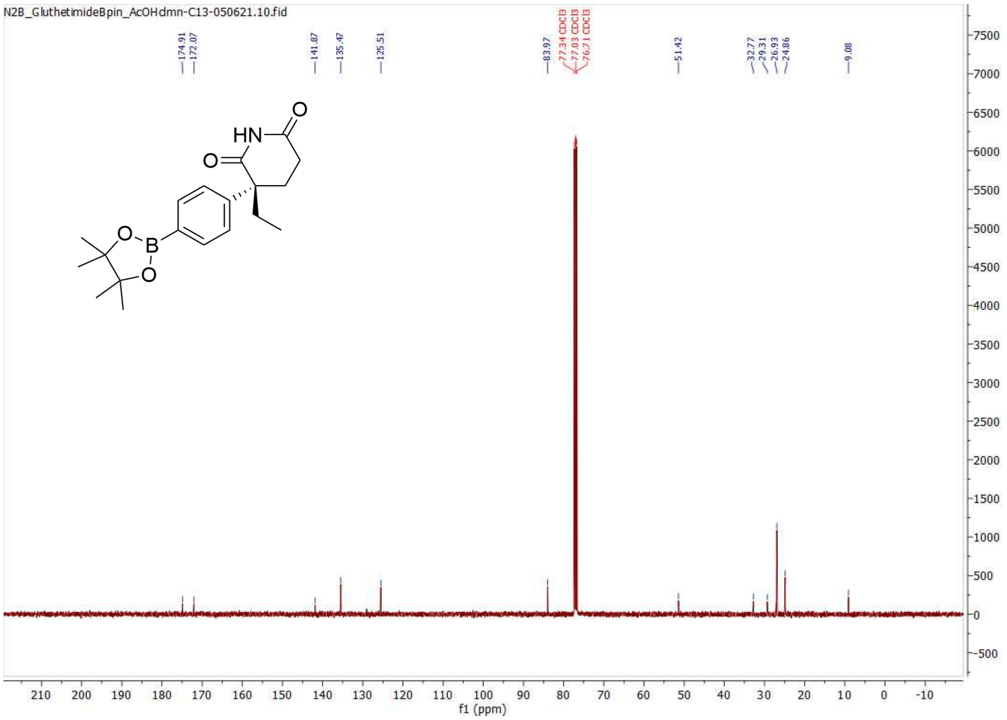

Figure S22. ^{13}C-NMR of compound 19a

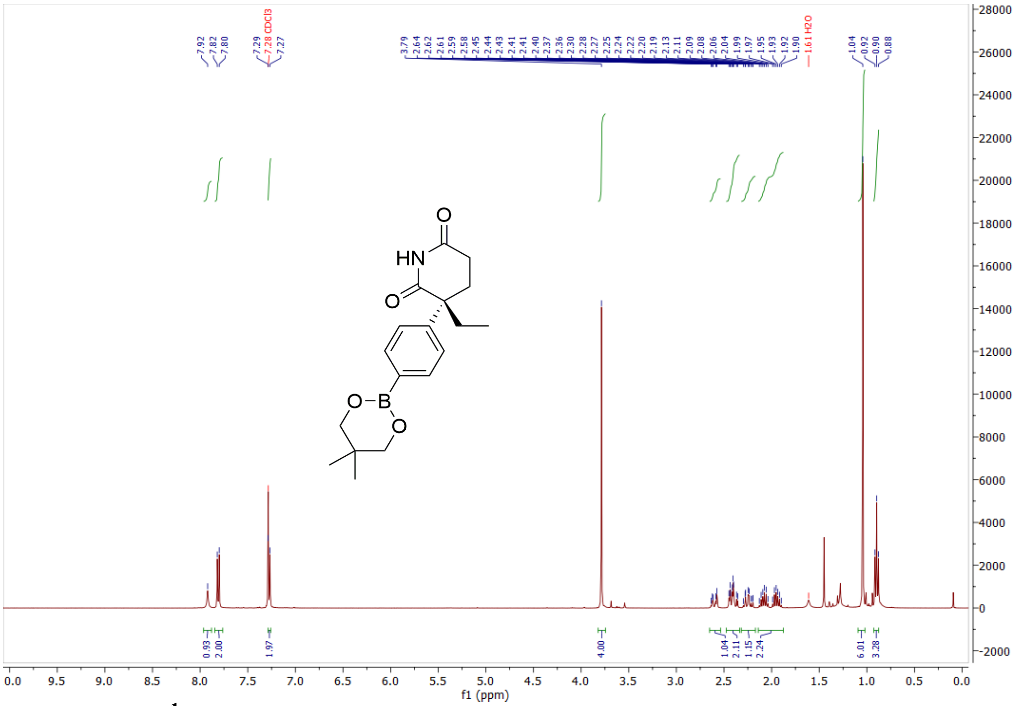

Figure S23. ^{1}H-NMR of compound 19b

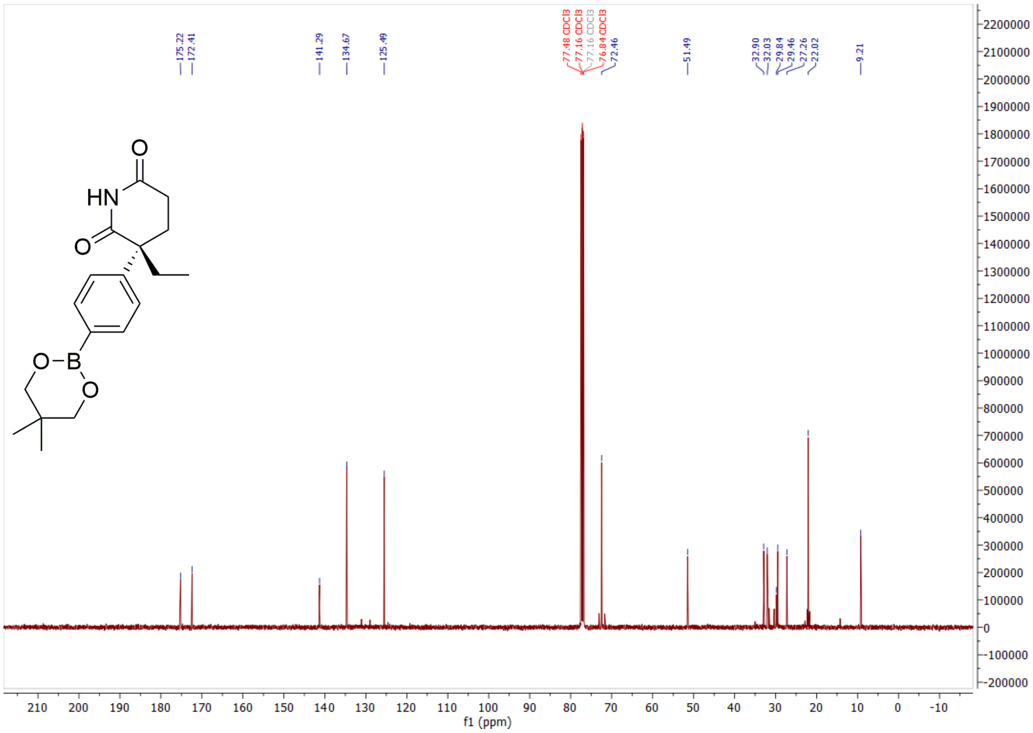

Figure S24. ^{13}C-NMR of compound 19b

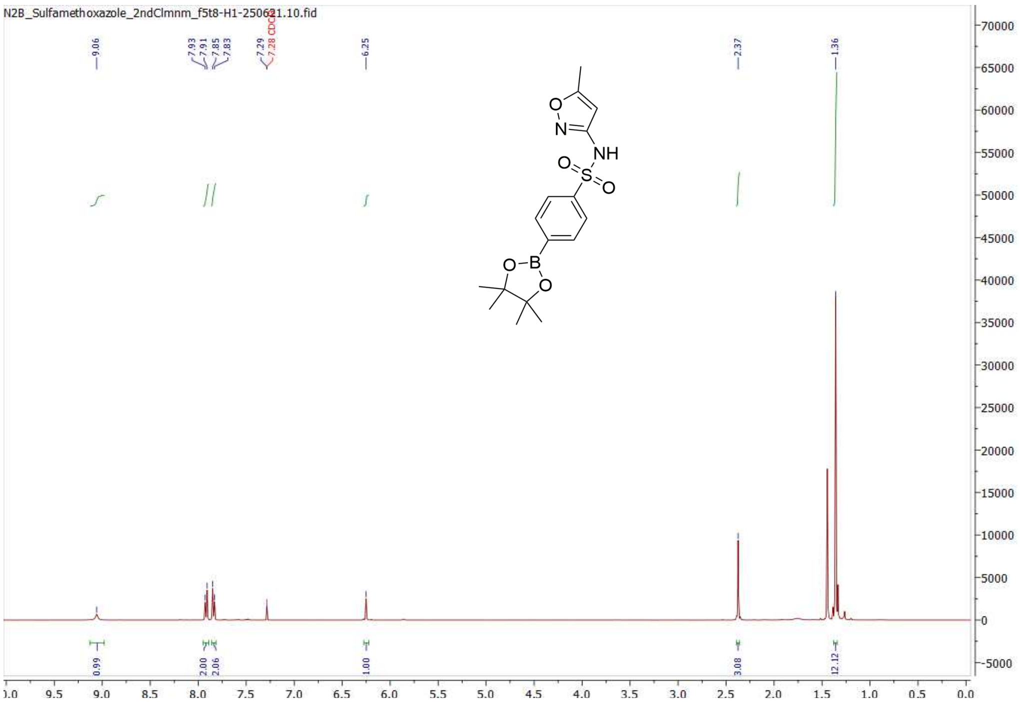

Figure S25. ^{1}H-NMR of compound 20a

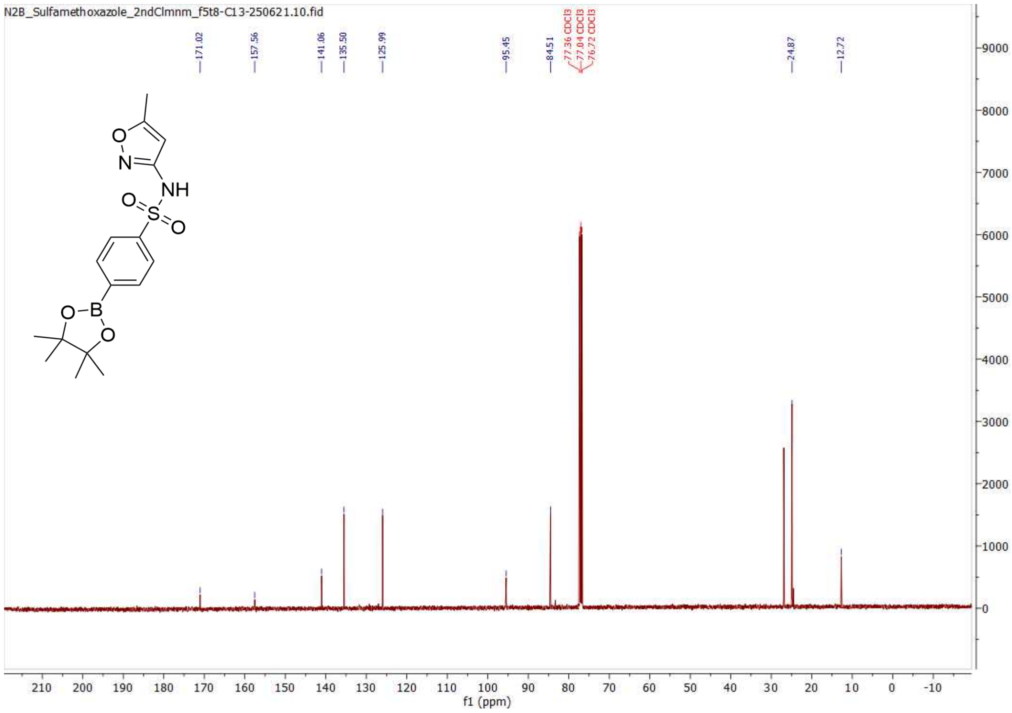

Figure S26. ^{13}C-NMR of compound 20a

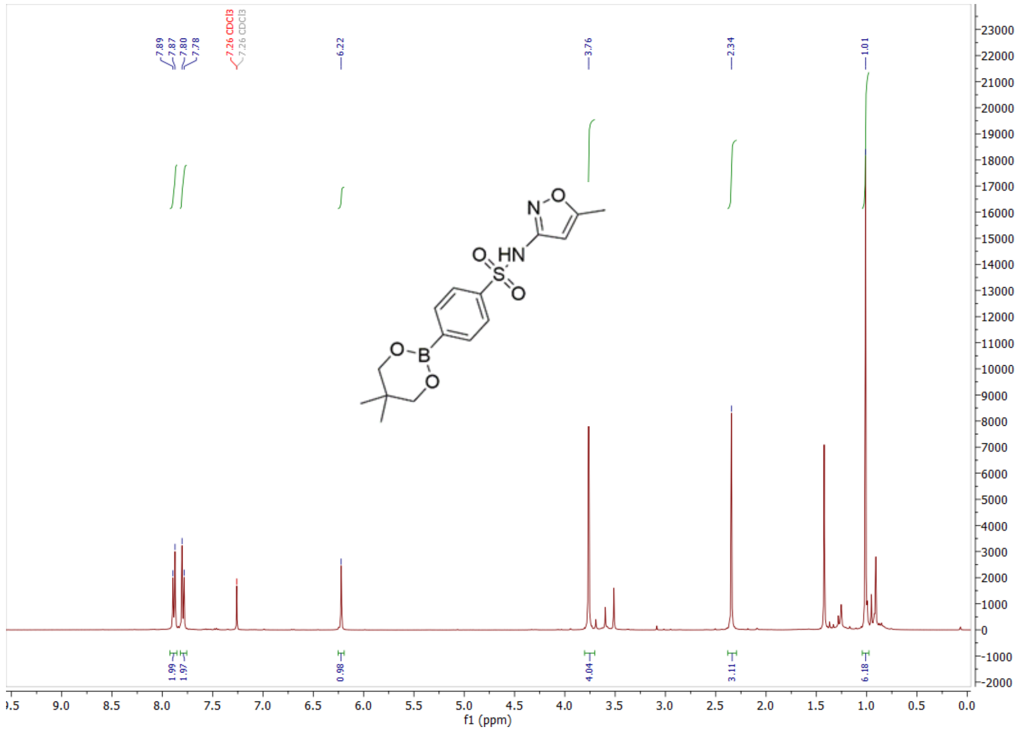

Figure S27. ^{1}**H-NMR of compound 20b**

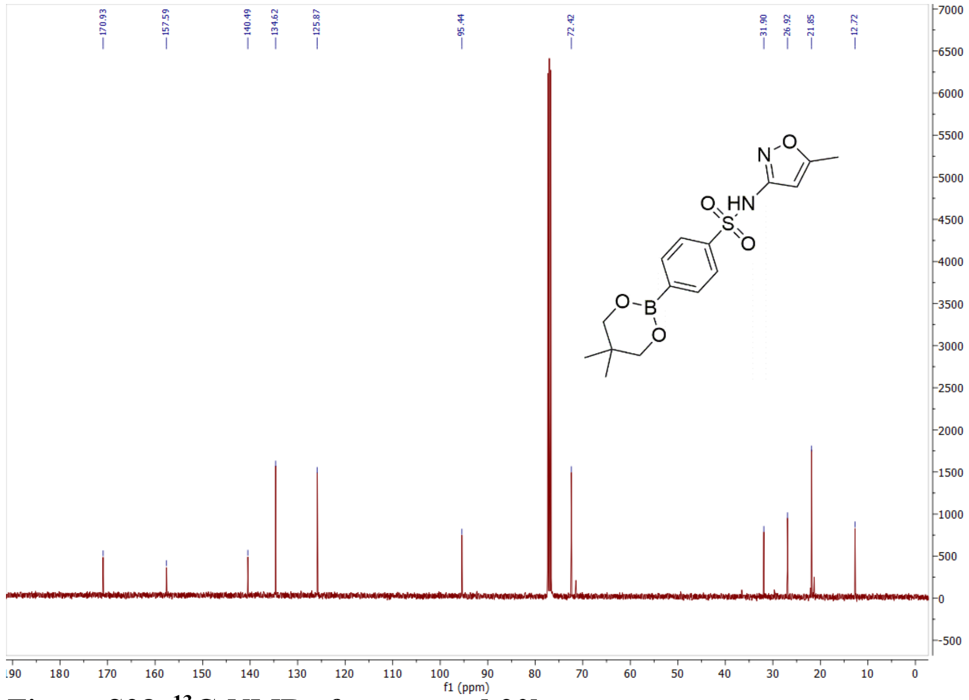

Figure S28. ^{13}**C-NMR of compound 20b**

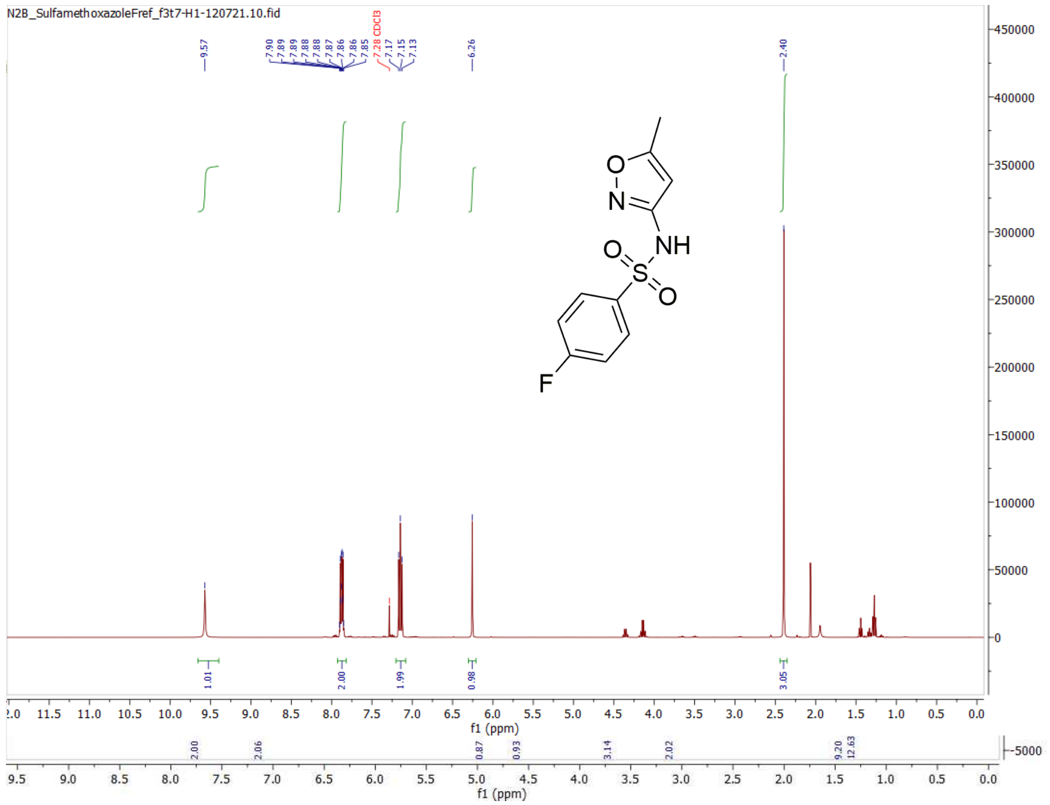

Figure S29. ^{1}H-NMR of compound 26

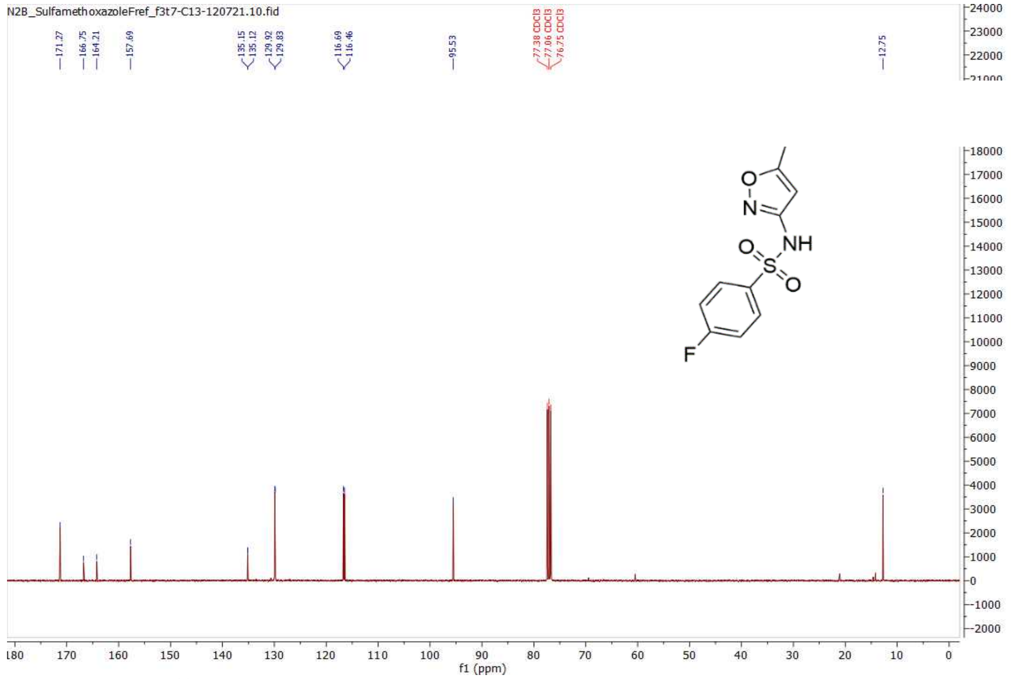

Figure S30. ^{13}C-NMR of compound 26

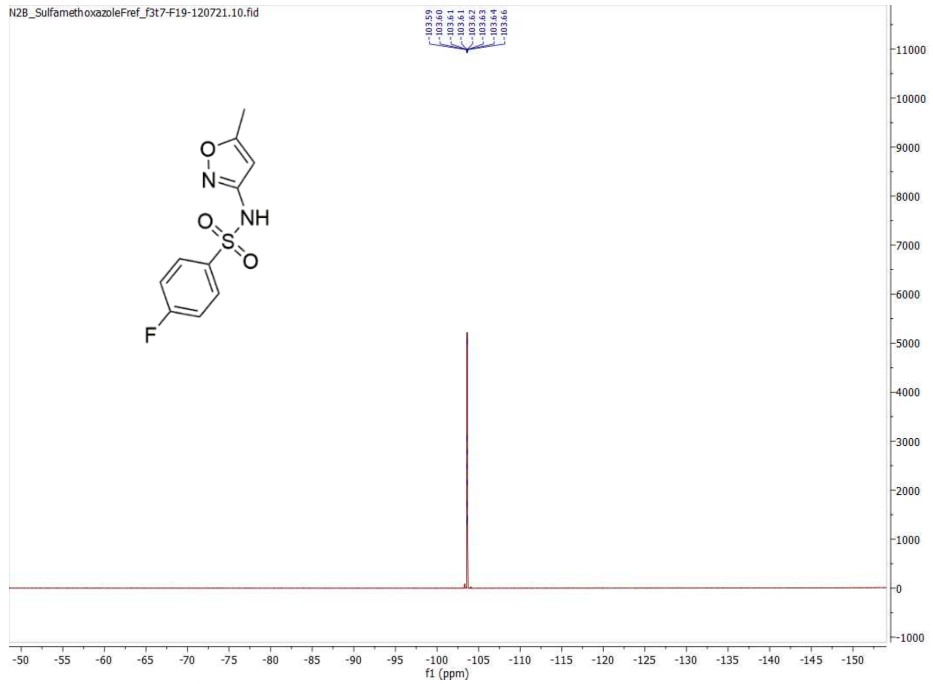

Figure S31 ^{19}F-NMR of compound 26

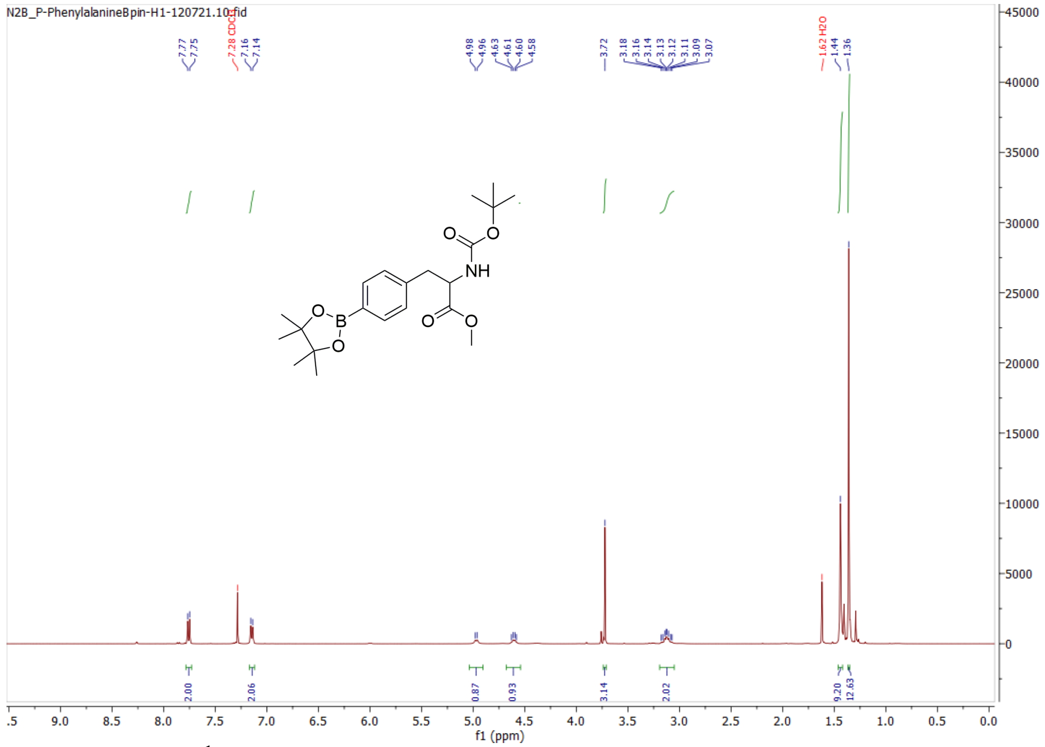

Figure S32 ^{1}H-NMR of compound 21a

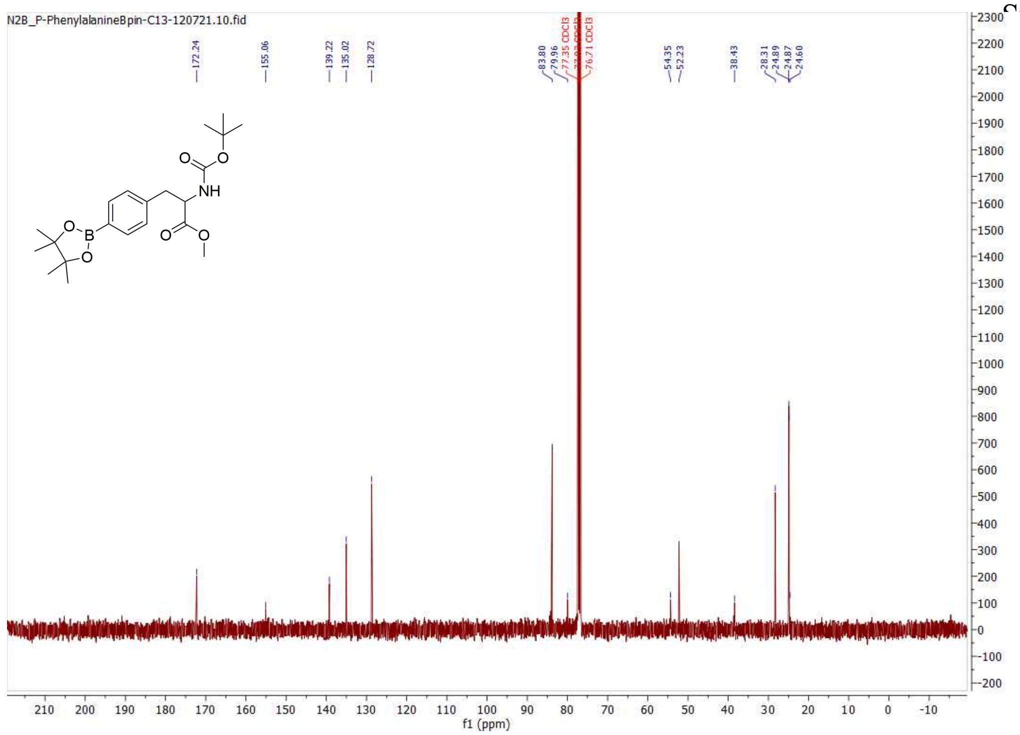

Figure S33. **^{13}C-NMR of compound 21a**

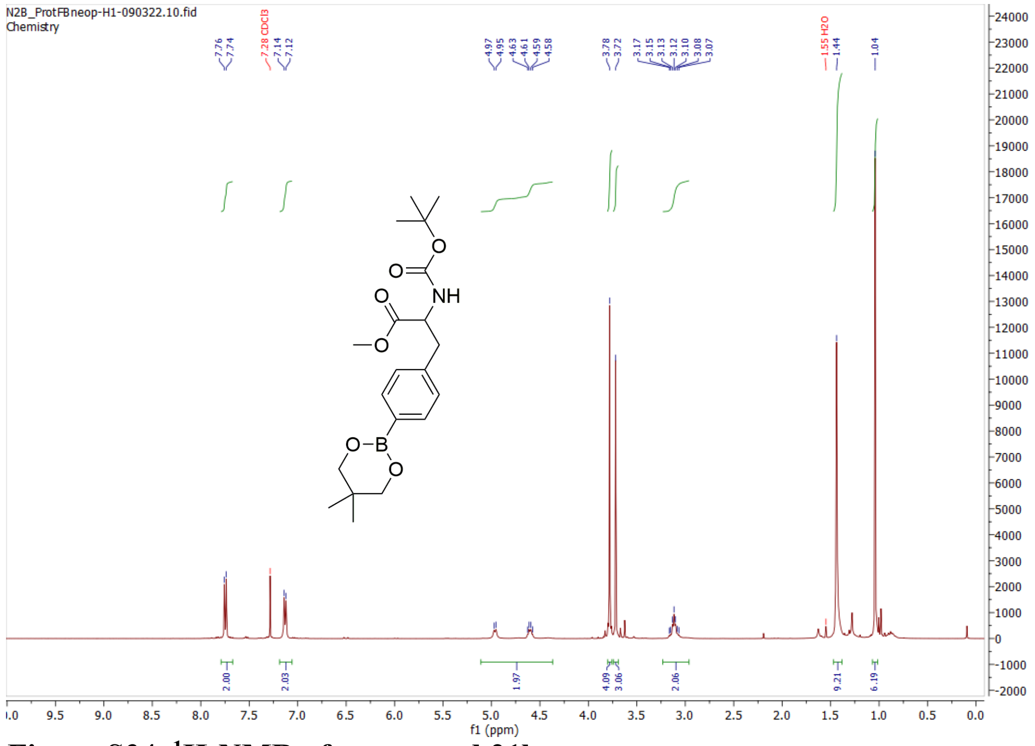

Figure S34. **^{1}H-NMR of compound 21b**

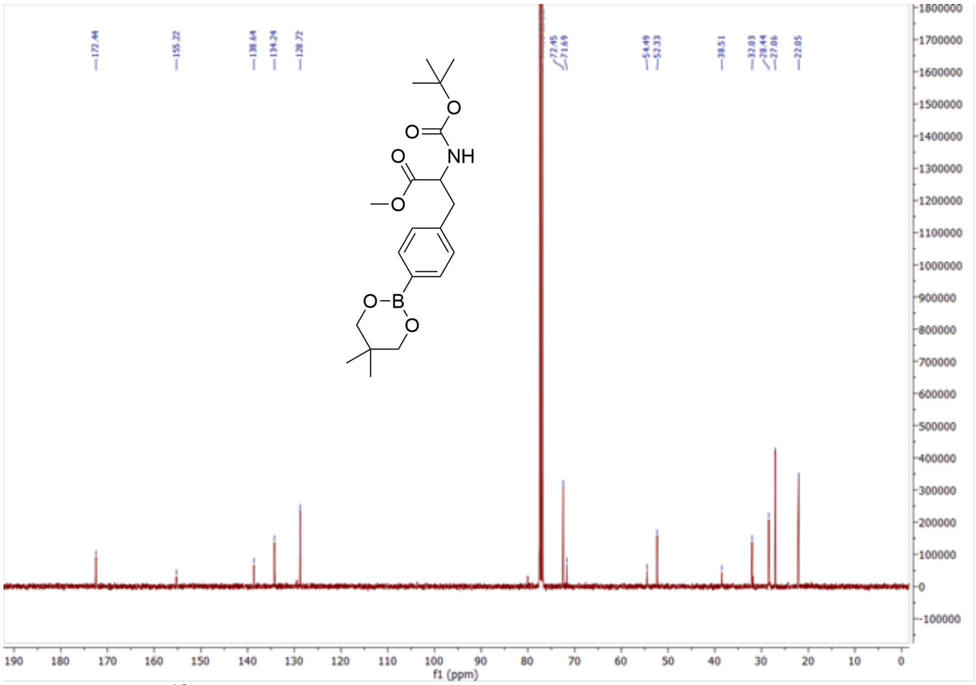

Figure S35. ^{13}C-NMR of compound 21b

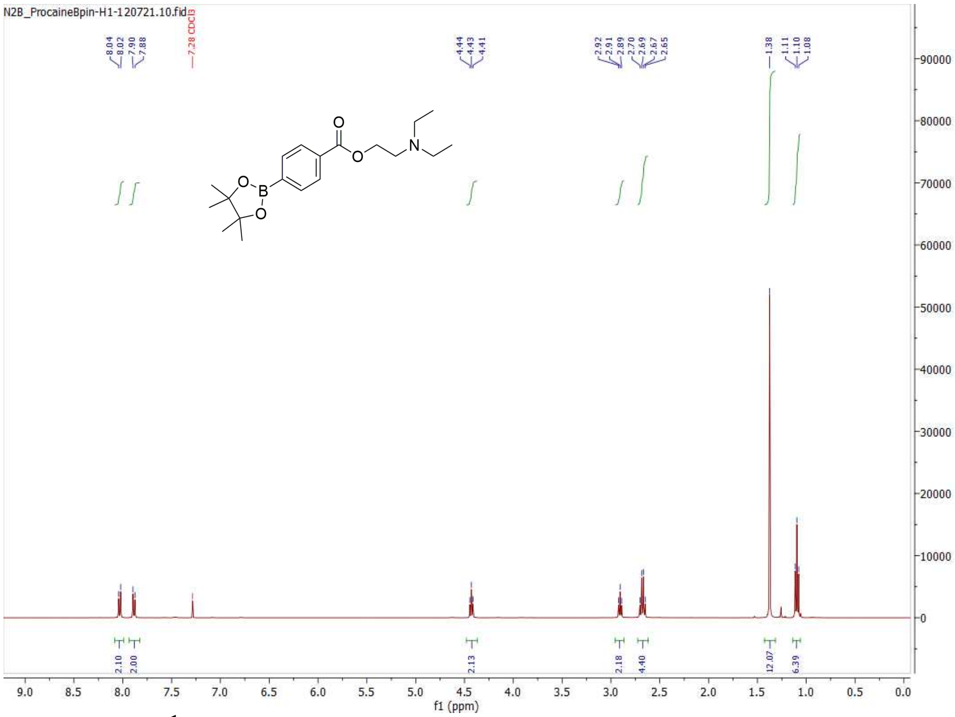

Figure S36. ^{1}H-NMR of compound 22a.

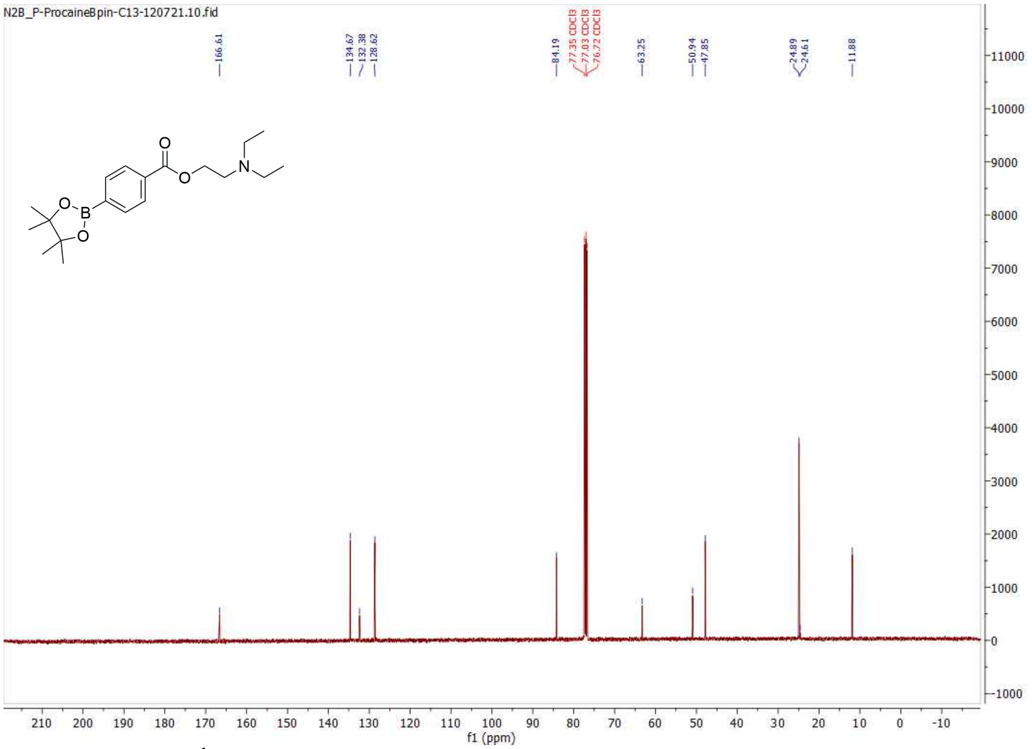

Figure S37. **^{1}H-NMR of compound 22a**

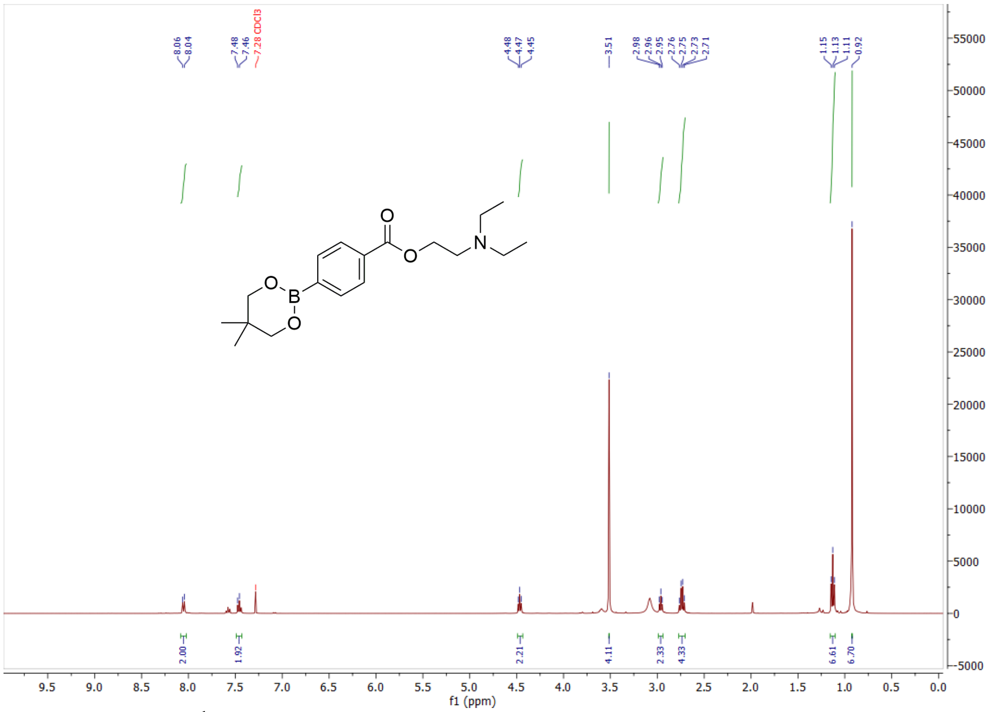

Figure S38. **^{1}H-NMR of compound 22b.**

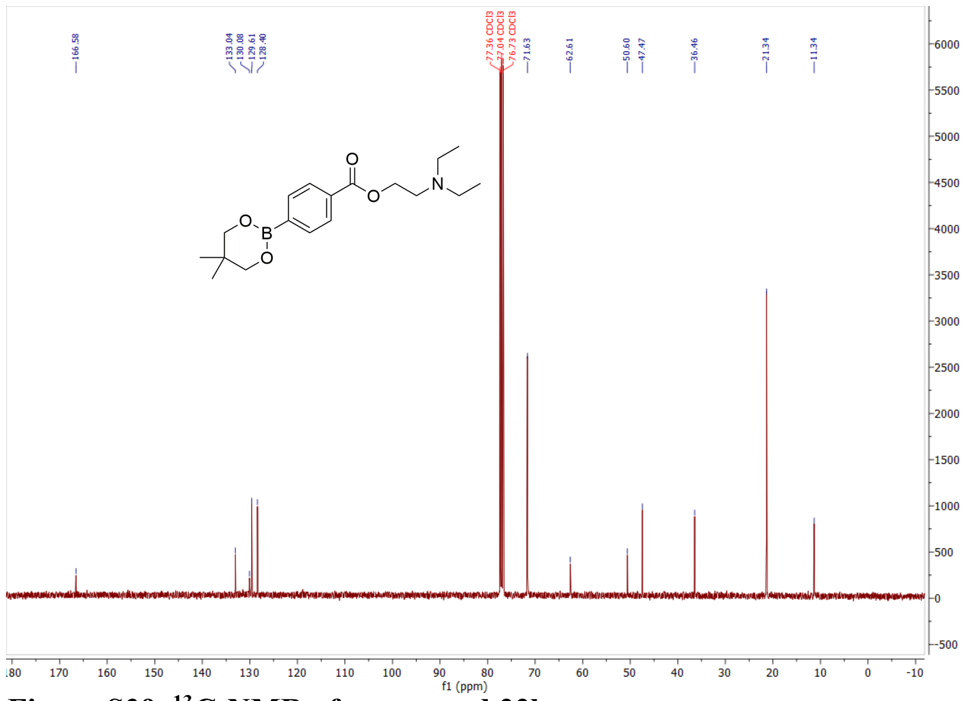

Figure S39. ^{13}C-NMR of compound 22b.

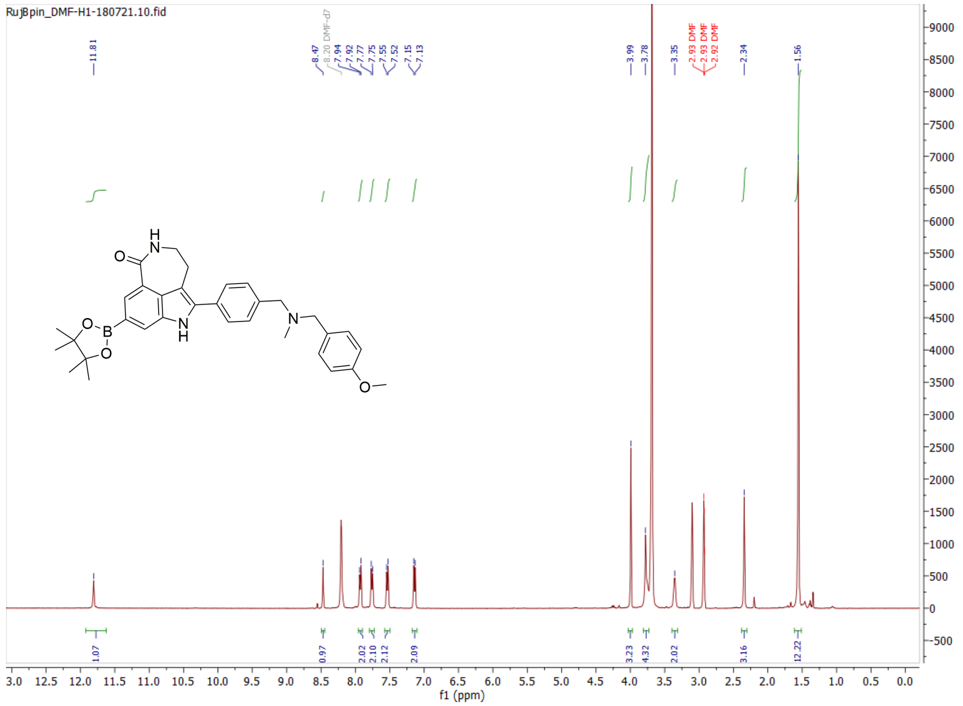

Figure S40. ^{1}H-NMR of compound 23a

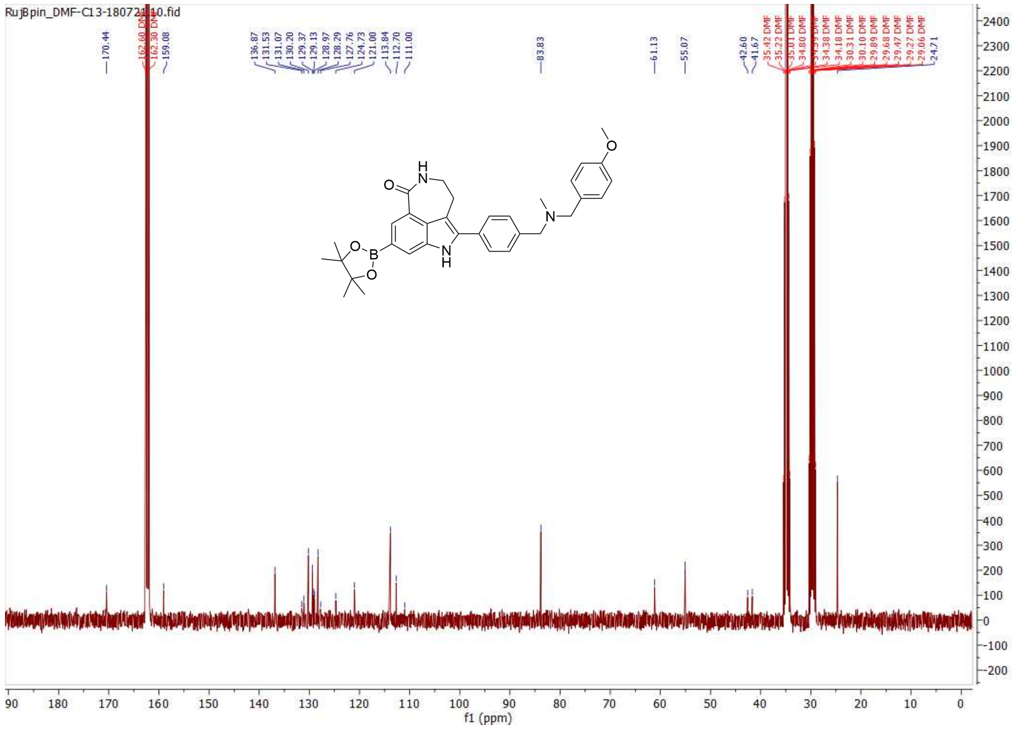

Figure S41. ^{13}C-NMR of compound 23a

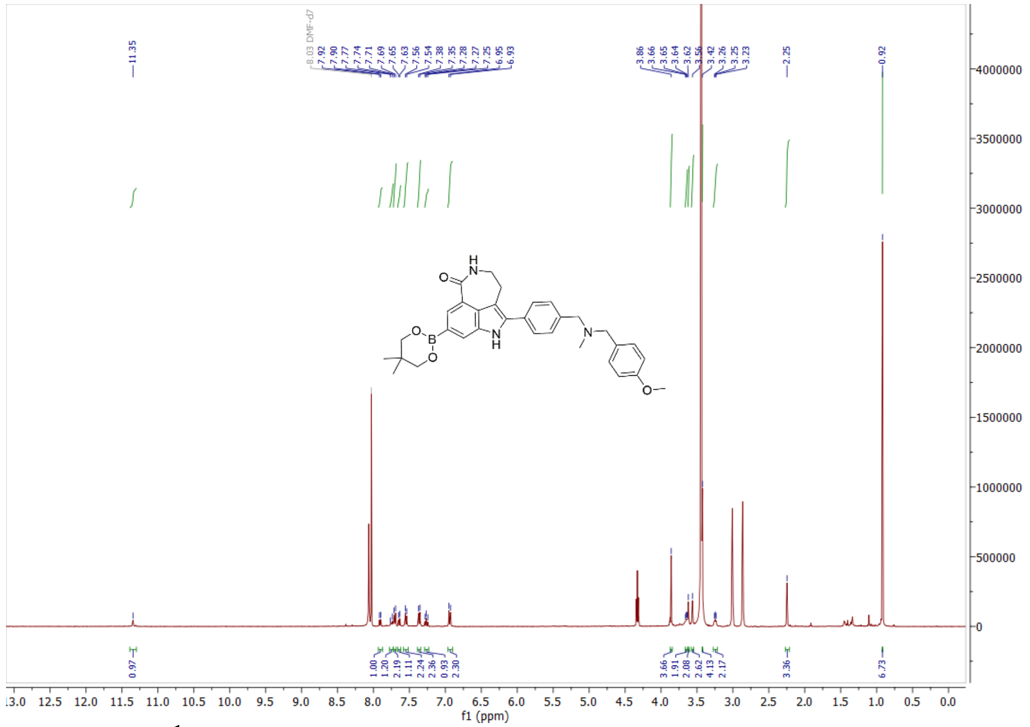

Figure S42. ^{1}H-NMR of compound 23b

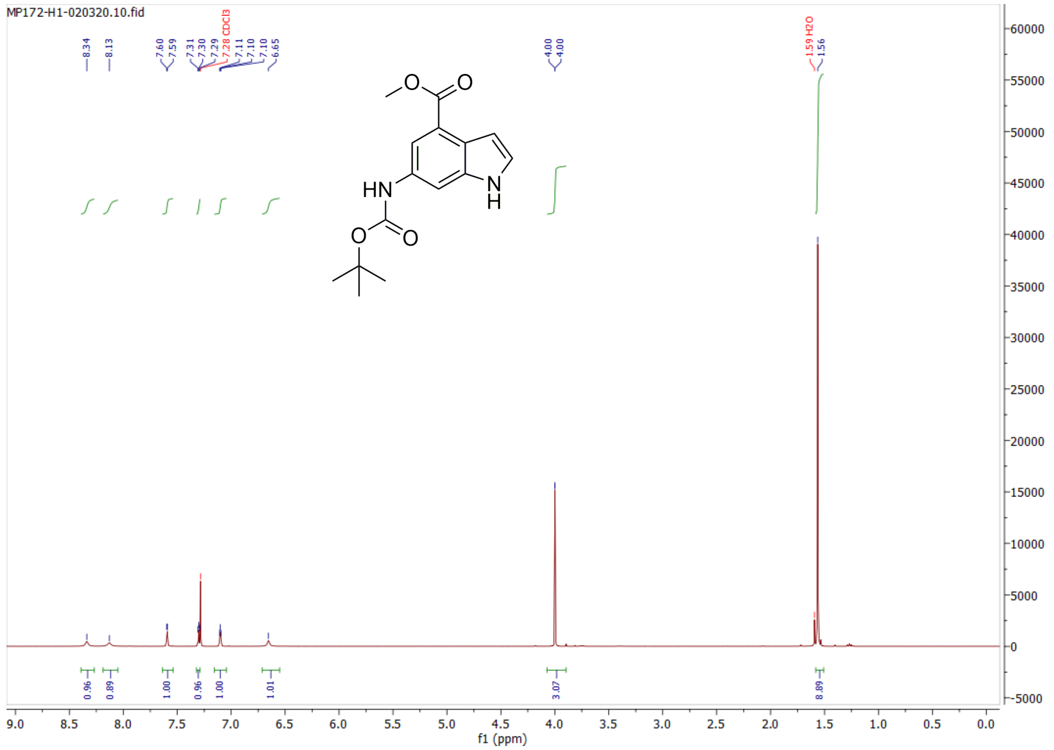

Figure S43. ^{1}H-NMR of compound S1

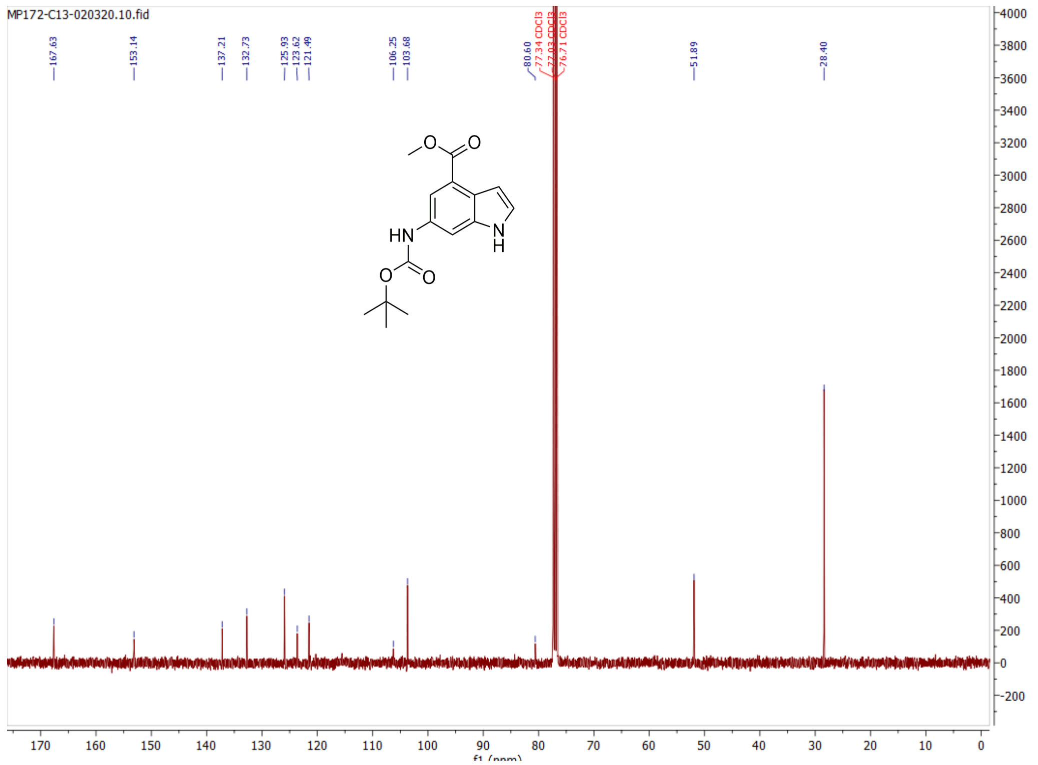

Figure S44. ^{13}C-NMR of compound S1

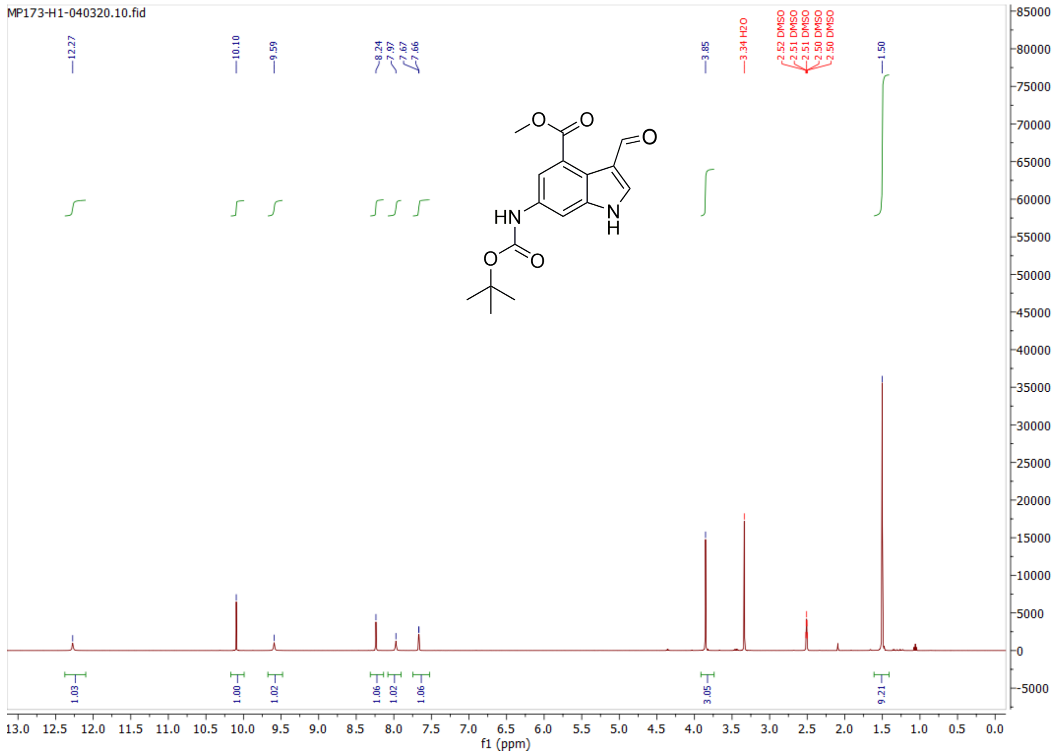

Figure S45. ^{1}**H-NMR of compound S2**

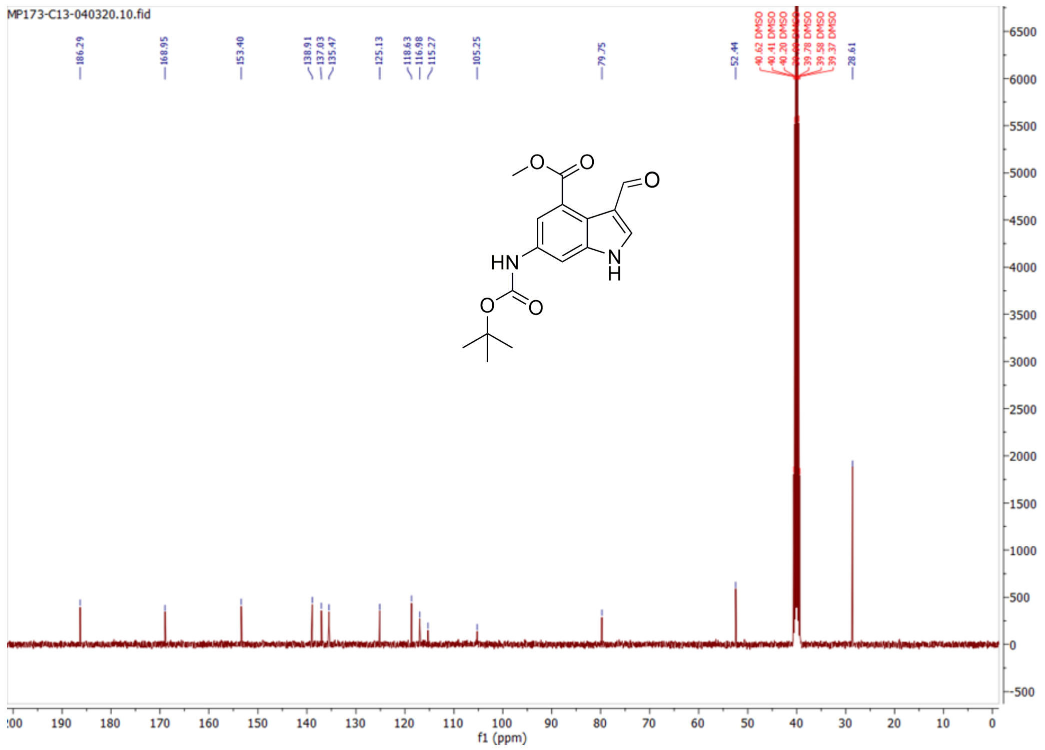

Figure S46. ^{13}**C-NMR of compound S2**

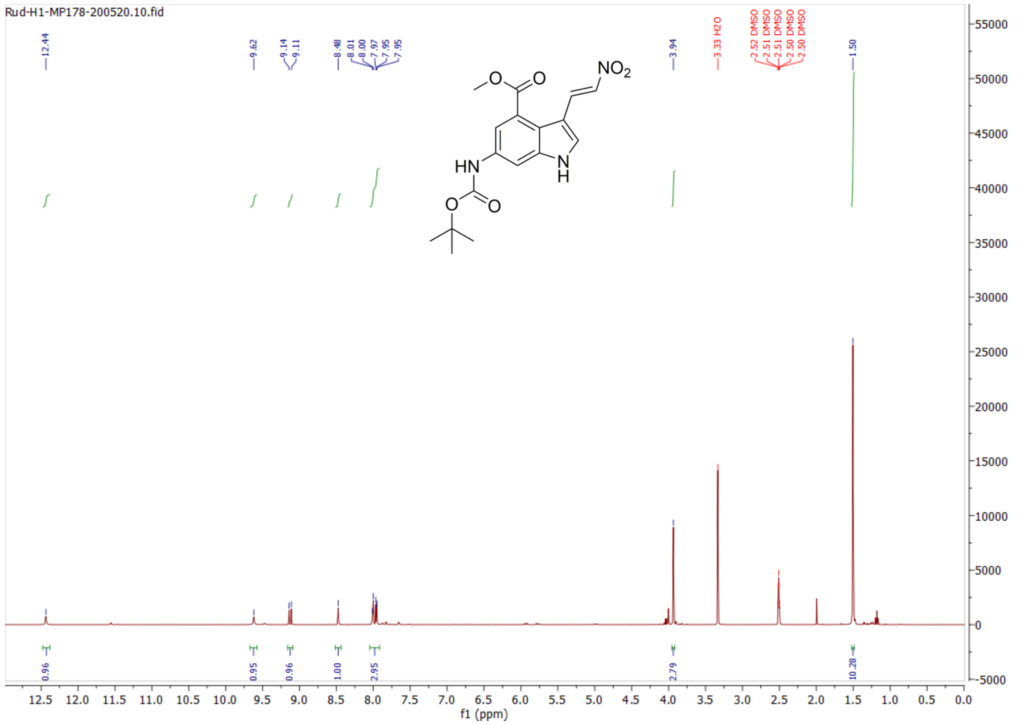

Figure S47. ^{1}H-NMR of compound S3

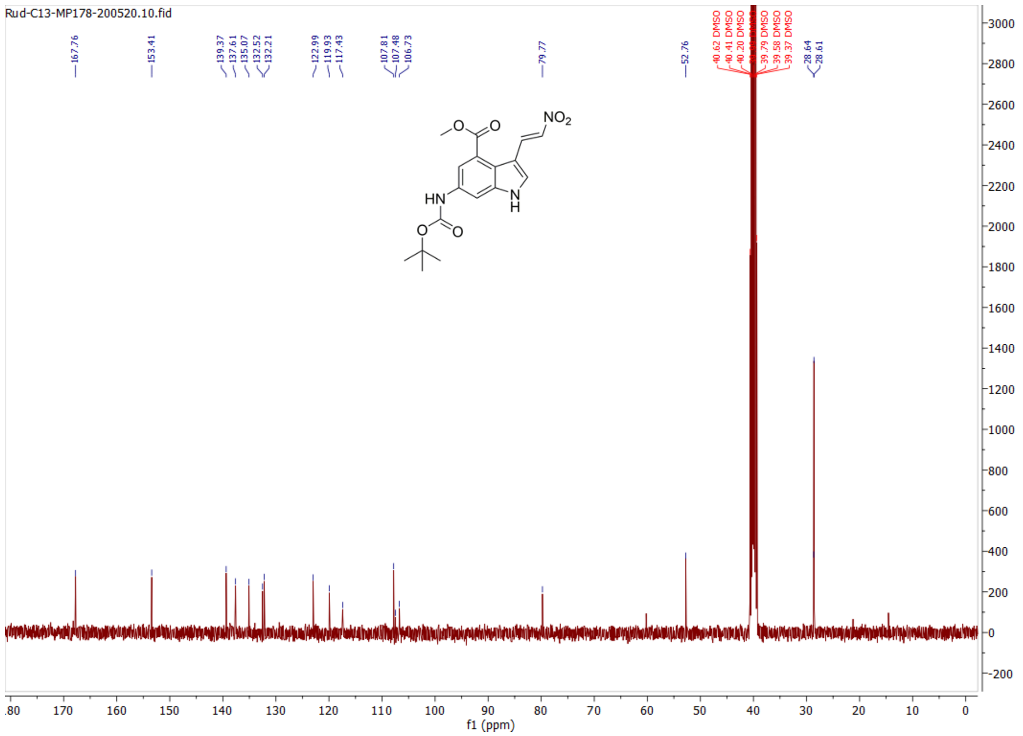

Figure S48. ^{13}C-NMR of compound S3

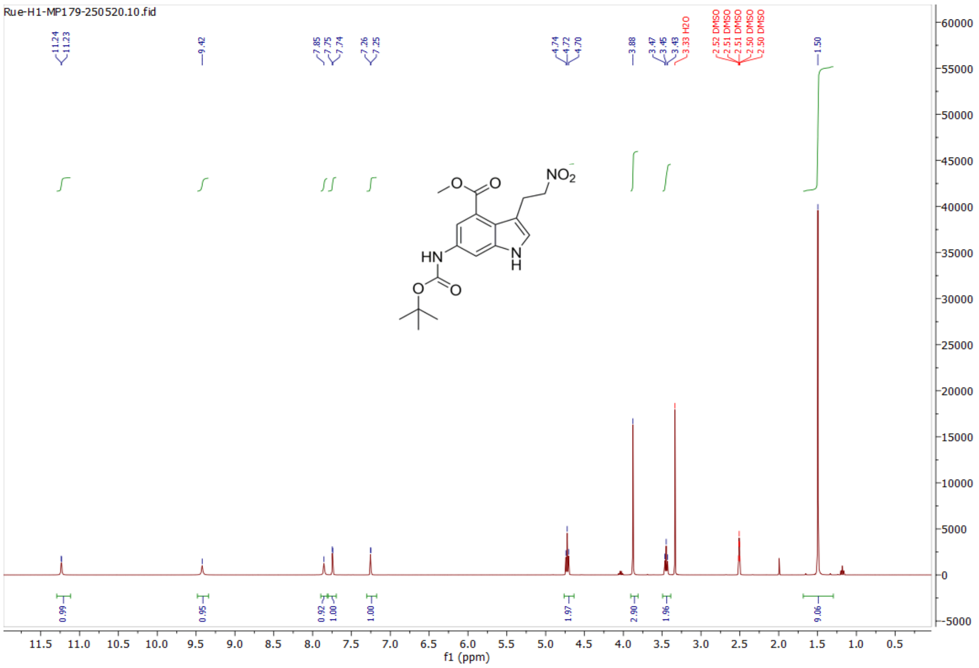

Figure S49. ^{1}H-NMR of compound S4.

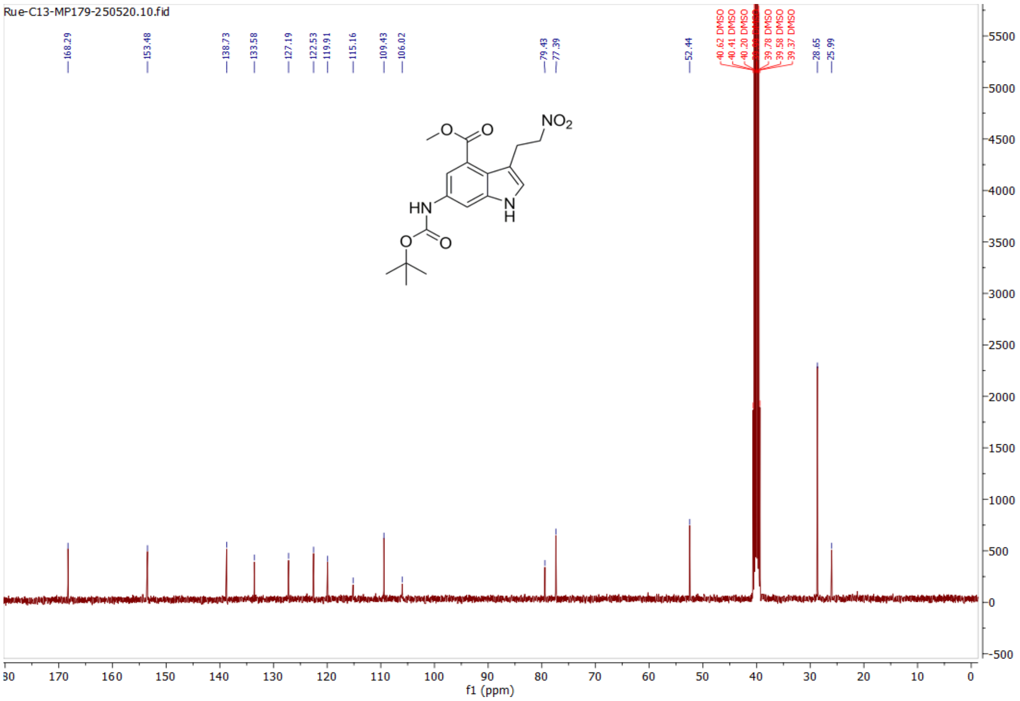

Figure S50. ^{13}C-NMR of compound S4.

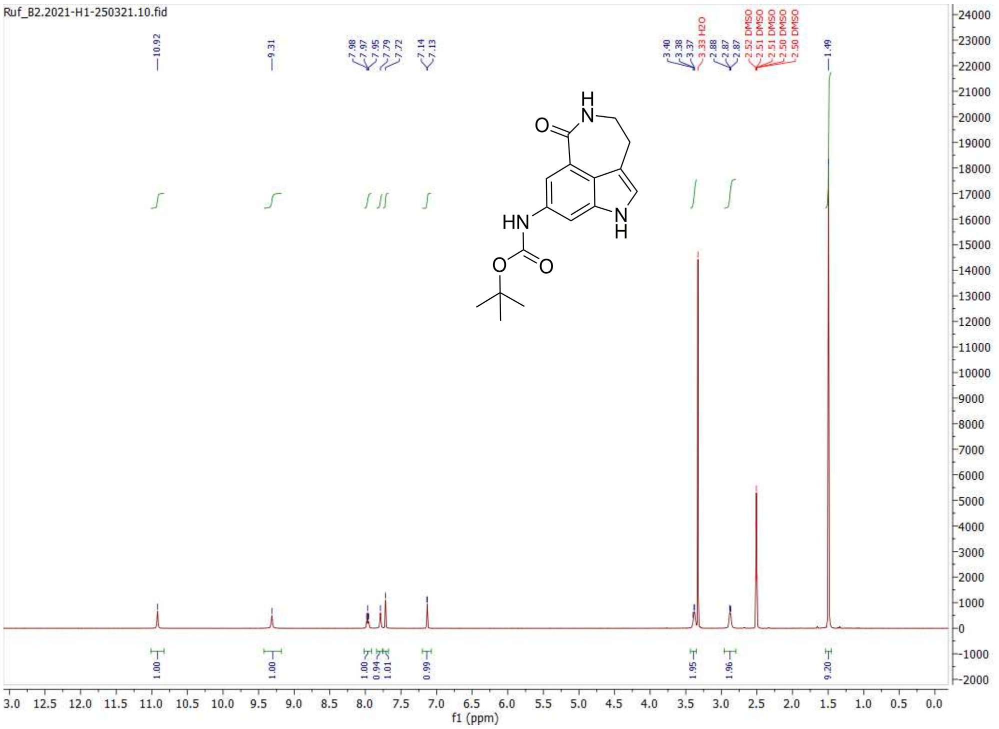

Figure S51. ^{1}H-NMR of compound S5.

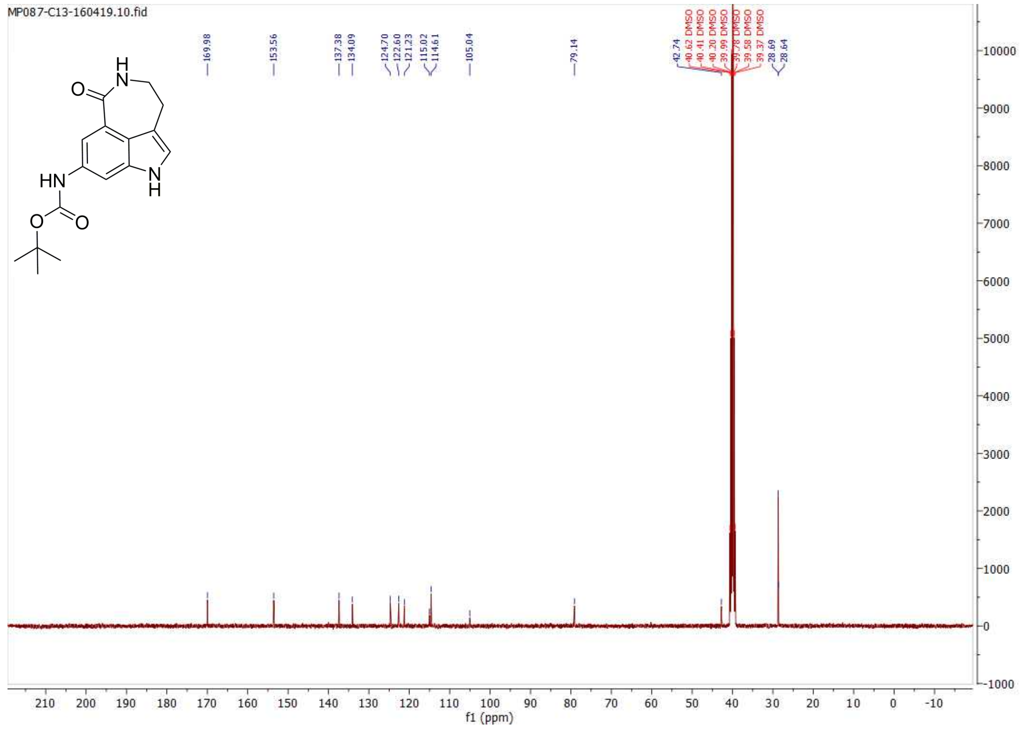

Figure S52. ^{13}C-NMR of compound S5.

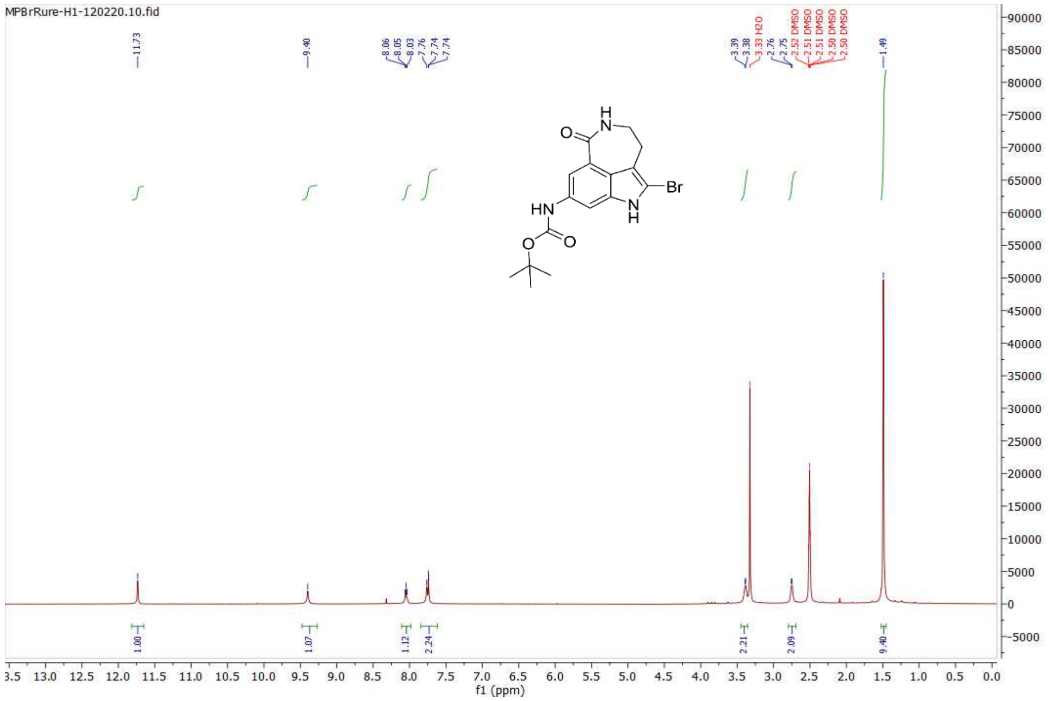

Figure S53 ^{1}H-NMR of compound S6.

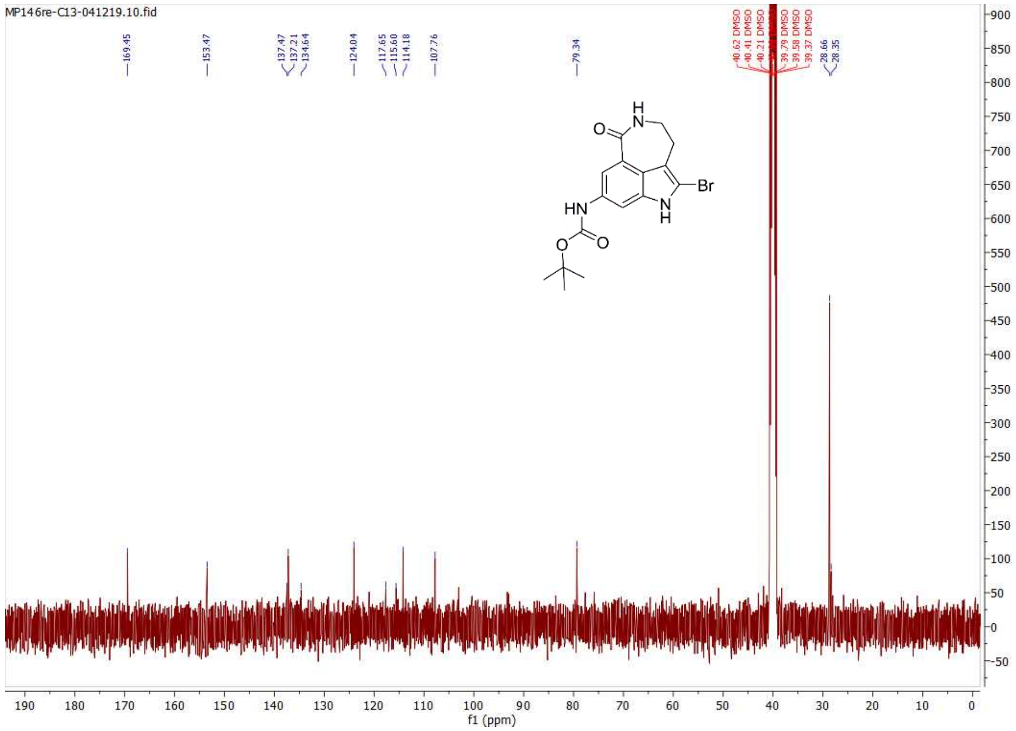

Figure S54. ^{13}C-NMR of compound S6.

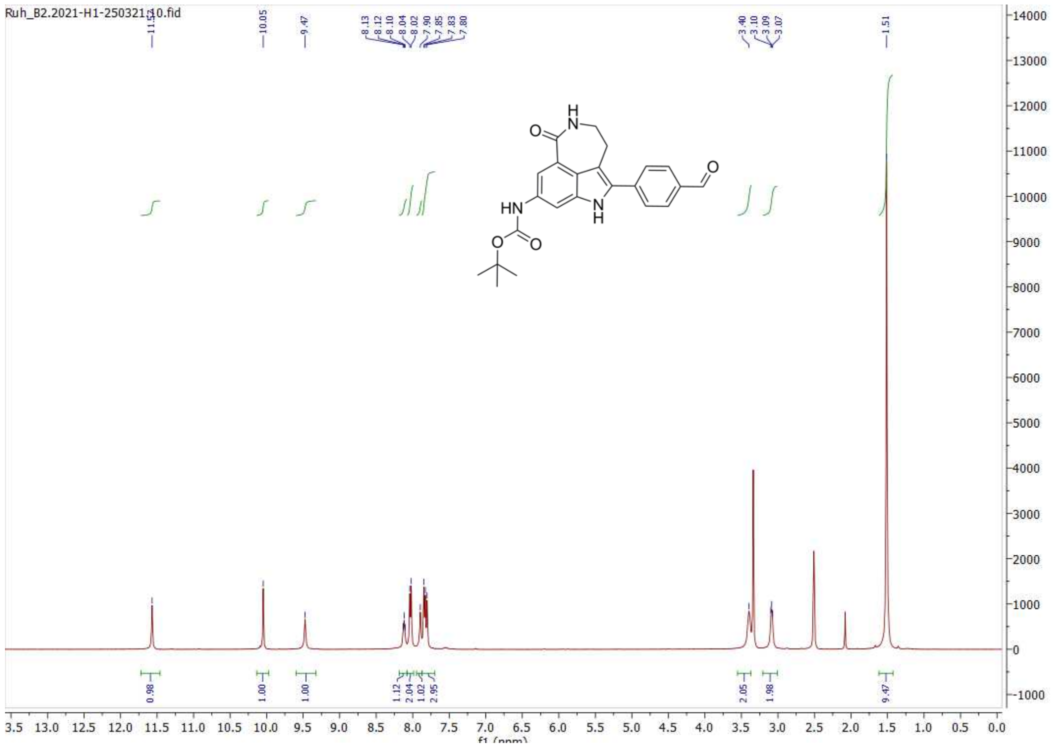

Figure S55. **^{1}H-NMR of compound S7.**

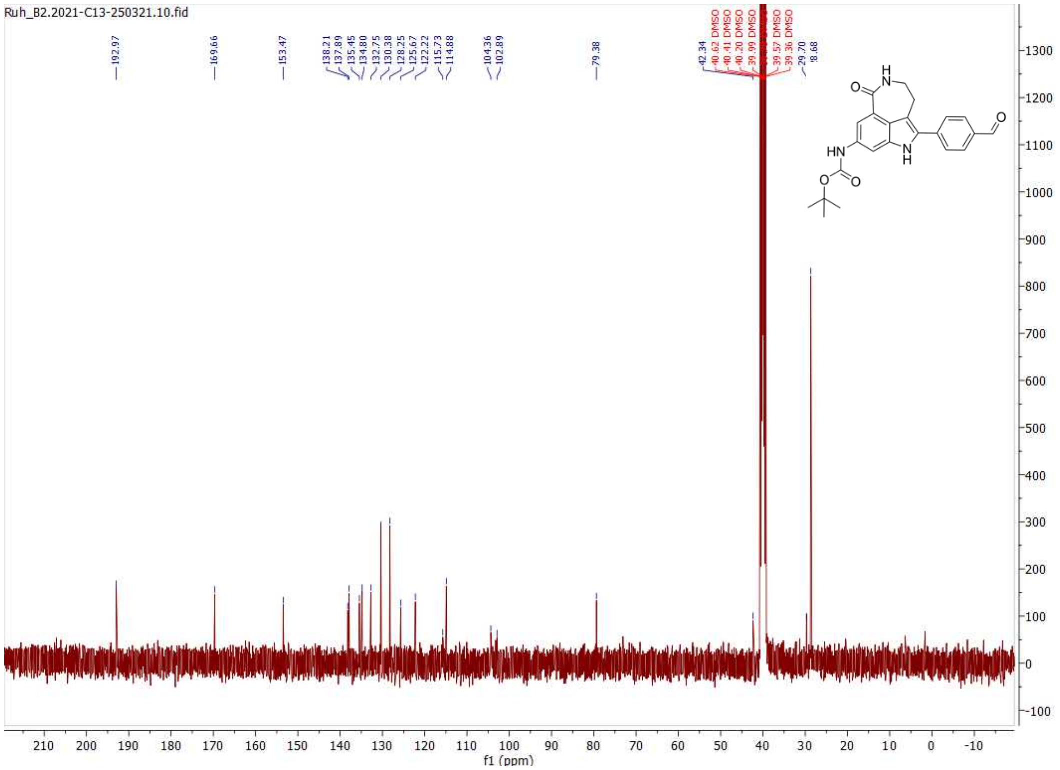

Figure S56. **^{13}C-NMR of compound S7.**

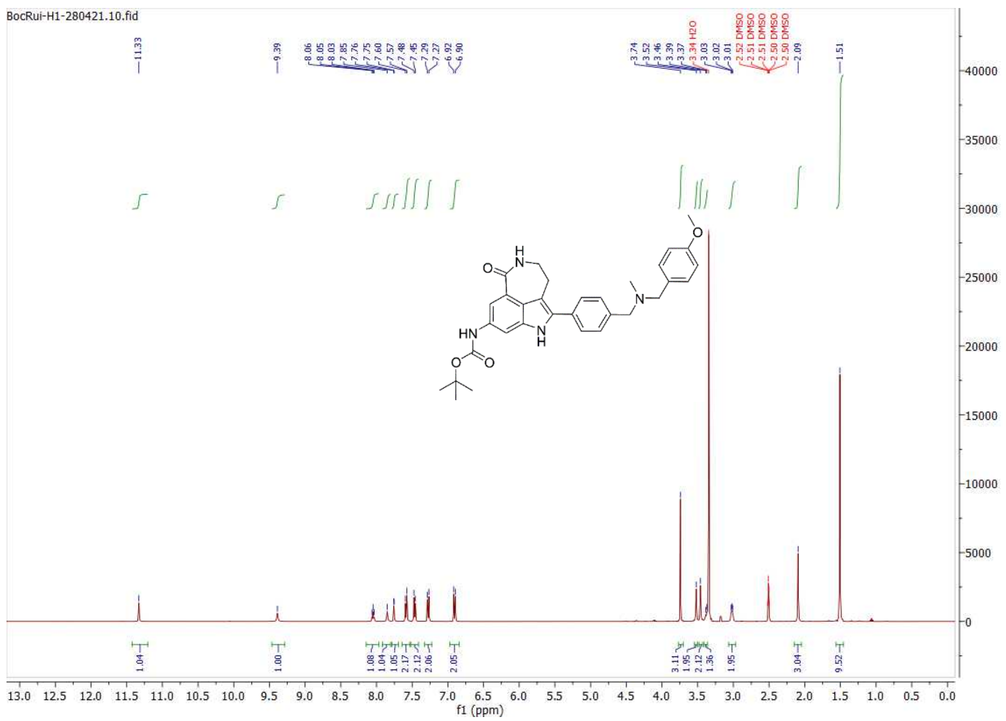

Figure S57. ^{1}H-NMR of compound S8.

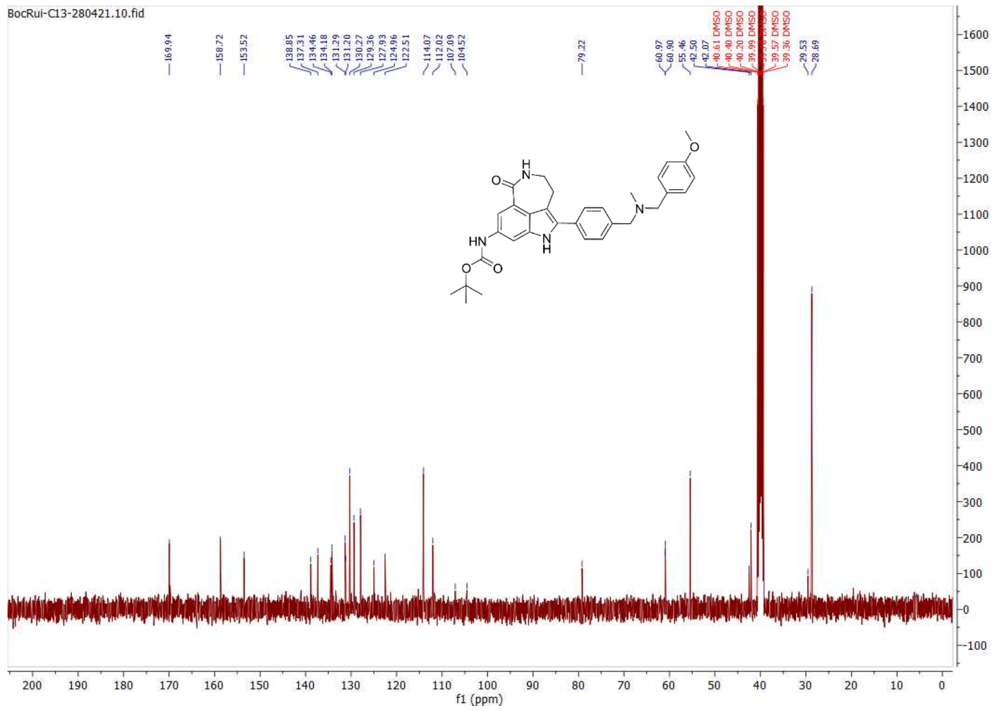

Figure S58. ^{13}C-NMR of compound S8.

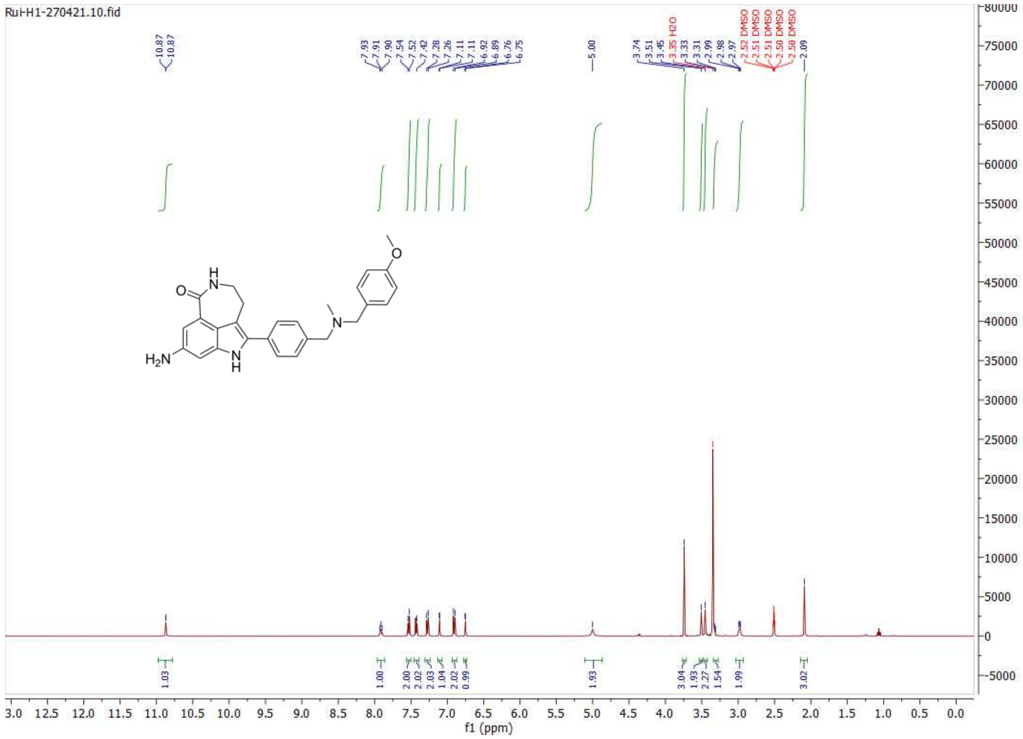

Figure S59. ^{1}H-NMR of compound S9.

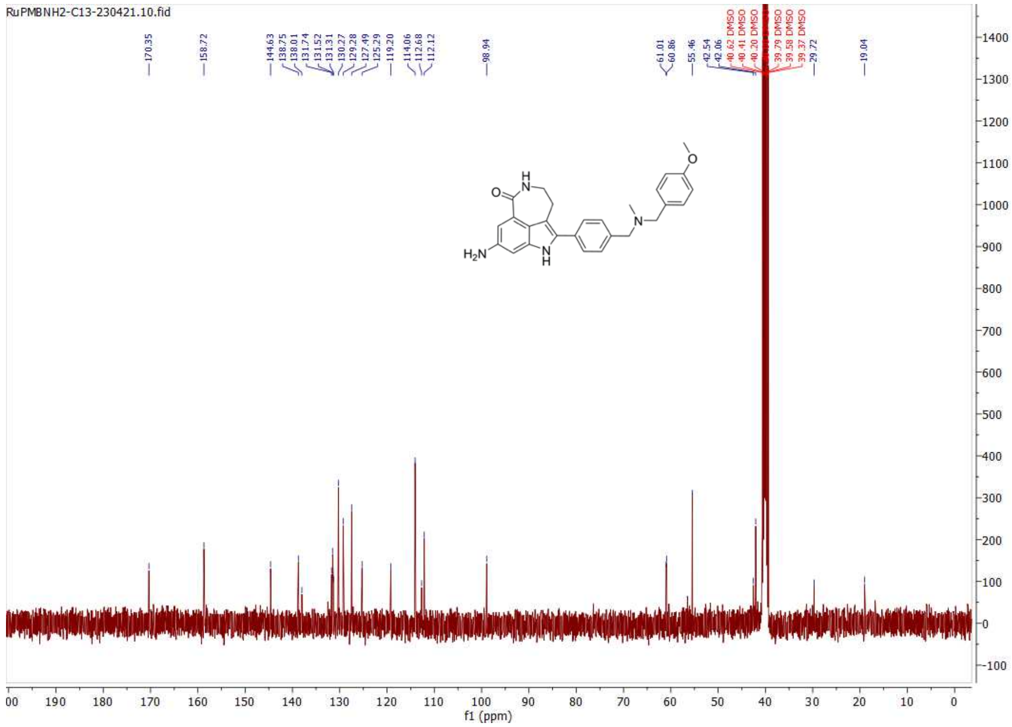

Figure S60. ^{13}C-NMR of compound S9.
